# Alcohol use and its associations with mental health across the first year of the COVID-19 pandemic

**DOI:** 10.1371/journal.pone.0361854

**Published:** 2026-10-30

**Authors:** Lu Zhang, Bianca Ton, Louise Mewton, Susanne Schweizer

**Affiliations:** 1 School of Psychology, University of New South Wales, Sydney, Australia; 2 Centre for Youth Mental Health, University of Melbourne, Melbourne, Australia; 3 Orygen, The National Centre of Excellence in Youth Mental Health, Melbourne, Australia; 4 The Matilda Centre for Research in Mental Health and Substance Use, The University of Sydney, Sydney, Australia; University of Huelva: Universidad de Huelva, SPAIN

## Abstract

**Background:**

While the COVID-19 pandemic caused major disruptions to social connections and lifestyles, many countries continued the sale of alcohol as an essential commodity. Evidence on change in alcohol use, and its association with mental health and wellbeing during the pandemic is, however, mixed and limited to initial periods of the pandemic. This study aimed to address these research gaps.

**Methods:**

Data were drawn from the COVID-19 Risks Across the Lifespan (CORAL) Study, a three-wave longitudinal study conducted between May 2020 and April 2021. The present sample included 2,369 participants (age_*M*_ = 38.81 ± 16.94 years, range = 11–100; 90% females) from the United Kingdom, United States, and Australia. Measures included pre-pandemic alcohol use and changes in consumption frequency, quantity, drinking behaviours (e.g., drinking earlier in the day), and motivations (e.g., drinking for enjoyment) during the pandemic. General perceived stress, COVID-19-related stress, loneliness, and symptoms of depression and anxiety were also assessed.

**Findings:**

Drinking frequency and quantity remained stable across the first year of the pandemic; however, non-linear age associations were found. Young individuals aged between 11 to 20 years, and mid-aged individuals aged between 50 to 70 years, had the highest probability of increased alcohol use frequency and quantity. Drinking behaviours and motivations also remained largely stable, although age- and country-specific differences emerged, potentially reflecting policy differences such as alcohol access restrictions and lockdown measures. Finally, loneliness unidirectionally predicted subsequent alcohol use quantity.

**Conclusions:**

These findings highlight the heterogeneous alcohol use patterns across age groups and countries during the pandemic, and underscore loneliness as an important public health issue with implications for alcohol misuse.

## Introduction

The global coronavirus (COVID-19) pandemic was an unprecedented time of increased public health policy restrictions to reduce disease transmission [1–3]. This led to detrimental impacts on physical and mental health by disrupting people’s social interactions, financial stability, and lifestyles [4]. However, the type and extent of restrictions varied greatly across countries, states, and regions. One example of cross-country policy variation was the provision of alcohol to the public. While some countries banned alcohol sales completely during lockdown or restricted on-premises consumption sites, many countries continued to permit the sale of alcohol as an essential commodity [5]. This is noteworthy because alcohol consumption has been shown to increase with psychological distress [6]. The distress evoked by the pandemic, social isolation, and resulting loneliness may have then led to more alcohol consumption in countries with unrestricted alcohol sales [7]. Elucidating how alcohol use patterns differ across countries can provide insights into the alcohol-mental health relationship. These insights can inform future prevention, intervention, and policy strategies to improve public health outcomes across physical and mental health.

During the pandemic, there were concerns about a “*toll of increased alcohol harm for a generation*” with rapid increases in alcohol sales during the first COVID-19 lockdown [8]. However, review of the empirical evidence shows mixed findings for changes in the quantity and frequency of alcohol use from before to during the COVID-19 pandemic, with reports of increases, decreases, and no change [9–11]. The heterogenous findings may relate to country-level differences. For example, while decreases in alcohol consumption were observed in Australia, increases were observed in the United States and the United Kingdom [12–14]. These findings may be due to alcohol-related policy differences, such as relaxed off-premise sale of alcohol in the United States and United Kingdom but not in Australia [15–17]. Further, pre-existing cross-national differences in alcohol consumption should be considered. According to the World Health Organisation’s 2018 report, total pure alcohol consumption per-capita was higher in the Unites Kingdom and Australia than the United States, at 11.4, 10.6, and 9.8 litres per year, respectively [18]. The prevalence of heavy episodic drinking was also higher in Australia (36%) than in the United Kingdom (30%) and United States (26%) [18]. These data show clear evidence of pre-pandemic geographic differences in alcohol use.

Beyond country-level policy differences, individual-level characteristics such as pre-pandemic level of alcohol use and age may also explain heterogeneity in pandemic-related alcohol use. Indeed, evidence suggests that individuals who were heavy drinkers before the pandemic, defined as drinking more than 4–5 standard drinks per day [19], were more likely to increase their alcohol consumption during the pandemic [20,21]. Contrastingly, light-to-moderate drinkers, who drank 1–3 standard drinks per day before the pandemic [22,23], showed patterns of reduced use [20,21]. Further, alcohol consumption generally increased with age during the pandemic, although this pattern was not uniformly found, yet evidence from underage youth remains limited [10]. Given the high prevalence of underage drinking across the globe, research is needed to understand the impact of the pandemic on alcohol consumption across the lifespan, including among underage individuals [24]. Although the legal drinking age varies across countries, with a minimum age of 18 years in Australia and the United Kingdom and 21 years in the United States, underage alcohol use remains a significant public health concern regardless of its legal status [24,25]. Understanding how pandemic-related disruptions, such as school closures and changes in parental supervision, may have influenced underage drinking is therefore critical to advance age-appropriate prevention and policy responses.

Shifts in drinking motivations (e.g., boredom, increased stress, and distress), and drinking behaviours have also been reported [26–29]. For example, some individuals began drinking earlier in the day, a behaviour potentially facilitated by policy changes such as work from home [29]. Notably, prior research has largely examined drinking during the early stages of the COVID-19 pandemic with limited longitudinal investigation of changes in alcohol use quantity, frequency, other behaviours and motivations of use across the pandemic [9,10]. Predictions based on past global economic crises suggest that the impact of crises on alcohol consumption varies over time. Specifically, alcohol consumption in the medium- and long-term (e.g., months and years after the crisis) has been proposed to increase as distress continues to build [30]. Hence, it is unclear whether COVID-19 effects on drinking pattern and habits persisted following the easing of restrictions. It is similarly unclear whether this varied across age groups who may have been differentially impacted by the pandemic’s social restrictions.

In addition to the direct impact of restrictions on alcohol access, there may also have been indirect effects. Specifically, increased alcohol use during the COVID-19 pandemic has been associated with poorer mental health and wellbeing, as well as increased stress and loneliness [14,31–33]. In a sample of more than 5,000 adults in the United States and another sample of >13,800 Australian adults, individuals with anxiety or depressive symptoms had significantly higher odds of reporting increased alcohol use compared to those without these symptoms during the pandemic [33,34]. However, limited research has directly examined the association between alcohol use and COVID-19-specific stress. Existing evidence is also primarily based on cross-sectional research. As a result, the directionality of these relationships remains unclear, and little is known about how these associations may have changed over the course of the pandemic.

The present study therefore aimed to address the gaps in the literature by using data from the COVID-19 Risks Across the Lifespan (CORAL) study. Specifically, we investigated age- and country-related (Australia, the United Kingdom, and United States) differences in alcohol use, and their associations with mental health problems across the first year of the COVID-19 pandemic (May 2020 to April 2021). The longitudinal design tested the following hypotheses: individuals who drank more pre-pandemic would report greater alcohol use at T1 (H1); alcohol use would increase over time (H2a), drinking behaviours such as drinking earlier in the day would change (H2b), especially in older adults (H2c). We also hypothesised that the most common motivations for alcohol use would be to reduce stress or worry and to kill time or reduce boredom (H3), and that alcohol consumption would show bidirectional associations with mental health, stress, and loneliness (H4).

## Methods

### Participants

Participants were drawn from the CORAL study, a prospective cohort study conducted in Australia, the United Kingdom, and United States. The study assessed determinants of mental health and cognition during the COVID-19 pandemic. Participants were assessed at three timepoints in three-month intervals across the first year of the COVID-19 pandemic. Specifically, baseline assessments were completed between 5 May to 30 September 2020 (T1), 3-month follow-up assessments between 5 August 2020 to 31 January 2021 (T2); and 6-month follow-up assessment from 5 November 2020 to 30 March 2021 (T3).

The CORAL study used convenience sampling, primarily recruiting participants online via research forums (e.g., MQ Participate, SANE Australia), social media (e.g., Facebook, Instagram), and newsletters (e.g., school newsletters, general and youth mental health organisations). Sample size was determined by recruitment capacity within the study’s data collection period. Eligibility criteria included: i) aged 11 years or older; ii) residence in Australia, the United Kingdom, or United States; iii) English fluency, iv) no history of traumatic brain injury or neurodevelopmental or neurological disorders, as the broader study included a cognitive task [35], and v) capable of providing informed consent. The full sample comprised 3,208 participants who consented to and participated in the study.

The present study further excluded participants with missing data on age (*N* = 40) and the question whether they drink alcohol or not at T1 (*N* = 799), resulting in 2,369 total participants (age *M* = 38.81 years, *SD* = 16.94, range = 11–100; 90% females). As the broader CORAL Study specifically targeted recruitment of pregnant individuals, the present sample also included 114 pregnant participants.

The study was approved by the University of New South Wales Human Research Ethics Committee (HC200287). Electronic informed consent was obtained from all participants. For participants younger than 18 years, electronic assent was obtained from the participants, together with electronic consent from a parent or guardian. Participants under 18 years were able to participate only after both forms of consent had been obtained.

### Procedure

Eligible participants who provided informed consent online were invited to complete the online survey hosted on Qualtrics and a cognitive task hosted on the Cognitron platform. The cognitive task was described elsewhere and is not relevant to the present study [35]. Participants completed demographic questions before reporting on their mental health, alcohol use, stress and social determinants of health. Participants who completed at least 65% of the baseline survey (T1) were followed up at T2 and T3. Participants who completed the survey at T2 and T3 received an Amazon gift card valued at £5, USD $6, or AUD $10. Additionally, every 100th participants received an Amazon gift card valued at £50, USD $60, or AUD $100.

### Measures

The following self-report measures were administered at all timepoints, except for demographic information and pre-pandemic alcohol consumption which were only measured at T1.

#### Alcohol use.

Participants were first asked to indicate whether they drink alcohol (i.e., ‘*Do you drink alcohol’*) with a binary yes or no response option at each timepoint. If they indicated they do not drink alcohol, the following items were not presented. Pre-pandemic alcohol consumption frequency and quantity over the past year was assessed at T1 using the Alcohol Use Disorder Identification Test [AUDIT; 36]. Specifically, the first two items from the AUDIT were used (i.e., ‘*Before COVID-19 how often did you have a drink containing alcohol’* and ‘*Before COVID-19 how many standard drinks containing alcohol did you have on a typical day’*). A standard drink was defined as half a pint of beer (284 ml) or a small glass of wine (100 ml) for the participants. Responses were provided on a 5-point Likert scale, ranging from ‘*never’* (0) to ‘*4 or more times a week’* (4) for the frequency item and ‘*1 or 2’* (0) to *‘10 or more’* (4) standard drinks for the quantity item. At each timepoint, participants also reported changes in alcohol use frequency and quantity relative to pre-pandemic levels using two items. In these items, “now” referred to alcohol use at the time of the assessment: ‘*How frequently are you drinking alcohol now compared to before COVID-19’* and ‘*What quantity are you drinking alcohol now compared to before COVID-19’*. Responses were rated on a 5-point Likert scale: *‘lot less’* (−2)*, ‘a bit less’* (−1)*, ‘about the same’* (0)*, ‘a bit more’* (1)*,* or ‘*a lot more’* (2) [37]. Changes in drinking behaviours and motivations relative to pre-pandemic were assessed using binary (yes or no) items. For example, ‘*Compared to before the COVID-19 pandemic, are you now starting to drink alcohol earlier in the day’*). Behaviours assessed included drinking earlier in the day, having difficulty stopping drinking, and drinking alone. Motivations assessed included drinking to reduce boredom, to destress, or to feel good or enjoy oneself.

#### COVID-19 related stress.

The Pandemic Anxiety Scale (PAS) was administered to assess COVID-19-related stress [38]. Participants endorsed 9 items, such as ‘*I’m worried that I will catch COVID-19’,* on a 5-point Likert scale from ‘*strongly disagree’* (0) to ‘*strongly agree’* (4). The scale showed acceptable internal consistency at all timepoints (*ωT* = 0.83–0.86).

#### General stress.

The Perceived Stress Scale (PSS) was administered to measure perceived level of general stress [39]. Participants endorsed 4 items on a 5-point Likert scale from ‘*never’* (0) to ‘*very often’* (4). The scale had acceptable internal consistency at all timepoints (*ωT* = 0.84–0.85).

#### Loneliness.

The 20-item UCLA Loneliness Scale was administered to assess loneliness and social isolation [40]. Participants endorsed items on a 4-point Likert scale from ‘*I never feel this way’* (0) to ‘*I often feel this way’* (3). The scale showed excellent internal consistency at all timepoints (*ωTs* = 0.97).

#### Depression.

The eight-item Patient Health Questionnaire-8 (PHQ-8) was used to assess the severity of depression symptoms [41]. Participants endorsed items on a 4-point Likert scale from ‘*not at all’* (0) to ‘*nearly every day’* (3). The scale showed good internal consistency at all time points (*ωT* = 0.93–0.94).

#### Anxiety.

The seven-item Generalised Anxiety Disorder-7 (GAD-7) scale was administered to assess severity of generalised anxiety symptoms [42]. Participants endorsed items on a 4-point Likert scale from ‘*not at all’* (0) to ‘*nearly every day’* (3). The scale showed excellent internal consistency at all time points (*ωT* = 0.95–0.96).

### Statistical analysis

Analyses were performed using R version 4.4.0 [43]. See supplementary materials (SM) for model details. All participants, including those younger than 18 years, were included in analysis because the study aimed to examine age-related differences. Missing data were handled via complete-case analysis throughout. Participants were excluded from a given model if they had missing data on any included variable, whether due to non-response or because they had indicated they do not drink alcohol and were therefore not presented with subsequent alcohol-related items.

Separate cumulative link ordinal regressions models with logit link functions were conducted, to examine associations between pre-pandemic alcohol use quantity and frequency and reported changes in alcohol use at T1 (H1). Age and country variations in pre-pandemic alcohol use were also examined using ordinal regression with age and country as predictors. Reported changes in alcohol use across T1-T3 (H2a) and age effects (H2c) were examined using ordinal mixed effects models with logit link functions. Predictors included categorical timepoint categorical, continuous time-invariant baseline standardised age, and their interaction. Linear, quadratic, and cubic age terms were compared using AIC and log-likelihood, with cubic term providing the best model fit and therefore retain in the final models (see S1 Table 3 in S1 File for model comparison results). Where the timepoint by age interaction was non-significant, results from the reduced model (without interaction) were reported.

Changes in drinking behaviours (H2b) and motivations (H3) across T1-T3 were analysed using mixed-effects logistic models with timepoint, time-invariant baseline age, and their interaction; non-linear age effects were not tested due to convergence issues. Pre-pandemic to T1 changes were examined using logistic regression with age and country as predictors. False discovery rate (FDR) correction was applied across the three models within H2b and H3 [44].

Cross-lagged panel models assessed bidirectional associations of alcohol use frequency and quantity with general stress, COVID-related stress, loneliness, and symptoms of depression and generalized anxiety (H4). Outcomes were modelled separately and robust maximum likelihood estimation was used.

Country was included as a categorical covariate in all models, and participant ID was included as a random effect where appropriate.

#### Sensitivity analysis.

To assess whether any observed effects were over and above COVID-related stress effects, the total score on the Pandemic Anxiety Scale was included as a covariate in sensitivity analyses for H1-H3. Sensitivity analyses excluding pregnant subsample were conducted for all hypotheses to assess finding robustness.

## Results

### Demographic information and sample distribution

See Table 1 for demographic information and clinical characteristics, and Table 2 for the distribution of alcohol use quantity and frequency across waves. The distribution of alcohol use quantity and frequency by gender and country is reported in S1 Table 1 and 2 in S1 File, respectively.

**Table 1:** Demographics information.

Variable	*M* (SD) / *N* (%)
Age (at T1)	38.81 (16.94) years
Gender	
Female	2,130 (90%)
Male	212 (8.9%)
Other	21 (0.9%)
Prefer not to say	6 (<1%)
Country of residence	
United Kingdom	1,073 (45%)
United States	702 (30%)
Australia	594 (25%)
Missing	
Ethnicity	
Caucasian	1,997 (84%)
Asian	104 (4%)
Mixed	71 (3%)
Hispanic	44 (2%)
African	16 (<1%)
Aboriginal or Torres Strait Islander	9 (<1%)
Other	97 (4%)
Prefer not to say	30 (1%)
Highest level of education	
Primary school	21 (1%)
High school	463 (20%)
Professional or Vocational training	370 (16%)
University	1,515 (64%)
COVID-19 stress	
T1	19.82 (6.17)
T2	19.10 (6.47)
T3	19.09 (6.39)
Perceived general stress	
T1	7.89 (3.52)
T2	7.22 (3.54)
T3	7.54 (3.51)
Loneliness	
T1	25.60 (15.71)
T2	25.69 (15.98)
T3	27.57 (15.77)
Generalised anxiety symptoms	
T1	8.15 (6.17)
T2	6.73 (5.83)
T3	7.37 (5.99)
Depression symptoms	
T1	9.37 (6.31)
T2	8.06 (6.27)
T3	8.47 (6.31)

T1 = baseline, T2 = 3-months follow-up, T3 = 6-months follow-up, *M* = mean, SD = standard deviation. ANOVA revealed no significant variation in age across countries, *F*(2, 2366) = 1.83, *p* = .160 (age mean: United Kingdom = 38.1 ± 16.6, United States = 39.3 ± 18.1, Australia = 39.6 ± 16.2 years).

**Table 2:** Distribution of alcohol use quantity and frequency across assessment timepoints.

Variable	N (%)			
	Pre-pandemic	T1	T2	T3
Indicated alcohol use				
Yes	–	1,158 (49%)	476 (56%)	466 (60%)
No	–	1,211 (51%)	373 (44%)	310 (40%)
Alcohol use quantity				
Absolute levels (pre-pandemic)				
1-2 std drinks	890 (79%)	–	–	–
3–4 std drinks	164 (15%)	–	–	–
5–6 std drinks	52 (5%)	–	–	–
7–9 std drinks	16 (1%)	–	–	–
10 or more std drinks	6 (1%)	–	–	–
Changes over time (relative to pre-pandemic levels; T1-T3)				
A lot less	–	237 (21%)	78 (16%)	83 (18%)
A bit less	–	113 (10%)	68 (14%)	63 (14%)
About the same	–	500 (43%)	214 (45%)	213 (46%)
A bit more	–	237 (21%)	91 (19%)	80 (17%)
A lot more	–	64 (6%)	25 (5%)	23 (5%)
Alcohol use frequency				
Absolute levels (pre-pandemic)				
Never	21 (2%)	–	–	–
Monthly or less	320 (28%)	–	–	–
2-4 times per month	373 (32%)	–	–	–
2-3 times per week	285 (25%)	–	–	–
More than 4 times a week	154 (13%)	–	–	–
Changes over time (relative to pre-pandemic levels; T1-T3)				
A lot less	–	227 (20%)	73 (15%)	82 (18%)
A bit less	–	105 (9%)	65 (14%)	66 (14%)
About the same	–	403 (35%)	196 (41%)	187 (40%)
A bit more	–	319 (28%)	104 (22%)	97 (21%)
A lot more	–	97 (8%)	38 (8%)	30 (6%)

Percentages are calculated using non-missing responses (minor deviations due to rounding). T1 = baseline, T2 = 3-months follow-up, T3 = 6-months follow-up.

More than half the sample reported not drinking alcohol at T1 and were therefore not included in the subsequent analyses, as the questions pertaining to alcohol use were not asked of them. Of these individuals 490 (40%) were pregnant people and 278 (23%) were individuals under their country-specific legal age of drinking (18 years in Australia and the United Kingdom; 21 years in the United States).

### Alcohol use quantity and frequency

#### Pre-pandemic use.

At T1, 49% of participants with valid responses reported alcohol use and were therefore presented with questions about their pre-pandemic alcohol use. Among these participants, older individuals were more likely to report significantly higher drinking quantity (odds = 1.13, 95% CI = [1.04, 1.22], *p* = .004) and more frequent drinking (odds = 1.95, 95% CI = [1.83, 2.08], *p* < .001) than younger individuals before the outbreak (Fig 1). Relative to Australia, participants in the United Kingdom were more likely to report greater drinking quantity (odds = 1.36, 95% CI = [1.12, 1.66], *p* = .002) but lower frequency (odds = 0.80, 95% CI = [0.69, 0.93], *p* = .004). Participants in the United States, however, were more likely to report both lower quantity (odds = 0.42, 95% CI = [0.32, 0.55], *p* < .001) and frequency (odds = 0.60, 95% CI = [0.50, 0.71], *p* < .001), compared to those in Australia (see S1 Table 1 in S1 File for proportion distribution by country).

**Fig 1:**
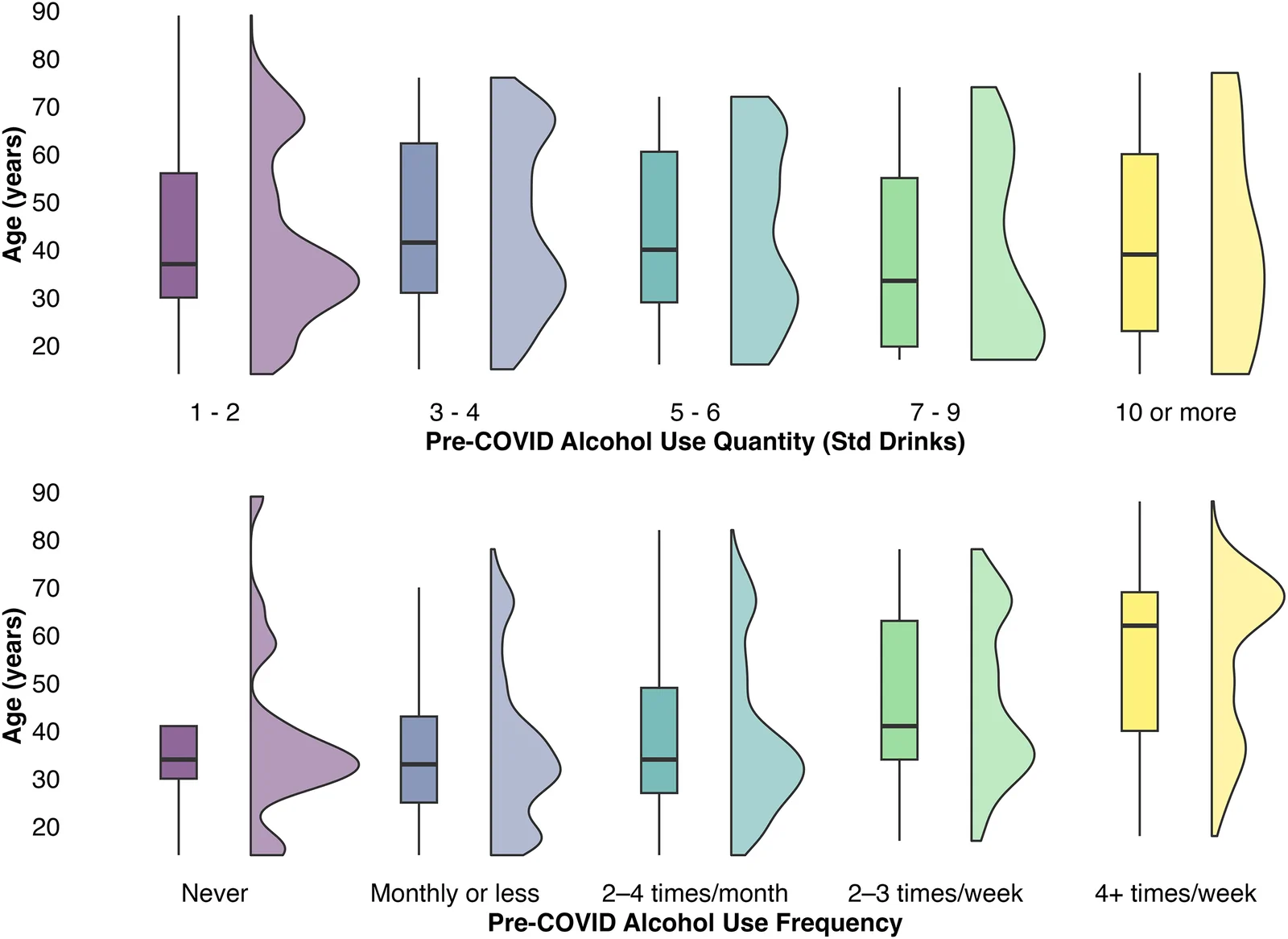
Distribution of pre-pandemic alcohol consumption quantity and frequency by age.

Sample sizes for each group were as follows: for pre-COVID alcohol use quantity (*N* = 1,128 with valid responses), 1–2 drinks (*N* = 890), 3–4 drinks (*N* = 164), 5–6 drinks (*N* = 52), 7–9 drinks (*N* = 16), 10 or more drinks (*N* = 6); for pre-COVID alcohol use frequency (*N* = 1,153 with valid responses), Never (*N* = 21), Monthly or less (*N* = 320), 2–4 times per month (*N* = 373), 2–3 times per week (*N* = 285), 4+ times per week (*N* = 154). std = standard drinks.

#### Changes during the pandemic.

*Pre-pandemic to T1.* Ordinal regression showed that individuals who drank 3–4 standard drinks a day pre-pandemic were significantly more likely to show increased drinking at T1 (odds = 1.40, 95% CI = [1.01, 1.92], *p* = .041), relative to those who drank 1–2 standard drinks. Participants in the United Kingdom (odds = 1.45, 95% CI = [1.12, 1.89]*, p* = .005) and the United States (odds = 1.70, 95% CI = [1.26, 2.30], *p* < .001) were also more likely to report increased drinking, compared to those in Australia.

No association was observed between pre-pandemic and T1 drinking frequency (S1 Table 5 in S1 File). However, compared to individuals in Australia, those in the United Kingdom (odds = 1.67, 95% CI = [1.29, 2.15], *p* < .001) and United States (odds = 1.72, 95% CI = [1.28, 2.31], *p* < .001) were more likely to report more frequent drinking at T1, relative to before the pandemic.

T1-T3. No significant changes in drinking frequency and quantity were observed across T1-T2. However, compared with T1, responses at T3 were more likely to fall within a lower alcohol-frequency change category (Table 3). It is important to note that as odds greater than one from ordinal models indicate a higher likelihood of responding in a higher change category, such as moving from ‘*a lot less*’ to *‘a little less’*. They do not necessarily reflect an increase in absolute alcohol quantity or frequency.

**Table 3:** Results for changes in alcohol use quantity and frequency.

Variable	Odds	95% CI	SE	*t*	*p*
Quantity model					
Age	2.49	[1.74, 3.57]	0.18	4.95	**<.001**
Age^2^	1.62	[1.23, 2.13]	0.14	3.40	**.001**
Age^3^	0.67	[0.57, 0.75]	0.09	−4.45	**<.001**
T2	1.04	[0.81, 1.34]	0.13	0.29	.770
T3	0.82	[0.63, 1.07]	0.13	−1.47	.141
United Kingdom	1.98	[1.29, 3.04]	0.22	3.12	**.002**
United States	2.07	[1.27, 3.36]	0.25	2.93	**.003**
Frequency model					
Age	1.94	[1.40, 2.69]	0.17	3.99	**<.001**
Age^2^	1.31	[1.02, 1.69]	0.13	2.10	**.036**
Age^3^	0.75	[0.64, 0.88]	0.08	−3.51	**<.001**
T2	0.95	[0.75, 1.21]	0.12	−0.42	.674
T3	0.73	[0.57, 0.93]	0.13	−2.52	**.012**
United Kingdom	2.10	[1.41, 3.11]	0.20	3.66	**<.001**
United States	2.01	[1.29, 3.15]	0.23	3.07	**.002**

Odds greater than one reflect a higher likelihood of reporting a higher response category (e.g., from much less to a little bit less), but not necessarily an absolute increase in alcohol use. T1 was the reference category for T2 and T3, and Australia was the reference country. Bold text indicates significant effect at *p* < .05.

Significant non-linear age effects were observed for changes in drinking frequency and quantity (Fig 2). Individuals younger than approximately 75 years had a consistently high probability of remaining at pre-pandemic levels, whereas this probability declined sharply among those older than 75 years, who instead showed an increasing probability of reporting decreases in consumption. Among individuals who did show changes in alcohol use, the highest predicted probability of increased consumption (relative to per-pandemic) was observed among younger (11–20 years) and mid-aged (50–70years) individuals. See S1 Table 5 in S1 File for detailed results.

**Fig 2:**
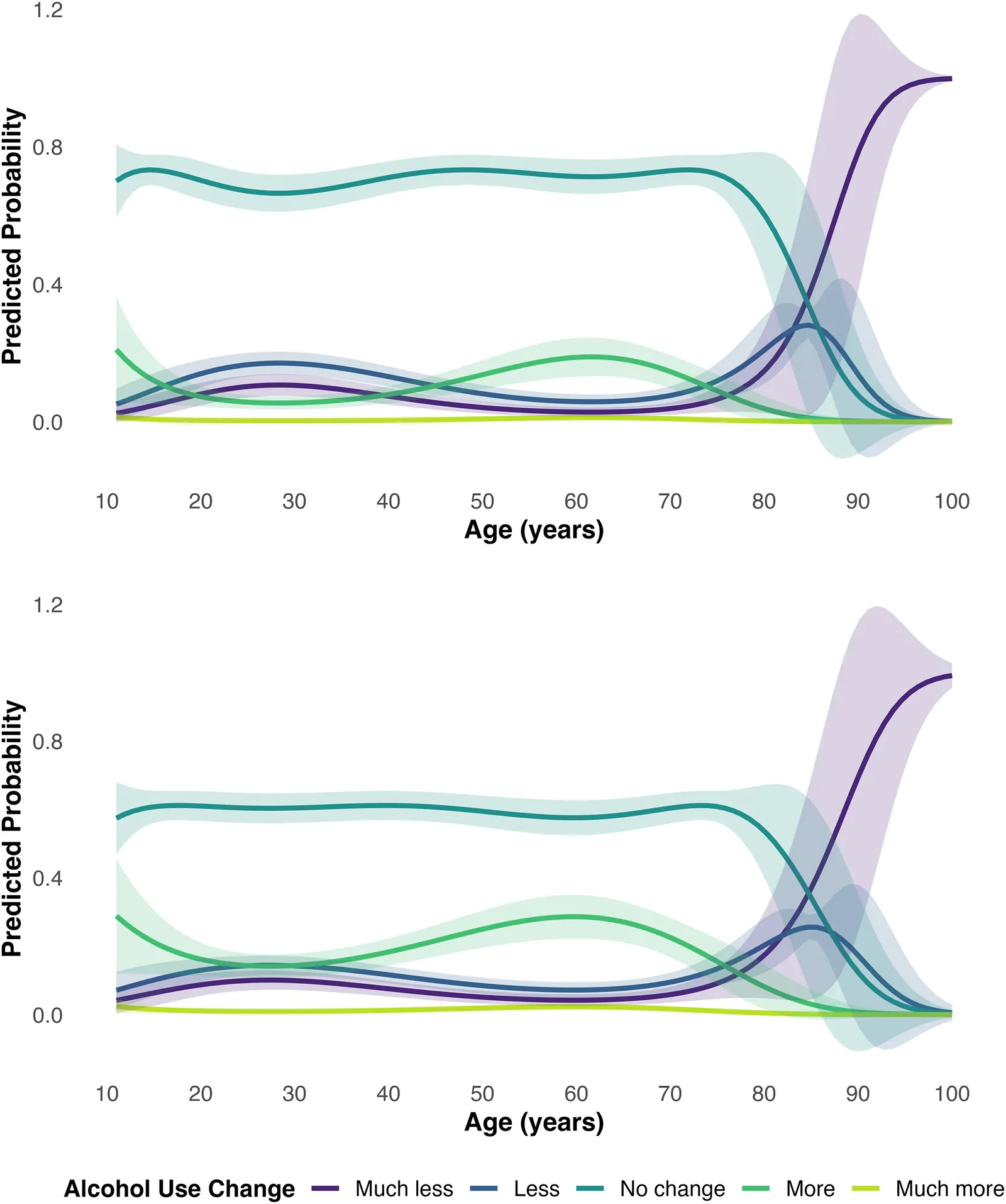
Marginal effect of age on predicted probability of varying levels of change in alcohol use during the pandemic.

The top panel shows predicted probability of change in alcohol use quantity while the bottom panel shows predicted probability of change in alcohol use frequency. Each line represents the predicted probability of being at each response level (much less, a bit less, no change, a bit more, or much more alcohol consumption or frequency relative to pre-pandemic levels) on average across the timepoints, in association with age. Shaded area indicates 95% confidence intervals.

### Alcohol use behaviour

At T1, individuals showed significantly *lower* odds of *reporting increases* in drinking behaviours relative to before the pandemic, These behaviours included starting to drink earlier in the day (odds = 0.16, 95% CI = [0.11, 0.22], *p*FDR < .001), difficulty stopping drinking (odds = 0.12, 95% CI = [0.08, 0.17], *p*FDR < .001), and drinking alone (odds = 0.27, 95% CI = [0.21, 0.36], *p*FDR < .001). Importantly, as these items assessed *increases* only (e.g., ‘*Are you now starting to drink earlier in the day’),* these findings should not be interpreted as evidence that the behaviours decreased. Rather, these findings more likely indicate that participants tended *not* to report greater engagement in these behaviours than before the pandemic. No significant effects of age or country were found for changes in difficulty stopping drinking or drinking alone. For starting to drink earlier in the day, older age was associated with lower odds of reporting a change (odds = 0.79, 95% CI = [0.68, 0.91], *p*FDR = .004). Individuals in the United Kingdom or United States had higher odds of reporting increases in this behaviour at T1 than those in Australia (United Kingdom: odds = 1.85, 95% CI = [1.26, 2.76], *p*FDR = .004; United States: odds = 2.15, 95% CI = [1.40, 3.36], *p*FDR = .002).

There were no significant changes in drinking behaviours from T1-T3 (see S1 Table 6 in S1 File).

### Motivations for alcohol use

Similar to the findings for alcohol use behaviour, participants had significantly lower odds at T1 of reporting increased drinking to kill time (odds = 0.23, 95% CI = [0.17, 0.31], *p*FDR < .001), destress (odds = 0.38, 95% CI = [0.29, 0.49], *p*FDR < .001), or to enjoy (odds = 0.46, 95% CI = [0.36, 0.59], *p*FDR < .001) than before the pandemic. These findings indicate that people generally were *not* more likely to endorse these motivations for drinking than before the pandemic. As with the findings of behavioural change, these results do not reflect that people endorsed these motivations less than before the pandemic. Older age was associated with lower odds of reporting increased drinking for these reasons (Table 4), while no effect of country was observed.

**Table 4:** Results for changes in motivation for alcohol use during the pandemic.

Variable	Odds	95% CI	SE	*t*	*p*	*p*FDR
To kill time or boredom						
Pre-pandemic to T1						
**Age**	**0.73**	**[0.63, 0.84]**	**0.08**	**−4.18**	**<.001**	**<.001**
United Kingdom	1.25	[0.88, 1.75]	0.18	1.21	.227	.248
United States	1.46	[0.97, 2.21]	0.21	1.75	.072	.096
T1 to T3						
Age	0.65	[0.41, 1.03]	0.15	−1.83	.067	.184
T2	0.67	[0.36, 1.25]	0.21	−1.26	.207	.334
T3	0.56	[0.29, 1.07]	0.18	−1.76	.079	.184
United Kingdom	1.39	[0.47, 4.12]	0.77	0.60	.551	.635
United States	1.64	[0.48, 5.58]	1.02	0.79	.428	.535
To destress						
Pre-pandemic to T1						
**Age**	**0.82**	**[0.72, 0.93]**	**0.07**	**−3.06**	**.002**	**.004**
United Kingdom	1.04	[0.76, 1.43]	0.16	0.26	.797	.797
United States	1.47	[1.03, 2.11]	0.18	2.11	.035	.056
T1 to T3						
Age	0.72	[0.49, 1.05]	0.14	−1.72	.086	.184
T2	0.60	[0.34, 1.06]	0.17	−1.76	.078	.184
T3	0.71	[0.41, 1.23]	0.20	−1.22	.223	.334
United Kingdom	1.27	[0.50, 3.26]	0.61	0.50	.618	.662
United States	2.20	[0.75, 6.42]	1.20	1.44	.151	.283
To enjoy						
Pre-pandemic to T1						
**Age**	**0.79**	**[0.70, 0.90]**	**0.06**	**−3.71**	**<.001**	**.001**
United Kingdom	1.28	[0.95, 1.74]	0.15	1.61	.107	.129
United States	1.45	[1.02, 2.06]	0.18	2.08	.037	.056
T1 to T3						
**Age**	**0.67**	**[0.58, 0.78]**	**0.05**	**−5.14**	**<.001**	**<.001**
T2	0.94	[0.70, 1.26]	0.14	−0.43	.666	.666
T3	0.86	[0.63, 1.17]	0.13	−0.96	.336	.458
**United Kingdom**	**1.67**	**[1.15, 2.41]**	**0.31**	**2.72**	**.007**	**.033**
**United States**	**1.97**	**[1.30, 3.00]**	**0.42**	**3.19**	**.001**	**.011**

T1 was treated as the reference category for T2 and T3 effects and Australia was treated as the reference category for country effects. Bolded text indicated significant effects at *p*FDR < .05.

From T1-T3, drinking patterns remained stable, with no significant changes in drinking motivated by boredom, stress relief, or enjoyment. A significant effect of age was observed for motivations related to enjoyment, such that older age was associated with reduced odds of reporting increased drinking for enjoyment (Table 4). Living in the United Kingdom and United States was associated with higher odds of reporting increased drinking for enjoyment compared to Australia (Table 4).

### Alcohol and mental health

Cross-lagged panel models provided no evidence of prospective bidirectional associations between changes in alcohol use frequency and quantity and symptoms of depression or anxiety, nor with general or pandemic-related stress. See S1 Tables 7–12 in S1 File for relevant results. Notably, loneliness at T1 and T2 was prospectively associated with alcohol use quantity at subsequent timepoints (i.e., T2 and T3). Cross-sectionally, loneliness was also associated with alcohol use frequency at T2 and T3 (Fig 3).

**Fig 3:**
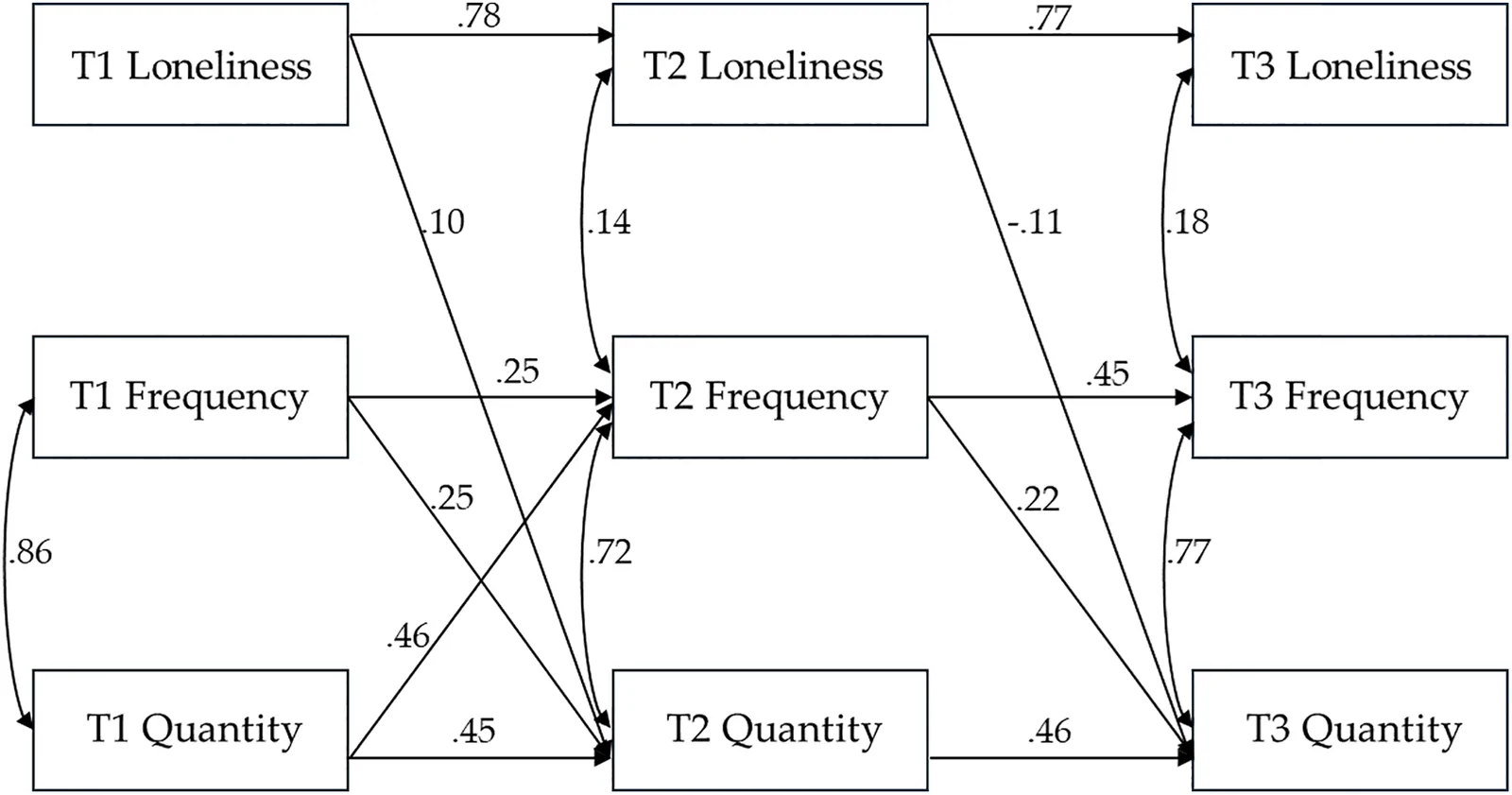
Cross-lagged panel model for associations between loneliness and alcohol consumption across time.

Figure shows standardised coefficients, with only paths significant at *p* < .05 shown. Models covaried for age, country, and pre-pandemic alcohol consumption frequency and quantity. Single arrows indicate time varying regression pathways while double arrows indicate within-timepoint covariances. Model fit: *χ*^2^ (9) = 54.02, *p* < .001, Comparative Fit Index (CFI) = 0.97, Tucker-Lewis Index (TFI) = 0.75, Root Mean Square Error of Approximation (RMSEA) = 0.15, Standardized Root Mean Square Residual (SRMR) = 0.04.

### Sensitivity analysis

Sensitivity analyses showed that our results remained stable after accounting for the effect of COVID-19-related stress and after exclusion of pregnant sample data (see S1 Tables 12–24 in S1 File).

## Discussion

The current study analysed data from the multinational CORAL study to investigate longitudinal changes in alcohol use frequency, quantity, drinking behaviours, and motivations across age, country, and time during the first year of the COVID-19 pandemic (May 2020 – April 2021). We also examined associations of alcohol consumption with mental health, stress, and loneliness. Overall, alcohol use remained relatively stable across the first year of the pandemic. However, higher pre-pandemic quantity predicted greater change following the outbreak, with people in the United Kingdom and United States more likely to report increased consumption than those in Australia. Non-linear age effects were observed- younger and mid-aged individuals were most likely to increase use, whereas older adults tended to reduce use. Alcohol-related behaviours and motivations for alcohol use did not change overall from pre-pandemic levels, although some age- and country-specific differences emerged. Finally, loneliness predicted later alcohol consumption quantity.

Partly consistent with our prediction (H1), increases in alcohol consumption after the COVID-19 outbreak were related to pre-pandemic levels, but only among moderate-to-heavy drinkers who drank 3–4 standard drinks per day. This was not observed among heavy drinkers who drank more than 4–5 drinks per day before the pandemic. This contrasts with prior studies reporting increases in heavy drinkers [20,21], potentially reflecting the low proportion of heavy drinkers in the sample. Further, alcohol use frequency and quantity remained stable over time, diverging from prior research that reported changes in use [9,11,13,45]. The discrepancy may reflect the longitudinal scope of the current study spanning the full first year of the pandemic, while earlier work examined mostly the initial phase of the pandemic. The relative stability we observed may therefore reflect adjustment to initial disruptions or the gradual easing of restrictions over time. In contrast, studies that focused on the acute phase of the pandemic may have captured transient changes that resolved as initial stressors subsided or became normalised [46]. Further, the observed stability may again reflect the predominance of light-to-moderate drinkers in the sample, as prior studies reported increases in primarily heavier drinkers [21,47]. This underscores the importance of considering baseline drinking patterns when investigating the pandemic effects and suggests that prevention and intervention efforts may need to prioritise individuals with a history of heavy alcohol use.

The results showed a non-linear age effect on alcohol use during the pandemic. Specifically, both younger (11−20 years) and mid-aged (50−70 years) individuals were most likely to *increase* their use, whereas adults over 75 were most likely to *reduce* use. These findings may appear inconsistent with our hypothesis (H2c) and prior studies linking older age with increased alcohol use [10]. However, this discrepancy likely stems from differences in age categorisation. For example, one study reported increases among ‘older’ adults was in fact referring those aged 50 and above [48]. Importantly, our findings may reconcile the seemingly mixed results in the literature – increases have been reported among both younger and ‘older’ individuals [10]. Rather than being contradictory, these findings may instead reflect the non-linear age effect observed, with both younger and mid-aged groups particularly susceptible to increases during the pandemic. Further, we also extend prior work by identifying increased alcohol use among underage young people, highlighting the need for targeted prevention (e.g., psychoeducation, parental support) in this vulnerable age group. Conversely, the observed decreases among individuals aged 75 and above may reflect unique challenges faced by older adults during the COVID-19 pandemic, including heightened health concerns, disrupted support services, and reduced access to alcohol due to mobility limitations or limited familiarity with online purchasing platforms [49,50].

We found no changes in overall alcohol use behaviours or motivations from before to during the pandemic, nor across the first year of the pandemic. However, we did find age-related differences: older adults were less likely to begin drinking earlier in the day after the outbreak than before the pandemic. However, this effect did not persist across the pandemic, suggesting it may have been an initial response to the pandemic. Older adults were also less likely to report increased drinking motivated by boredom, stress, or enjoyment after the pandemic outbreak. This may reflect more habitual or ritualistic drinking in this age group, compared to more emotionally driven drinking in younger individuals [51,52]. Although speculative, future research examining detailed drinking related behaviours and motivations could further clarify these age-related differences.

Country-related differences were also observed. Compared to Australia, individuals in the United Kingdom and United States were more likely to increase their drinking during the pandemic. Across the first year, they were also more likely to begin drinking earlier in the day and to drink for enjoyment. These differences may reflect variations in overall alcohol accessibility across countries during the study period (i.e., May 2020 – March 2021). Unlike Australia, both the United Kingdom and United States introduced measures expanding off-premises alcohol access, which came into effect prior to or during the first wave of data collection (i.e., May – September 2020) and continued throughout the study period [15–17]. For example, the United States permitted takeaway, curbside pick-ups, and home delivery of alcohol after March 2020, and the United Kingdom relaxed laws on takeaway sales after July 2020 [15–17]. These changes may have extended alcohol access beyond what was typically available before the pandemic. In contrast, Australia did not introduce comparable measures expanding off-premises alcohol access throughout the study period [53]. Further, on-premises alcohol venues closed during Australia’s initial nationwide lockdown from March to May 2020, which overlapped with T1 data collection. Some states, including Victoria, also experienced more prolonged or recurring localised lockdowns spanning T1 to T2, during which these venues remained closed [54]. Other restrictions, such as nighttime curfews (e.g., Melbourne’s 8pm-5am curfew from August to September 2020) may have further limited opportunities to access alcohol during this period [55]. These combined differences in COVID-19 restrictions and alcohol accessibility policies across countries may therefore be an important contributor to the country-related patterns of alcohol consumption observed in this study. Cultural norms may also contribute to these country-related differences. For example, drinking in Australia has traditionally been associated with mateship and social solidarity [56]. Reduced opportunities for social interactions during the pandemic may have contributed to the absence of increased alcohol use observed in Australia relative to the United States and United Kingdom. Alternatively, or additionally, this may reflect greater distress from higher morbidity and mortality rates in the United States and United Kingdom [57,58]. However, as the effect remained after adjusting for COVID-related stress, country differences may extend beyond COVID-19-induced stress, highlighting the impact of public policy on drinking behaviours.

Finally, although several cross-sectional studies have reported links between increased alcohol use and poor mental health, stress, and loneliness, we found no evidence of such prospective associations over the first year of the pandemic for mental health and stress. Loneliness, however, showed a unidirectional association predicting later alcohol use quantity. Social isolation and restrictions during the COVID-19 pandemic amplified loneliness [59], which has long been implicated as both a contributing and maintaining factor for alcohol misuse [60–62]. The observed associations may therefore partly reflect the use of alcohol as a coping response to loneliness. Indeed, a recent systematic review of 33 studies similarly identified loneliness as one of several psychosocial factors associated with binge drinking among young people during the pandemic [63]. Our longitudinal findings complement this review by showing that loneliness was prospectively associated with subsequent alcohol-use quantity in a sample spanning adolescence to older adulthood. Our findings therefore extend the evidence concerning loneliness to a broader age range and changes in pattern of alcohol use quantity.

The present study has several strengths, including a large multinational sample (albeit all Western countries) and a longitudinal design capturing changes across the pandemic. Several limitations should also be noted. First, generalisability is limited by the use of convenience sampling and a sample primarily composed of Caucasian females. It is known that females generally report lower alcohol use than males, with evidence of the gender gap continuing into the pandemic [10,64]. Consistent with this pattern, the proportion of female participants declined as pre-pandemic drinking quantity increased, from 90% among those reporting 1–2 standard drinks to 33% among those reporting 10 or more standard drinks. This suggests that the predominantly female sample consisted mostly of light-to-moderate drinkers. As past research found that heavy drinkers were more likely to increase alcohol use during the pandemic, the predominantly female composition of our sample likely limited the number of participants at higher risk of increased consumption [20,21]. Relatedly, the low proportion of heavy drinkers in our sample, with fewer than 10% reporting more than 4 standard drinks on a typical day, likely limited the statistical power to detect meaningful effects among heavy drinkers. This may partly explain why increases in consumption were observed only among moderate-to-heavy drinkers, but not among heavy drinkers, and why the overall likelihood of increased alcohol use was low. Beyond consumption patterns, the gender imbalance in our sample may also have implications for the psychological outcomes examined in this study. Evidence suggests that women were disproportionately affected by pandemic-related psychological distress, including anxiety, depression, and loneliness, compared to men [65–67]. The predominantly female composition of our sample may have influenced the observed unidirectional association between loneliness and later alcohol use quantity, and the absence of associations with other mental health outcomes. These findings may therefore not generalise to populations of other genders, who may exhibit different patterns of alcohol use and psychological response to pandemic-related stressors. A second limitation to consider is that alcohol use was measured differently before and during the pandemic. Pre-pandemic use was assessed using absolute indicators of drinking quantity and frequency, whereas alcohol use during the pandemic was assessed as perceived change relative to pre-pandemic levels. This difference may have limited direct comparisons across timepoints and prevented precise estimation of the magnitude of change in alcohol consumption over time. For instance, the absence of statistically significant prospective associations between alcohol use and mental wellbeing outcomes may reflect these measurement limitations, rather than a true lack of associations. Furthermore, self-reported alcohol use is subject to recall bias and social desirability bias. These may have led to under-reporting of changes in drinking quantity, frequency, behaviours, and motivations during the pandemic, potentially affecting the reliability of the findings [68]. Future research could consider methods such as daily drinking diaries or 24-hour recalls to improve accuracy [69].

Nevertheless, findings from the present study have important policy implications. First, our study extended the risk of increased alcohol use during the pandemic to younger and potentially underage individuals. This highlights the need to continue to develop targeted psychoeducation and parental support strategies to reduce underage drinking, especially during periods of enforced or self-selected social isolation. Further, online and delivery-based alcohol sales expanded in the United Kingdom and United States during the pandemic, and continued beyond the pandemic [16,17]. Combined with inconsistent age-verification practices across off-premises distribution channels [70], this highlights the need for stronger verification policies, jointly enforced by policymakers and distributors, to mitigate the risk of underage access. Moreover, the observed country-related differences suggest that alcohol accessibility policy may play a meaningful role in shaping population-level drinking patterns during public health crises. Specifically, the expansion of off-premises alcohol access (e.g., delivery and takeaway sales) in the United Kingdom and United States, relative to the comparatively more restricted access in Australia, was associated with a greater likelihood of increased consumption. This suggests that policymakers should carefully weigh the economic benefits of relaxing alcohol accessibility regulations, such as supporting the hospitality industry, against the potential public health risks of increased consumption, particularly during periods of heightened psychological distress [16,17]. Finally, the prospective association between loneliness and later alcohol use quantity underscores the importance of addressing social isolation as a public health priority [71,72]. Continued efforts in developing interventions aimed at maintaining social connection, such as social prescribing [73], may be important in mitigating the risk of alcohol misuse as a coping response to loneliness in future public health crises.

This study contributes to understanding alcohol use during state-enforced mobility and alcohol sales restrictions. Findings of non-linear age effect and country-related differences in changes in alcohol use, behaviours, and motivations offer a nuanced view of how these patterns vary by individual and context. The observed impact of loneliness on later alcohol consumption also adds to the growing body of evidence that highlights loneliness as a public health issue.

## Supporting information

S1 File Supplementary Materials. This file contains details of the analytical models used in the present study, the data distributions, the full analysis results, and sensitivity analyses results.
(DOCX)

## References

[1] WHO. Timeline: WHO’s COVID-19 response. Available from: https://www.who.int/emergencies/diseases/novel-coronavirus-2019/interactive-timeline. Accessed 2025 July 17.

[2] Wilder-Smith A, Freedman DO. Isolation, quarantine, social distancing and community containment: pivotal role for old-style public health measures in the novel coronavirus (2019-nCoV) outbreak. J Travel Med. 2020;27(2):taaa020. doi: 10.1093/jtm/taaa020 32052841 PMC7107565

[3] CDC. CDC museum COVID-19 timeline. Centers for Disease Control and Prevention. Available from: https://www.cdc.gov/museum/timeline/covid19.html. 2024. Accessed 2025 July 17.

[4] Violant-Holz V, Gallego-Jiménez MG, González-González CS, Muñoz-Violant S, Rodríguez MJ, Sansano-Nadal O, et al. Psychological health and physical activity levels during the COVID-19 pandemic: a systematic review. Int J Environ Res Public Health. 2020;17(24):9419. doi: 10.3390/ijerph17249419 33334073 PMC7765528

[5] Murthy P, Narasimha VL. Effects of the COVID-19 pandemic and lockdown on alcohol use disorders and complications. Curr Opin Psychiatry. 2021;34(4):376–85. doi: 10.1097/YCO.0000000000000720 34016817 PMC8183243

[6] Horta Esper L, Furtado EF. Gender differences and association between psychological stress and alcohol consumption: a systematic review. J Alcohol Drug Depend. 2013. doi: 10.4172/2329-6488.1000116

[7] Koob GF, Powell P, White A. Addiction as a coping response: hyperkatifeia, deaths of despair, and COVID-19. Am J Psychiatry. 2020;177(11):1031–7. doi: 10.1176/appi.ajp.2020.20091375 33135468

[8] Finlay I, Gilmore I. Covid-19 and alcohol-a dangerous cocktail. BMJ. 2020;369:m1987. doi: 10.1136/bmj.m1987 32434792

[9] Sohi I, Chrystoja BR, Rehm J, Wells S, Monteiro M, Ali S, et al. Changes in alcohol use during the COVID-19 pandemic and previous pandemics: a systematic review. Alcohol Clin Exp Res. 2022;46(4):498–513. doi: 10.1111/acer.14792 35412673 PMC9111333

[10] Roberts A, Rogers J, Mason R, Siriwardena AN, Hogue T, Whitley GA, et al. Alcohol and other substance use during the COVID-19 pandemic: a systematic review. Drug Alcohol Depend. 2021;229(Pt A):109150. doi: 10.1016/j.drugalcdep.2021.109150 34749198 PMC8559994

[11] Bantounou MA. A narrative review of the use of alcohol during the Covid-19 pandemic; effects and implications. J Addict Dis. 2023;41(1):30–40. doi: 10.1080/10550887.2022.2058852 35373718

[12] Clare PJ, Aiken A, Yuen WS, Upton E, Kypri K, Degenhardt L, et al. Alcohol use among young Australian adults in May-June 2020 during the COVID-19 pandemic: a prospective cohort study. Addiction. 2021;116(12):3398–407. doi: 10.1111/add.15599 34105838 PMC8212116

[13] Pollard MS, Tucker JS, Green HD Jr. Changes in adult alcohol use and consequences during the COVID-19 pandemic in the US. JAMA Netw Open. 2020;3(9):e2022942. doi: 10.1001/jamanetworkopen.2020.22942 32990735 PMC7525354

[14] Jacob L, Smith L, Armstrong NC, Yakkundi A, Barnett Y, Butler L, et al. Alcohol use and mental health during COVID-19 lockdown: a cross-sectional study in a sample of UK adults. Drug Alcohol Depend. 2021;219:108488. doi: 10.1016/j.drugalcdep.2020.108488 33383352 PMC7768217

[15] Barbosa C, Cowell AJ, Dowd WN. Alcohol consumption in response to the COVID-19 pandemic in the United States. J Addict Med. 2021;15(4):341–4. doi: 10.1097/ADM.0000000000000767 33105169 PMC8327759

[16] Fitzgerald N, Manca F, Uny I, Martin JG, O’Donnell R, Ford A, et al. Lockdown and licensed premises: COVID-19 lessons for alcohol policy. Drug Alcohol Rev. 2022;41(3):533–45. doi: 10.1111/dar.13413 34904313 PMC9300075

[17] Lemp JM, Kilian C, Probst C. Here to stay? Policy changes in alcohol home delivery and “to-go” sales during and after COVID-19 in the United States. Drug Alcohol Rev. 2024;43(2):434–9. doi: 10.1111/dar.13789 38038182 PMC10922279

[18] World Health Organization. Global status report on alcohol and health 2018. Geneva: World Health Organization; 2019.

[19] Fernández-León P, Martínez-Montilla JM, Fagundo-Rivera J, Lima-Serrano M. Deconstructing adolescent binge drinking: a concept analysis with implications for nursing practice. Int J Addict Nurs. 2026. doi: 10.1097/jan.0000000000000689

[20] Celidoni M, Costa-Font J, Salmasi L. Mobility restrictions and alcohol use during lockdown: “A still and dry pandemic for the many”? Econ Hum Biol. 2023;50:101268. doi: 10.1016/j.ehb.2023.101268 37517160

[21] Kilian C, O’Donnell A, Potapova N, López-Pelayo H, Schulte B, Miquel L, et al. Changes in alcohol use during the COVID-19 pandemic in Europe: a meta-analysis of observational studies. Drug Alcohol Rev. 2022;41(4):918–31. doi: 10.1111/dar.13446 35187739 PMC9111882

[22] Choi Y-J, Myung S-K, Lee J-H. Light alcohol drinking and risk of cancer: a meta-analysis of cohort studies. Cancer Res Treat. 2018;50(2):474–87. doi: 10.4143/crt.2017.094 28546524 PMC5912140

[23] Abel EL, Kruger ML, Friedl J. How do physicians define “light,” “moderate,” and “heavy” drinking? Alcohol Clin Exp Res. 1998;22(5):979–84. doi: 10.1111/j.1530-0277.1998.tb03692.x 9726266

[24] Esposito S, Campana BR, Argentiero A, Masetti M, Fainardi V, Principi N. Too young to pour: the global crisis of underage alcohol use. Front Public Health. 2025;13:1598175. doi: 10.3389/fpubh.2025.1598175 40692883 PMC12277329

[25] Harding FM, Hingson RW, Klitzner M, Mosher JF, Brown J, Vincent RM, et al. Underage drinking: a review of trends and prevention strategies. Am J Prev Med. 2016;51(4 Suppl 2):S148-57. doi: 10.1016/j.amepre.2016.05.020 27476384

[26] Jackson KM, Merrill JE, Stevens AK, Hayes KL, White HR. Changes in alcohol use and drinking context due to the COVID-19 pandemic: a multimethod study of college student drinkers. Alcohol Clin Exp Res. 2021;45(4):752–64. doi: 10.1111/acer.14574 33755224 PMC8076077

[27] Biddle N, Edwards B, Gray M, Sollis K. Alcohol consumption during the COVID-19 period: May 2020. The ANU Centre for Social Research and Methods; 2020.

[28] Lechner WV, Laurene KR, Patel S, Anderson M, Grega C, Kenne DR. Changes in alcohol use as a function of psychological distress and social support following COVID-19 related University closings. Addict Behav. 2020;110:106527. doi: 10.1016/j.addbeh.2020.106527 32679435 PMC7319610

[29] Killgore WDS, Cloonan SA, Taylor EC, Vanuk JR, Dailey NS. Morning drinking during COVID-19 lockdowns. Psychiatry Res. 2022;307:114320. doi: 10.1016/j.psychres.2021.114320 34910965 PMC8683136

[30] Rehm J, Kilian C, Ferreira-Borges C, Jernigan D, Monteiro M, Parry CDH, et al. Alcohol use in times of the COVID 19: Implications for monitoring and policy. Drug Alcohol Rev. 2020;39(4):301–4. doi: 10.1111/dar.13074 32358884 PMC7267161

[31] Bragard E, Giorgi S, Juneau P, Curtis BL. Daily diary study of loneliness, alcohol, and drug use during the COVID-19 Pandemic. Alcohol Clin Exp Res. 2022;46(8):1539–51. doi: 10.1111/acer.14889 36018325 PMC9429816

[32] Tsai J, Elbogen EB, Huang M, North CS, Pietrzak RH. Psychological distress and alcohol use disorder during the COVID-19 era among middle- and low-income U.S. adults. J Affect Disord. 2021;288:41–9. doi: 10.1016/j.jad.2021.03.085 33839557 PMC9754659

[33] Capasso A, Jones AM, Ali SH, Foreman J, Tozan Y, DiClemente RJ. Increased alcohol use during the COVID-19 pandemic: the effect of mental health and age in a cross-sectional sample of social media users in the U.S. Prev Med. 2021;145:106422. doi: 10.1016/j.ypmed.2021.106422 33422577 PMC9063034

[34] Tran TD, Hammarberg K, Kirkman M, Nguyen HTM, Fisher J. Alcohol use and mental health status during the first months of COVID-19 pandemic in Australia. J Affect Disord. 2020;277:810–3. doi: 10.1016/j.jad.2020.09.012 33065821 PMC7476559

[35] Chau AKC, Minihan S, Okayama S, Schweizer S. The relationship between cognitive and affective control and symptoms of depression and anxiety across the lifespan: a 3-wave longitudinal study. Compr Psychiatry. 2025;137:152564. doi: 10.1016/j.comppsych.2024.152564 39647234

[36] Bush K, Kivlahan DR, McDonell MB, Fihn SD, Bradley KA. The AUDIT alcohol consumption questions (AUDIT-C): an effective brief screening test for problem drinking. Arch Intern Med. 1998;158:1789–95. doi: 10.1001/archinte.158.16.1789 9738608

[37] Dolli I, Slade T, Teesson M, Chapman C. Longitudinal and self-attributed change in alcohol use among young adults during the COVID-19 pandemic in Australia. Drug Alcohol Rev. 2023;42(3):625–32. doi: 10.1111/dar.13602 36682030 PMC10947013

[38] Choi KR, Heilemann MV, Fauer A, Mead M. A second pandemic: mental health spillover from the novel coronavirus (COVID-19). J Am Psychiatr Nurses Assoc. 2020;26:340–3. doi: 10.1177/1078390320919803 32340586

[39] Cohen S, Kamarck T, Mermelstein R. A global measure of perceived stress. J Health Soc Behav. 1983;24(4):385–96. doi: 10.2307/2136404 6668417

[40] Russell DW. UCLA Loneliness Scale (Version 3): reliability, validity, and factor structure. J Pers Assess. 1996;66(1):20–40. doi: 10.1207/s15327752jpa6601_2 8576833

[41] Kroenke K, Spitzer RL, Williams JB. The PHQ-9: validity of a brief depression severity measure. J Gen Intern Med. 2001;16(9):606–13. doi: 10.1046/j.1525-1497.2001.016009606.x 11556941 PMC1495268

[42] Spitzer RL, Kroenke K, Williams JBW, Löwe B. A brief measure for assessing generalized anxiety disorder: the GAD-7. Arch Intern Med. 2006;166(10):1092–7. doi: 10.1001/archinte.166.10.1092 16717171

[43] R Core Team. R: a language and environment for statistical computing. Vienna, Austria: R Foundation for Statistical Computing; 2025.

[44] Benjamini Y, Hochberg Y. Controlling the false discovery rate: a practical and powerful approach to multiple testing. J R Stat Soc B: Stat Methodol. 1995;57(1):289–300. doi: 10.1111/j.2517-6161.1995.tb02031.x

[45] Kilian C, Rehm J, Allebeck P, Braddick F, Gual A, Barták M, et al. Alcohol consumption during the COVID-19 pandemic in Europe: a large-scale cross-sectional study in 21 countries. Addiction. 2021;116(12):3369–80. doi: 10.1111/add.15530 34109685

[46] Daly M, Robinson E. Psychological distress and adaptation to the COVID-19 crisis in the United States. J Psychiatr Res. 2021;136:603–9. doi: 10.1016/j.jpsychires.2020.10.035 33138985 PMC7588823

[47] Nagahama S, Dhungel B, Hori A, Michikawa T, Asakura K, Nishiwaki Y. Changes in the frequency and amount of alcohol intake before and during the COVID-19 pandemic. J Occup Health. 2024;66(1):uiae055. doi: 10.1093/joccuh/uiae055 39397380 PMC11561262

[48] Knell G, Robertson MC, Dooley EE, Burford K, Mendez KS. Health behavior changes during COVID-19 pandemic and subsequent “Stay-at-Home” orders. Int J Environ Res Public Health. 2020;17(17):6268. doi: 10.3390/ijerph17176268 32872179 PMC7504386

[49] Gilbert AS, Garratt SM, Brijnath B, Ostaszkiewicz J, Batchelor F, Dang C, et al. “Keeping our distance”: Older adults’ experiences during year one of the COVID-19 pandemic and lockdown in Australia. J Aging Stud. 2023;67:101170. doi: 10.1016/j.jaging.2023.101170 38012941

[50] Sallie SN, Ritou V, Bowden-Jones H, Voon V. Assessing international alcohol consumption patterns during isolation from the COVID-19 pandemic using an online survey: highlighting negative emotionality mechanisms. BMJ Open. 2020;10(11):e044276. doi: 10.1136/bmjopen-2020-044276 33243820 PMC7692002

[51] Bareham BK, Kaner E, Spencer LP, Hanratty B. Drinking in later life: a systematic review and thematic synthesis of qualitative studies exploring older people’s perceptions and experiences. Age Ageing. 2019;48(1):134–46. doi: 10.1093/ageing/afy069 29733341 PMC6322501

[52] Kuntsche E, Knibbe R, Gmel G, Engels R. Why do young people drink? A review of drinking motives. Clin Psychol Rev. 2005;25(7):841–61. doi: 10.1016/j.cpr.2005.06.002 16095785

[53] Miller M, Mojica-Perez Y, Livingston M. A timeline of alcohol-relevant restrictions during the COVID-19 pandemic. 2023.

[54] Colbert S, Wilkinson C, Thornton L, Richmond R. COVID-19 and alcohol in Australia: Industry changes and public health impacts. Drug Alcohol Rev. 2020;39(5):435–40. doi: 10.1111/dar.13092 32483882 PMC7300689

[55] Victorian Equal Opportunity and Human Rights Commission. 2020 report on the operation of the Charter of Human Rights and Responsibilities: Victorian COVID restrictions – a timeline. Melbourne, Australia: Victorian Equal Opportunity and Human Rights Commission; 2020. Available from: https://www.humanrights.vic.gov.au/static/aa2d1e2e64aa07240a504ddb0ee130d9/Resource-Charter_report-2020-Victorian_COVID_restrictions-a_timeline.pdf

[56] Savic M, Room R, Mugavin J, Pennay A, Livingston M. Defining “drinking culture”: a critical review of its meaning and connotation in social research on alcohol problems. Drugs: Educ Prev Policy. 2016;23(4):270–82. doi: 10.3109/09687637.2016.1153602

[57] Msemburi W, Karlinsky A, Knutson V, Aleshin-Guendel S, Chatterji S, Wakefield J. The WHO estimates of excess mortality associated with the COVID-19 pandemic. Nature. 2023;613(7942):130–7. doi: 10.1038/s41586-022-05522-2 36517599 PMC9812776

[58] Karlinsky A, Kobak D. Tracking excess mortality across countries during the COVID-19 pandemic with the World Mortality Dataset. Elife. 2021;10:e69336. doi: 10.7554/eLife.69336 34190045 PMC8331176

[59] Killgore WDS, Cloonan SA, Taylor EC, Dailey NS. Loneliness: a signature mental health concern in the era of COVID-19. Psychiatry Res. 2020;290:113117. doi: 10.1016/j.psychres.2020.113117 32480121 PMC7255345

[60] Walsh BE, Schlauch RC. Differential impact of emotional and social loneliness on daily alcohol consumption in individuals with alcohol use disorder. Drug Alcohol Depend. 2024;264:112433. doi: 10.1016/j.drugalcdep.2024.112433 39265209

[61] Gutkind S, Gorfinkel LR, Hasin DS. Prospective effects of loneliness on frequency of alcohol and marijuana use. Addict Behav. 2022;124:107115. doi: 10.1016/j.addbeh.2021.107115 34543868 PMC8511227

[62] Akerlind I, Hörnquist JO. Loneliness and alcohol abuse: a review of evidences of an interplay. Soc Sci Med. 1992;34(4):405–14. doi: 10.1016/0277-9536(92)90300-f 1566121

[63] Merino-Casquero A, Andrade-Gómez E, Fagundo-Rivera J, Fernández-León P. Beyond confinement: a systematic review on factors influencing binge drinking among adolescents and young adults during the pandemic. J Clin Med. 2025;14(5):1546. doi: 10.3390/jcm14051546 40095494 PMC11900273

[64] White AM. Gender differences in the epidemiology of alcohol use and related harms in the United States. Alcohol Res. 2020;40(2):01. doi: 10.35946/arcr.v40.2.01 33133878 PMC7590834

[65] Ausín B, González-Sanguino C, Castellanos MÁ, Muñoz M. Gender-related differences in the psychological impact of confinement as a consequence of COVID-19 in Spain. J Gend Stud. 2020;30(1):29–38. doi: 10.1080/09589236.2020.1799768

[66] Kolakowsky-Hayner SA, Goldin Y, Kingsley K, Alzueta E, Arango-Lasprilla JC, Perrin PB. Psychosocial impacts of the COVID-19 quarantine: a study of gender differences in 59 countries. Medicina. 2021;57:789. doi: 10.3390/medicina57080789 PMC8400641 34440995

[67] Prowse R, Sherratt F, Abizaid A, Gabrys RL, Hellemans KGC, Patterson ZR, et al. Coping With the COVID-19 pandemic: examining gender differences in stress and mental health among university students. Front Psychiatry. 2021;12:650759. doi: 10.3389/fpsyt.2021.650759 33897499 PMC8058407

[68] Stockwell T, Zhao J, Panwar S, Roemer A, Naimi T, Chikritzhs T. Do “Moderate” drinkers have reduced mortality risk? A systematic review and meta-analysis of alcohol consumption and all-cause mortality. J Stud Alcohol Drugs. 2016;77(2):185–98. doi: 10.15288/jsad.2016.77.185 26997174 PMC4803651

[69] Patterson C, Hogan L, Cox M. A comparison between two retrospective alcohol consumption measures and the daily drinking diary method with university students. Am J Drug Alcohol Abuse. 2019;45(3):248–53. doi: 10.1080/00952990.2018.1514617 30230918

[70] Duthie C, Pocock T, Curl A, Clark E, Norriss D, Bidwell S, et al. Online on-demand delivery services of food and alcohol: a scoping review of public health impacts. SSM Popul Health. 2023;21:101349. doi: 10.1016/j.ssmph.2023.101349 36845670 PMC9950721

[71] Holt-Lunstad J, Sine K. Loneliness and social connection in the mental health crisis. Biol Psychiatry. 2025;97:939–41. doi: 10.1016/j.biopsych.2024.10.019 39481777

[72] WHO. WHO commission on social connection. Available from: https://www.who.int/groups/commission-on-social-connection. Accessed 2026 July 30.

[73] Mitchell SB, Cartwright L, Gude A, Bethel A, Hunt H, Smith JR, et al. The use of social prescribing and community-based wellbeing activities as a potential prevention and early intervention pathway to improve adolescent emotional and social development: a systematic mapping review. BMC Public Health. 2025;25(1):3495. doi: 10.1186/s12889-025-24413-5 41094654 PMC12522731

