# Supplementary material for: Alcohol use and its associations with mental health across the first year of the COVID-19 pandemic
Source: PLoS One. 2026 Oct 30;21(10):e0361854. doi: 10.1371/journal.pone.0361854 (PMC13722157; doi:10.1371/journal.pone.0361854)
Supplement: Supplementary file 1 [file pone.0361854.s001.docx]

# Supplementary Materials

Table of Contents

[Analysis Model Equations	2](#_Toc218773816)

[Data Distribution	5](#_Toc218773817)

[Main Analysis Results	6](#_Toc218773818)

[Sensitivity Analysis Results	18](#_Toc218773819)

[Results accounting for pandemic-related stress	18](#_Toc218773820)

[Results for analyses without pregnant sample	22](#_Toc218773821)

## Analysis Model Equations

H1 - Associations between pre-pandemic drinking and drinking after the start of COVID at T1:

*Drinking quantity/frequency at T1 (factor) ~ pre-pandemic drinking quantity/frequency at T1 (factor) + country (factor) + pandemic anxiety scale (PAS) total score at T1 (continuous)^^*

H2a - Changes in drinking quantity and frequency during the pandemic (T1-T3):

*Drinking quantity/frequency^ ~ (age + age^2^ + age^3^) * assessment timepoint + country + PAS total score^^ + (1 | participant ID)*

H2b - Changes in drinking behaviours from before the pandemic to T1 and during the pandemic (T1-T3):

Pre-pandemic to T1:

*Changes in drinking behaviours from pre-pandemic at T1 ~ age + country + PAS total score^^*

T1 - T3:

*Changes in drinking behaviours from pre-pandemic^ ~ age * assessment timepoint + country + PAS total score ^^ + (1 | participant ID)*

H3 - Changes in motivations for drinking from before the pandemic to T1 and across the pandemic (T1-T3):

Pre-pandemic to T1:

*Changes in motivations for drinking from pre-pandemic at T1 ~ age + country + PAS total score^^*

T1 – T3:

*Changes in motivations for drinking from pre-pandemic^ ~ age * assessment timepoint + country + PAS total score ^^ + (1 | participant ID)*

Model notations explained:

^ denotes time varying variables

^^ denotes variables included only in sensitivity analyses

1 | participant ID represents random effect of participant ID

H4:

See Figure 1 for cross-lagged panel model diagrams that assessed the bi-directional associations of changes in drinking quantity and frequency with mental health and stress

**Figure S1**

*Cross-Lagged Panel Model Diagram for H4 Analysis*

*Note*. Single headed arrows represent cross-lagged paths, double headed arrows represent covariances, and circles represent error variances. All models covaried for age at T1, pre-pandemic drinking frequency and quantity, and country. MH abbreviates for mental health, which includes variables of depressive symptoms, anxiety symptoms, loneliness, COVID-19 related stress, and general stress (modelled separately).

## Data Distribution

**Figure S2**

*Histograms of continuous variables*

**Table S1**

Distribution of alcohol use quantity and frequency by country

| **Variable** | **Australia** | **UK** | **US** |
| --- | --- | --- | --- |
| **Indicated alcohol use** | | | |
| *T1* |  |  |  |
| Yes | 297 (50%) | 494 (46%) | 420 (60%) |
| No | 297 (50%) | 579 (54%) | 282 (40%) |
| *T2* |  |  |  |
| Yes | 105 (33%) | 121 (36%) | 146 (52%) |
| No | 216 (67%) | 216 (64%) | 134 (48%) |
| *T3* |  |  |  |
| Yes | 66 (36%) | 108 (34%) | 135 (49%) |
| No | 118 (64%) | 206 (66%) | 142 (51%) |
| **Pre-pandemic alcohol use quantity (standard drink)** | | | |
| 1-2 | 226 (79%) | 417 (74%) | 247 (89%) |
| 3-4 | 45 (16%) | 94 (17%) | 25 (9%) |
| 5-6 | 10 (3%) | 39 (7%) | 3 (1%) |
| 7-9 | 4 (1%) | 11 (2%) | 1 (0%) |
| 10 or more | 2 (1%) | 4 (1%) | 0 (0%) |
| **Change in alcohol use quantity relative to pre-pandemic levels** | | | |
| *T1* |  |  |  |
| A lot less | 76 (26%) | 117 (20%) | 44 (16%) |
| A bit less | 31 (11%) | 56 (10%) | 26 (9%) |
| About the same | 126 (43%) | 239 (41%) | 135 (48%) |
| A bit more | 49 (17%) | 126 (22%) | 62 (22%) |
| A lot more | 11 (4%) | 39 (7%) | 14 (5%) |
| *T2* |  |  |  |
| A lot less | 27 (21%) | 31 (14%) | 20 (15%) |
| A bit less | 17 (13%) | 35 (16%) | 16 (12%) |
| About the same | 52 (41%) | 98 (45%) | 64 (48%) |
| A bit more | 23 (18%) | 43 (20%) | 25 (19%) |
| A lot more | 7 (6%) | 9 (4%) | 9 (7%) |
| *T3* |  |  |  |
| A lot less | 24 (21%) | 34 (17%) | 25 (18%) |
| A bit less | 17 (15%) | 27 (13%) | 19 (13%) |
| About the same | 57 (49%) | 92 (45%) | 64 (45%) |
| A bit more | 16 (14%) | 38 (19%) | 26 (18%) |
| A lot more | 3 (3%) | 13 (6%) | 7 (5%) |
| **Pre-pandemic alcohol use frequency** | | | |
| Never | 10 (3%) | 7 (1%) | 4 (1%) |
| Monthly or less | 65 (22%) | 162 (28%) | 93 (33%) |
| 2-4 times per month | 89 (30%) | 197 (34%) | 87 (31%) |
| 2-3 times per week | 83 (28%) | 148 (26%) | 54 (19%) |
| More than 4 times a week | 47 (16%) | 64 (11%) | 43 (15%) |
| **Change in alcohol use frequency relative to pre-pandemic levels** | | | |
| *T1* |  |  |  |
| A lot less | 71 (24%) | 113 (20%) | 43 (15%) |
| A bit less | 35 (12%) | 46 (8%) | 24 (9%) |
| About the same | 106 (36%) | 193 (33%) | 104 (37%) |
| A bit more | 65 (22%) | 160 (28%) | 94 (33%) |
| A lot more | 16 (5%) | 65 (11%) | 16 (6%) |
| *T2* |  |  |  |
| A lot less | 25 (20%) | 25 (12%) | 23 (17%) |
| A bit less | 18 (14%) | 32 (15%) | 15 (11%) |
| About the same | 48 (38%) | 95 (44%) | 53 (40%) |
| A bit more | 24 (19%) | 49 (23%) | 31 (23%) |
| A lot more | 11 (9%) | 15 (7%) | 12 (9%) |
| *T3* |  |  |  |
| A lot less | 21 (18%) | 38 (19%) | 23 (16%) |
| A bit less | 19 (16%) | 28 (14%) | 19 (13%) |
| About the same | 54 (46%) | 76 (37%) | 57 (40%) |
| A bit more | 18 (15%) | 46 (23%) | 33 (23%) |
| A lot more | 5 (4%) | 16 (8%) | 9 (6%) |

*Note.* Percentages reflect % of the total sample within each country (minor deviations due to rounding).

**Table S2**

Distribution of alcohol use quantity and frequency before the COVID-19 pandemic by gender

| **Variable** | **Female** | **Male** | **Other/NA** |
| --- | --- | --- | --- |
| **Indicated alcohol use** | | | |
| *T1* |  |  |  |
| Yes | 1,018 (48%) | 129 (61%) | 13 (45%) |
| No | 1,112 (52%) | 83 (39%) | 16 (55%) |
| *T2* |  |  |  |
| Yes | 327 (45%) | 33 (34%) | 13 (65%) |
| No | 404 (55%) | 65 (66%) | 7 (35%) |
| *T3* |  |  |  |
| Yes | 265 (40%) | 29 (34%) | 16 (89%) |
| No | 405 (60%) | 57 (66%) | 2 (11%) |
| **Pre-pandemic alcohol use quantity (standard drink)** | | | |
| 1-2 | 798 (81%) | 83 (66%) | 9 (82%) |
| 3-4 | 138 (14%) | 25 (20%) | 1 (9%) |
| 5-6 | 44 (4%) | 7 (6%) | 1 (9%) |
| 7-9 | 9 (1%) | 7 (6%) | 0 (0%) |
| 10 or more | 2 (0%) | 4 (3%) | 0 (0%) |
| **Change in alcohol use quantity relative to pre-pandemic levels** | | | |
| *T1* |  |  |  |
| A lot less | 213 (21%) | 24 (19%) | 0 (0%) |
| A bit less | 96 (9%) | 16 (12%) | 1 (9%) |
| About the same | 437 (43%) | 55 (43%) | 8 (73%) |
| A bit more | 211 (21%) | 25 (20%) | 1 (9%) |
| A lot more | 55 (5%) | 8 (6%) | 1 (9%) |
| *T2* |  |  |  |
| A lot less | 67 (17%) | 10 (15%) | 1 (14%) |
| A bit less | 56 (14%) | 12 (18%) | 0 (0%) |
| About the same | 182 (45%) | 28 (43%) | 4 (57%) |
| A bit more | 78 (19%) | 11 (17%) | 2 (29%) |
| A lot more | 21 (5%) | 4 (6%) | 0 (0%) |
| *T3* |  |  |  |
| A lot less | 74 (18%) | 9 (16%) | 0 (0%) |
| A bit less | 52 (13%) | 11 (19%) | 0 (0%) |
| About the same | 187 (47%) | 24 (42%) | 2 (50%) |
| A bit more | 69 (17%) | 9 (16%) | 2 (50%) |
| A lot more | 19 (5%) | 4 (7%) | 0 (0%) |
| **Pre-pandemic alcohol use frequency** | | | |
| Never | 20 (2%) | 1 (1%) | 0 (0%) |
| Monthly or less | 294 (29%) | 20 (16%) | 6 (55%) |
| 2-4 times per month | 328 (32%) | 41 (32%) | 4 (36%) |
| 2-3 times per week | 248 (24%) | 37 (29%) | 0 (0%) |
| More than 4 times a week | 124 (12%) | 29 (23%) | 1 (9%) |
| **Change in alcohol use frequency relative to pre-pandemic levels** | | | |
| *T1* |  |  |  |
| A lot less | 206 (20%) | 21 (16%) | 0 (0%) |
| A bit less | 85 (8%) | 20 (16%) | 0 (0%) |
| About the same | 350 (35%) | 46 (36%) | 7 (64%) |
| A bit more | 282 (28%) | 33 (26%) | 4 (36%) |
| A lot more | 89 (9%) | 8 (6%) | 0 (0%) |
| *T2* |  |  |  |
| A lot less | 62 (15%) | 10 (15%) | 1 (14%) |
| A bit less | 50 (12%) | 15 (23%) | 0 (0%) |
| About the same | 169 (42%) | 23 (35%) | 4 (57%) |
| A bit more | 91 (23%) | 13 (20%) | 0 (0%) |
| A lot more | 32 (8%) | 4 (6%) | 2 (29%) |
| *T3* |  |  |  |
| A lot less | 74 (18%) | 8 (14%) | 0 (0%) |
| A bit less | 55 (14%) | 11 (19%) | 0 (0%) |
| About the same | 160 (40%) | 24 (42%) | 3 (75%) |
| A bit more | 86 (21%) | 10 (18%) | 1 (25%) |
| A lot more | 26 (6%) | 4 (7%) | 0 (0%) |

*Note.* Percentages are calculated within each gender and wave using non-missing data only.

## Main Analysis Supplementary Results

**Table S3**

*Model comparison results for linear, quadratic, and cubic age models for H2*

| Outcome | Model | df | AIC | BIC | logLik |
| --- | --- | --- | --- | --- | --- |
| Quantity Model | Cubic age | 39 | 5502.15 | 5727.93 | -2711.07 |
|  | Quadratic age | 30 | 5510.70 | 5685.68 | -2724.35 |
|  | Linear age | 21 | 5511.35 | 5635.53 | -2733.68 |
| Frequency Model | Cubic age | 13 | 5831.71 | 5910.73 | -2901.85 |
|  | Quadratic age | 11 | 5841.46 | 5894.81 | -2909.18 |
|  | Linear age | 9 | 5838.36 | 5909.19 | -2908.73 |

*Note.* CLMM = cumulative link mixed model; df = degrees of freedom; AIC = Akaike Information Criterion; BIC = Bayesian Information Criterion; logLik = log-likelihood. Lower AIC/BIC values and higher (less negative) logLik values indicate better model fit. For both quantity and frequency, the cubic age model provided the best fit based on AIC and log-likelihood.

**Table S4**

*Results for pre-pandemic alcohol consumption frequency and quantity in association with relative change in alcohol consumption at T1*

| Variable | Odds | 95% CI | *t* | *p* |
| --- | --- | --- | --- | --- |
| *Quantity Model* |  |  |  |  |
| Pre-pandemic quantity level |  |  |  |  |
| 3-4 vs 1-2 std drinks | 1.40 | [1.01, 1.92] | 2.05 | .**041** |
| 5-6 vs 1-2 std drinks | 1.45 | [0.83, 2.52] | 1.31 | .190 |
| 7-9 vs 1-2 std drinks | 1.04 | [0.4, 2.69] | 0.08 | .935 |
| 10 or more vs 1-2 std drinks | 2.15 | [0.43, 10.98] | 0.94 | .349 |
| Country |  |  |  |  |
| U.K. vs Australia | 1.45 | [1.12, 1.89] | 2.79 | **.005** |
| U.S. vs Australia | 1.70 | [1.26, 2.3] | 3.44 | **.001** |
| *Frequency Model* |  |  |  |  |
| Pre-pandemic frequency level |  |  |  |  |
| Monthly or less vs never | 0.70 | [0.3, 1.6] | -0.86 | .389 |
| 2-4 times/month vs never | 0.67 | [0.29, 1.54] | -0.94 | .346 |
| 2-3 times/week vs never | 1.01 | [0.44, 2.32] | 0.02 | .988 |
| 4+ times/week vs never | 1.08 | [0.46, 2.54] | 0.18 | .858 |
| Country |  |  |  |  |
| U.K. vs Australia | 1.67 | [1.29, 2.15] | 3.91 | **<.001** |
| U.S. vs Australia | 1.72 | [1.28, 2.31] | 3.62 | **<.001** |

*Note.* Std = standard.

**Table S5**

*Results for associations between age, timepoint, country, and relative change in alcohol consumption frequency and quantity from pre-pandemic levels analysis*

| Variable | Odds | 95% CI | *t* | *p* |
| --- | --- | --- | --- | --- |
| Full model results (with time x age interaction) |  |  |  |  |
| *Quantity model* |  |  |  |  |
| Age | 2.75 | [1.85, 4.08] | 5.01 | **<.001** |
| Age^2^ | 1.52 | [1.12, 2.07] | 2.66 | **.008** |
| Age^3^ | 0.68 | [0.56, 0.83] | -3.92 | **<.001** |
| T2 (vs T1) | 0.95 | [0.65, 1.39] | -0.27 | .784 |
| T3 (vs T1) | 0.79 | [0.53, 1.16] | -1.19 | .232 |
| U.K. (vs Australia) | 1.99 | [1.29, 3.06] | 3.13 | **.002** |
| U.S. (vs Australia) | 2.05 | [1.26, 3.35] | 2.89 | **.004** |
| Age x T2 | 0.80 | [0.49, 1.3] | -0.90 | .366 |
| Age x T3 | 0.75 | [0.46, 1.21] | -1.19 | .232 |
| Age^2^ x T2 | 1.32 | [0.85, 2.03] | 1.25 | .213 |
| Age^2^ x T3 | 1.10 | [0.71, 1.7] | 0.44 | .662 |
| Age^3^ x T2 | 0.91 | [0.71, 1.16] | -0.77 | .438 |
| Age^3^ x T3 | 1.02 | [0.81, 1.28] | 0.16 | .870 |
| *Frequency model* |  |  |  |  |
| Age | 1.96 | [1.37, 2.81] | 3.65 | **<.001** |
| Age^2^ | 1.20 | [0.90, 1.60] | 1.24 | .216 |
| Age^3^ | 0.79 | [0.66, 0.94] | -2.65 | **.008** |
| T2 (vs T1) | 0.87 | [0.61, 1.26] | -0.72 | .473 |
| T3 (vs T1) | 0.65 | [0.45, 0.95] | -2.24 | **.025** |
| U.K. (vs Australia) | 2.10 | [1.41, 3.13] | 3.66 | **<.001** |
| U.S. (vs Australia) | 2.01 | [1.29, 3.15] | 3.06 | **.002** |
| Age x T2 | 0.92 | [0.58, 1.47] | -0.33 | .740 |
| Age x T3 | 1.00 | [0.64, 1.57] | 0.01 | .994 |
| Age^2^ x T2 | 1.29 | [0.85, 1.97] | 1.20 | .230 |
| Age^2^ x T3 | 1.24 | [0.82, 1.88] | 1.04 | .299 |
| Age^3^ x T2 | 0.89 | [0.71, 1.11] | -1.03 | .305 |
| Age^3^ x T3 | 0.91 | [0.74, 1.12] | -0.93 | .354 |

**Table S6**

*Results for associations between age, timepoint, country, and relative change in alcohol use behaviour from pre-pandemic levels analysis*

| Variable | Odds | 95% CI | *t* | *p* | *p*FDR |
| --- | --- | --- | --- | --- | --- |
| Baseline models (pre-COVID to T1) |  |  |  |  |  |
| *Drinking earlier* |  |  |  |  |  |
| (Intercept) | 0.16 | [0.11, 0.22] | -10.66 | <.001 | **<.001** |
| Age | 0.79 | [0.68, 0.91] | -3.13 | .002 | **.004** |
| U.K. (vs Australia) | 1.85 | [1.26, 2.76] | 3.08 | .002 | **.004** |
| U.S. (vs Australia) | 2.15 | [1.4, 3.36] | 3.44 | .001 | **.002** |
| *Difficulty stopping drinking* |  |  |  |  |  |
| (Intercept) | 0.12 | [0.08, 0.17] | -11.13 | <.001 | **<.001** |
| Age | 0.99 | [0.82, 1.2] | -0.08 | .939 | .939 |
| U.K. (vs Australia) | 0.83 | [0.52, 1.33] | -0.80 | .423 | .508 |
| U.S. (vs Australia) | 0.84 | [0.48, 1.44] | -0.64 | .521 | .568 |
| *Drinking alone* |  |  |  |  |  |
| (Intercept) | 0.27 | [0.21, 0.36] | -9.01 | <.001 | **<.001** |
| Age | 0.89 | [0.77, 1.02] | -1.72 | .085 | .146 |
| U.K. (vs Australia) | 1.16 | [0.83, 1.64] | 0.87 | .383 | .508 |
| U.S. (vs Australia) | 1.18 | [0.79, 1.75] | 0.81 | .419 | .508 |
| Prospective models (T1-T3) |  |  |  |  |  |
| *Drinking earlier* |  |  |  |  |  |
| Age | 0.69 | [0.43, 1.11] | -1.52 | .128 | .481 |
| T2 (vs T1) | 0.35 | [0.17, 0.71] | -2.87 | .004 | .062 |
| T3 (vs T1) | 0.40 | [0.19, 0.83] | -2.46 | .014 | .105 |
| U.K. (vs Australia) | 2.10 | [0.61, 7.2] | 1.18 | .239 | .597 |
| U.S. (vs Australia) | 2.59 | [0.66, 10.15] | 1.37 | .172 | .515 |
| *Difficulty stopping drinking* |  |  |  |  |  |
| Age | 0.85 | [0.57, 1.28] | -0.77 | .442 | .828 |
| T2 (vs T1) | 0.63 | [0.36, 1.13] | -1.54 | .123 | .481 |
| T3 (vs T1) | 1.26 | [0.72, 2.19] | 0.80 | .423 | .828 |
| U.K. (vs Australia) | 1.27 | [0.48, 3.39] | 0.48 | .630 | .860 |
| U.S. (vs Australia) | 1.16 | [0.38, 3.56] | 0.26 | .793 | .915 |
| *Drinking alone* |  |  |  |  |  |
| Age | 0.91 | [0.53, 1.57] | -0.34 | .737 | .915 |
| T2 (vs T1) | 1.22 | [0.57, 2.61] | 0.51 | .611 | .860 |
| T3 (vs T1) | 1.28 | [0.58, 2.78] | 0.61 | .540 | .860 |
| U.K. (vs Australia) | 1.03 | [0.27, 3.89] | 0.04 | .965 | .965 |
| U.S. (vs Australia) | 0.94 | [0.2, 4.37] | -0.08 | .937 | .965 |

*Note.* Results based on reduced model without age by wave interaction as full model did not yield significant interaction effects.

**Table S7**

*Results for cross-lagged panel model examining associations between depression and alcohol consumption frequency and quantity*

| Predictor | Outcome | Path type | *b* | 95% CI | *p* |
| --- | --- | --- | --- | --- | --- |
| T1 Depression | T2 Depression | Regression | 0.80 | [0.7, 0.9] | **<.001** |
| T1 Alcohol Frequency | T2 Depression | Regression | -0.05 | [-1.09, 0.98] | .918 |
| T1 Alcohol Quantity | T2 Depression | Regression | 0.33 | [-0.65, 1.31] | .512 |
| T1 Depression | T2 Alcohol Frequency | Regression | 0.00 | [-0.01, 0.02] | .703 |
| T1 Alcohol Frequency | T2 Alcohol Frequency | Regression | 0.32 | [0.12, 0.53] | **.002** |
| T1 Alcohol Quantity | T2 Alcohol Frequency | Regression | 0.43 | [0.23, 0.63] | **<.001** |
| T1 Depression | T2 Alcohol Quantity | Regression | 0.01 | [-0.01, 0.03] | .194 |
| T1 Alcohol Frequency | T2 Alcohol Quantity | Regression | 0.31 | [0.13, 0.49] | **.001** |
| T1 Alcohol Quantity | T2 Alcohol Quantity | Regression | 0.38 | [0.2, 0.56] | **<.001** |
| T2 Depression | T3 Depression | Regression | 0.71 | [0.62, 0.8] | **<.001** |
| T2 Alcohol Frequency | T3 Depression | Regression | -0.21 | [-0.89, 0.48] | .551 |
| T2 Alcohol Quantity | T3 Depression | Regression | 0.34 | [-0.43, 1.12] | .381 |
| T2 Depression | T3 Alcohol Frequency | Regression | -0.01 | [-0.02, 0.01] | .509 |
| T2 Alcohol Frequency | T3 Alcohol Frequency | Regression | 0.43 | [0.21, 0.65] | **<.001** |
| T2 Alcohol Quantity | T3 Alcohol Frequency | Regression | 0.23 | [-0.01, 0.47] | .060 |
| T2 Depression | T3 Alcohol Quantity | Regression | -0.01 | [-0.02, 0.01] | .564 |
| T2 Alcohol Frequency | T3 Alcohol Quantity | Regression | 0.21 | [0.03, 0.39] | **.022** |
| T2 Alcohol Quantity | T3 Alcohol Quantity | Regression | 0.45 | [0.25, 0.65] | **<.001** |
| Age | T1 Depression | Regression | -0.08 | [-0.12, -0.04] | **<.001** |
| Pre-COVID Frequency | T1 Depression | Regression | -0.43 | [-1.16, 0.3] | .247 |
| Pre-COVID Quantity | T1 Depression | Regression | 1.20 | [-0.35, 2.75] | .130 |
| U.K. (vs Australia) | T1 Depression | Regression | 1.56 | [-0.08, 3.21] | .063 |
| U.S. (vs Australia) | T1 Depression | Regression | 1.30 | [-0.53, 3.13] | .164 |
| Age | T1 Alcohol Frequency | Regression | 0.00 | [0, 0.01] | .380 |
| Pre-COVID Frequency | T1 Alcohol Frequency | Regression | 0.04 | [-0.11, 0.18] | .615 |
| Pre-COVID Quantity | T1 Alcohol Frequency | Regression | 0.10 | [-0.07, 0.26] | .252 |
| U.K. (vs Australia) | T1 Alcohol Frequency | Regression | 0.22 | [-0.13, 0.57] | .216 |
| U.S. (vs Australia) | T1 Alcohol Frequency | Regression | 0.42 | [0.06, 0.79] | **.023** |
| Age | T1 Alcohol Quantity | Regression | 0.01 | [0, 0.01] | .224 |
| Pre-COVID Frequency | T1 Alcohol Quantity | Regression | 0.12 | [-0.03, 0.27] | .108 |
| Pre-COVID Quantity | T1 Alcohol Quantity | Regression | -0.04 | [-0.23, 0.15] | .691 |
| U.K. (vs Australia) | T1 Alcohol Quantity | Regression | 0.21 | [-0.15, 0.57] | .245 |
| U.S. (vs Australia) | T1 Alcohol Quantity | Regression | 0.41 | [0.04, 0.78] | **.031** |
| Age | T2 Depression | Regression | -0.01 | [-0.04, 0.02] | .434 |
| Pre-COVID Frequency | T2 Depression | Regression | -0.10 | [-0.59, 0.39] | .687 |
| Pre-COVID Quantity | T2 Depression | Regression | -0.02 | [-0.79, 0.76] | .965 |
| U.K. (vs Australia) | T2 Depression | Regression | -0.51 | [-1.85, 0.84] | .461 |
| U.S. (vs Australia) | T2 Depression | Regression | 0.11 | [-1.25, 1.47] | .878 |
| Age | T2 Alcohol Frequency | Regression | 0.00 | [-0.01, 0] | .189 |
| Pre-COVID Frequency | T2 Alcohol Frequency | Regression | -0.04 | [-0.15, 0.07] | .432 |
| Pre-COVID Quantity | T2 Alcohol Frequency | Regression | 0.14 | [-0.05, 0.33] | .147 |
| U.K. (vs Australia) | T2 Alcohol Frequency | Regression | 0.09 | [-0.2, 0.38] | .526 |
| U.S. (vs Australia) | T2 Alcohol Frequency | Regression | -0.04 | [-0.34, 0.25] | .776 |
| Age | T2 Alcohol Quantity | Regression | 0.00 | [-0.01, 0] | .194 |
| Pre-COVID Frequency | T2 Alcohol Quantity | Regression | -0.02 | [-0.13, 0.09] | .676 |
| Pre-COVID Quantity | T2 Alcohol Quantity | Regression | 0.10 | [-0.12, 0.33] | .369 |
| U.K. (vs Australia) | T2 Alcohol Quantity | Regression | -0.17 | [-0.44, 0.1] | .221 |
| U.S. (vs Australia) | T2 Alcohol Quantity | Regression | -0.07 | [-0.35, 0.21] | .617 |
| Age | T3 Depression | Regression | -0.03 | [-0.06, 0] | **.033** |
| Pre-COVID Frequency | T3 Depression | Regression | 0.12 | [-0.42, 0.66] | .658 |
| Pre-COVID Quantity | T3 Depression | Regression | 0.38 | [-0.36, 1.11] | .317 |
| U.K. (vs Australia) | T3 Depression | Regression | 0.47 | [-0.67, 1.6] | .419 |
| U.S. (vs Australia) | T3 Depression | Regression | 0.48 | [-0.6, 1.56] | .382 |
| Age | T3 Alcohol Frequency | Regression | 0.00 | [0, 0.01] | .286 |
| Pre-COVID Frequency | T3 Alcohol Frequency | Regression | 0.04 | [-0.08, 0.17] | .474 |
| Pre-COVID Quantity | T3 Alcohol Frequency | Regression | -0.04 | [-0.23, 0.14] | .651 |
| U.K. (vs Australia) | T3 Alcohol Frequency | Regression | 0.07 | [-0.24, 0.37] | .673 |
| U.S. (vs Australia) | T3 Alcohol Frequency | Regression | 0.22 | [-0.08, 0.51] | .145 |
| Age | T3 Alcohol Quantity | Regression | 0.00 | [0, 0.01] | .359 |
| Pre-COVID Frequency | T3 Alcohol Quantity | Regression | 0.03 | [-0.1, 0.15] | .674 |
| Pre-COVID Quantity | T3 Alcohol Quantity | Regression | 0.07 | [-0.11, 0.25] | .428 |
| U.K. (vs Australia) | T3 Alcohol Quantity | Regression | 0.12 | [-0.17, 0.42] | .407 |
| U.S. (vs Australia) | T3 Alcohol Quantity | Regression | 0.08 | [-0.2, 0.37] | .566 |
| T1 Alcohol Frequency | T1 Depression | Covariance | 0.38 | [-0.3, 1.06] | .269 |
| T1 Alcohol Quantity | T1 Alcohol Frequency | Covariance | 0.92 | [0.75, 1.09] | **<.001** |
| T1 Alcohol Quantity | T1 Depression | Covariance | 0.48 | [-0.23, 1.19] | .188 |
| T2 Alcohol Frequency | T2 Depression | Covariance | 0.18 | [-0.3, 0.66] | .467 |
| T2 Alcohol Quantity | T2 Alcohol Frequency | Covariance | 0.41 | [0.29, 0.53] | **<.001** |
| T2 Alcohol Quantity | T2 Depression | Covariance | 0.19 | [-0.28, 0.66] | .425 |
| T3 Alcohol Frequency | T3 Depression | Covariance | 0.21 | [-0.2, 0.61] | .316 |
| T3 Alcohol Quantity | T3 Alcohol Frequency | Covariance | 0.53 | [0.38, 0.68] | **<.001** |
| T3 Alcohol Quantity | T3 Depression | Covariance | 0.34 | [-0.07, 0.75] | .109 |
| T1 Depression | T1 Depression | Variance | 28.40 | [23.23, 33.58] | **<.001** |
| T1 Alcohol Frequency | T1 Alcohol Frequency | Variance | 1.06 | [0.88, 1.25] | **<.001** |
| T1 Alcohol Quantity | T1 Alcohol Quantity | Variance | 1.05 | [0.88, 1.22] | **<.001** |
| T2 Depression | T2 Depression | Variance | 15.45 | [11.82, 19.08] | **<.001** |
| T2 Alcohol Frequency | T2 Alcohol Frequency | Variance | 0.61 | [0.47, 0.75] | **<.001** |
| T2 Alcohol Quantity | T2 Alcohol Quantity | Variance | 0.55 | [0.43, 0.68] | **<.001** |
| T3 Depression | T3 Depression | Variance | 11.71 | [8.35, 15.07] | **<.001** |
| T3 Alcohol Frequency | T3 Alcohol Frequency | Variance | 0.72 | [0.57, 0.87] | **<.001** |
| T3 Alcohol Quantity | T3 Alcohol Quantity | Variance | 0.64 | [0.48, 0.8] | **<.001** |

Model fit: *χ*^2^ (9) = 69.10, *p* < .001, Comparative Fit Index (CFI) = 0.96, Tucker-Lewis Index (TFI) = 0.68, Root Mean Square Error of Approximation (RMSEA) = 0.17, Standardized Root Mean Square Residual (SRMR) = 0.04.

**Table S8**

*Results for cross-lagged panel model examining associations between anxiety and alcohol consumption frequency and quantity*

| Predictor | Outcome | Path type | *b* | 95% CI | *p* |
| --- | --- | --- | --- | --- | --- |
| T1 Anxiety | T2 Anxiety | Regression | 0.72 | [0.61, 0.83] | **<.001** |
| T1 Alcohol Frequency | T2 Anxiety | Regression | -0.12 | [-0.94, 0.7] | .772 |
| T1 Alcohol Quantity | T2 Anxiety | Regression | 0.42 | [-0.37, 1.21] | .297 |
| T1 Anxiety | T2 Alcohol Frequency | Regression | -0.01 | [-0.02, 0.01] | .478 |
| T1 Alcohol Frequency | T2 Alcohol Frequency | Regression | 0.26 | [0.04, 0.48] | **.018** |
| T1 Alcohol Quantity | T2 Alcohol Frequency | Regression | 0.49 | [0.28, 0.7] | **<.001** |
| T1 Anxiety | T2 Alcohol Quantity | Regression | 0.00 | [-0.02, 0.02] | .839 |
| T1 Alcohol Frequency | T2 Alcohol Quantity | Regression | 0.25 | [0.05, 0.45] | **.014** |
| T1 Alcohol Quantity | T2 Alcohol Quantity | Regression | 0.45 | [0.25, 0.65] | **<.001** |
| T2 Anxiety | T3 Anxiety | Regression | 0.73 | [0.63, 0.83] | **<.001** |
| T2 Alcohol Frequency | T3 Anxiety | Regression | 0.04 | [-0.67, 0.76] | .902 |
| T2 Alcohol Quantity | T3 Anxiety | Regression | 0.23 | [-0.51, 0.97] | .539 |
| T2 Anxiety | T3 Alcohol Frequency | Regression | 0.00 | [-0.02, 0.02] | .814 |
| T2 Alcohol Frequency | T3 Alcohol Frequency | Regression | 0.43 | [0.21, 0.65] | **<.001** |
| T2 Alcohol Quantity | T3 Alcohol Frequency | Regression | 0.22 | [-0.02, 0.46] | .071 |
| T2 Anxiety | T3 Alcohol Quantity | Regression | 0.01 | [-0.01, 0.02] | .609 |
| T2 Alcohol Frequency | T3 Alcohol Quantity | Regression | 0.22 | [0.04, 0.4] | **.019** |
| T2 Alcohol Quantity | T3 Alcohol Quantity | Regression | 0.44 | [0.24, 0.64] | **<.001** |
| Age | T1 Anxiety | Regression | -0.09 | [-0.12, -0.05] | **<.001** |
| Pre-COVID Frequency | T1 Anxiety | Regression | -0.09 | [-0.73, 0.54] | .774 |
| Pre-COVID Quantity | T1 Anxiety | Regression | 1.03 | [-0.33, 2.4] | .138 |
| U.K. (vs Australia) | T1 Anxiety | Regression | 1.55 | [-0.1, 3.2] | .065 |
| U.S. (vs Australia) | T1 Anxiety | Regression | 2.08 | [0.32, 3.85] | **.021** |
| Age | T1 Alcohol Frequency | Regression | 0.00 | [0, 0.01] | .358 |
| Pre-COVID Frequency | T1 Alcohol Frequency | Regression | 0.05 | [-0.09, 0.19] | .511 |
| Pre-COVID Quantity | T1 Alcohol Frequency | Regression | 0.11 | [-0.06, 0.27] | .218 |
| U.K. (vs Australia) | T1 Alcohol Frequency | Regression | 0.21 | [-0.14, 0.57] | .236 |
| U.S. (vs Australia) | T1 Alcohol Frequency | Regression | 0.44 | [0.08, 0.8] | **.017** |
| Age | T1 Alcohol Quantity | Regression | 0.01 | [0, 0.01] | .245 |
| Pre-COVID Frequency | T1 Alcohol Quantity | Regression | 0.12 | [-0.03, 0.27] | .127 |
| Pre-COVID Quantity | T1 Alcohol Quantity | Regression | -0.05 | [-0.24, 0.15] | .638 |
| U.K. (vs Australia) | T1 Alcohol Quantity | Regression | 0.21 | [-0.15, 0.58] | .254 |
| U.S. (vs Australia) | T1 Alcohol Quantity | Regression | 0.39 | [0.02, 0.76] | **.041** |
| Age | T2 Anxiety | Regression | -0.02 | [-0.05, 0] | .087 |
| Pre-COVID Frequency | T2 Anxiety | Regression | -0.26 | [-0.7, 0.18] | .242 |
| Pre-COVID Quantity | T2 Anxiety | Regression | -0.65 | [-1.25, -0.05] | **.034** |
| U.K. (vs Australia) | T2 Anxiety | Regression | 0.67 | [-0.46, 1.79] | .244 |
| U.S. (vs Australia) | T2 Anxiety | Regression | -0.26 | [-1.43, 0.9] | .659 |
| Age | T2 Alcohol Frequency | Regression | -0.01 | [-0.01, 0] | .096 |
| Pre-COVID Frequency | T2 Alcohol Frequency | Regression | -0.06 | [-0.17, 0.05] | .319 |
| Pre-COVID Quantity | T2 Alcohol Frequency | Regression | 0.15 | [-0.04, 0.35] | .127 |
| U.K. (vs Australia) | T2 Alcohol Frequency | Regression | 0.11 | [-0.18, 0.39] | .468 |
| U.S. (vs Australia) | T2 Alcohol Frequency | Regression | -0.05 | [-0.35, 0.24] | .727 |
| Age | T2 Alcohol Quantity | Regression | -0.01 | [-0.01, 0] | .093 |
| Pre-COVID Frequency | T2 Alcohol Quantity | Regression | -0.04 | [-0.15, 0.07] | .488 |
| Pre-COVID Quantity | T2 Alcohol Quantity | Regression | 0.12 | [-0.12, 0.35] | .324 |
| U.K. (vs Australia) | T2 Alcohol Quantity | Regression | -0.17 | [-0.44, 0.11] | .234 |
| U.S. (vs Australia) | T2 Alcohol Quantity | Regression | -0.09 | [-0.37, 0.19] | .535 |
| Age | T3 Anxiety | Regression | -0.02 | [-0.05, 0.01] | .143 |
| Pre-COVID Frequency | T3 Anxiety | Regression | 0.13 | [-0.32, 0.57] | .576 |
| Pre-COVID Quantity | T3 Anxiety | Regression | 0.33 | [-0.39, 1.06] | .366 |
| U.K. (vs Australia) | T3 Anxiety | Regression | 0.51 | [-0.49, 1.5] | .318 |
| U.S. (vs Australia) | T3 Anxiety | Regression | 1.07 | [-0.04, 2.19] | .059 |
| Age | T3 Alcohol Frequency | Regression | 0.00 | [0, 0.01] | .230 |
| Pre-COVID Frequency | T3 Alcohol Frequency | Regression | 0.05 | [-0.08, 0.17] | .457 |
| Pre-COVID Quantity | T3 Alcohol Frequency | Regression | -0.05 | [-0.25, 0.14] | .574 |
| U.K. (vs Australia) | T3 Alcohol Frequency | Regression | 0.05 | [-0.26, 0.36] | .758 |
| U.S. (vs Australia) | T3 Alcohol Frequency | Regression | 0.20 | [-0.1, 0.49] | .189 |
| Age | T3 Alcohol Quantity | Regression | 0.00 | [0, 0.01] | .268 |
| Pre-COVID Frequency | T3 Alcohol Quantity | Regression | 0.03 | [-0.09, 0.15] | .632 |
| Pre-COVID Quantity | T3 Alcohol Quantity | Regression | 0.07 | [-0.12, 0.25] | .475 |
| U.K. (vs Australia) | T3 Alcohol Quantity | Regression | 0.11 | [-0.19, 0.4] | .483 |
| U.S. (vs Australia) | T3 Alcohol Quantity | Regression | 0.06 | [-0.22, 0.34] | .673 |
| T1 Alcohol Frequency | T1 Anxiety | Covariance | 0.26 | [-0.47, 0.99] | .484 |
| T1 Alcohol Quantity | T1 Alcohol Frequency | Covariance | 0.92 | [0.75, 1.09] | **<.001** |
| T1 Alcohol Quantity | T1 Anxiety | Covariance | 0.32 | [-0.42, 1.06] | .397 |
| T2 Alcohol Frequency | T2 Anxiety | Covariance | 0.34 | [-0.08, 0.76] | .111 |
| T2 Alcohol Quantity | T2 Alcohol Frequency | Covariance | 0.43 | [0.31, 0.55] | **<.001** |
| T2 Alcohol Quantity | T2 Anxiety | Covariance | 0.32 | [-0.05, 0.69] | .092 |
| T3 Alcohol Frequency | T3 Anxiety | Covariance | 0.21 | [-0.15, 0.58] | .252 |
| T3 Alcohol Quantity | T3 Alcohol Frequency | Covariance | 0.54 | [0.38, 0.69] | **<.001** |
| T3 Alcohol Quantity | T3 Anxiety | Covariance | 0.34 | [0, 0.68] | .051 |
| T1 Anxiety | T1 Anxiety | Variance | 26.41 | [21.25, 31.57] | **<.001** |
| T1 Alcohol Frequency | T1 Alcohol Frequency | Variance | 1.07 | [0.89, 1.25] | **<.001** |
| T1 Alcohol Quantity | T1 Alcohol Quantity | Variance | 1.07 | [0.89, 1.24] | **<.001** |
| T2 Anxiety | T2 Anxiety | Variance | 12.29 | [9.5, 15.08] | **<.001** |
| T2 Alcohol Frequency | T2 Alcohol Frequency | Variance | 0.62 | [0.48, 0.76] | **<.001** |
| T2 Alcohol Quantity | T2 Alcohol Quantity | Variance | 0.58 | [0.45, 0.7] | **<.001** |
| T3 Anxiety | T3 Anxiety | Variance | 10.53 | [7.92, 13.15] | **<.001** |
| T3 Alcohol Frequency | T3 Alcohol Frequency | Variance | 0.73 | [0.57, 0.88] | **<.001** |
| T3 Alcohol Quantity | T3 Alcohol Quantity | Variance | 0.64 | [0.48, 0.81] | **<.001** |

Model fit: *χ*^2^ (9) = 68.32, *p* < .001, CFI = 0.96, TFI = 0.68, RMSEA = 0.17, SRMR = 0.04

**Table S9**

*Results for cross-lagged panel model examining associations between pandemic-related stress and alcohol consumption frequency and quantity*

| Predictor | Outcome | Path type | *b* | 95% CI | *p* |
| --- | --- | --- | --- | --- | --- |
| T1 Pandemic Stress | T2 Pandemic Stress | Regression | 0.79 | [0.69, 0.88] | **<.001** |
| T1 Alcohol Frequency | T2 Pandemic Stress | Regression | 0.04 | [-0.9, 0.98] | .929 |
| T1 Alcohol Quantity | T2 Pandemic Stress | Regression | -0.05 | [-1.03, 0.93] | .924 |
| T1 Pandemic Stress | T2 Alcohol Frequency | Regression | -0.01 | [-0.03, 0] | .089 |
| T1 Alcohol Frequency | T2 Alcohol Frequency | Regression | 0.24 | [0.02, 0.46] | **.031** |
| T1 Alcohol Quantity | T2 Alcohol Frequency | Regression | 0.50 | [0.29, 0.72] | **<.001** |
| T1 Pandemic Stress | T2 Alcohol Quantity | Regression | -0.01 | [-0.02, 0.01] | .322 |
| T1 Alcohol Frequency | T2 Alcohol Quantity | Regression | 0.24 | [0.04, 0.44] | **.019** |
| T1 Alcohol Quantity | T2 Alcohol Quantity | Regression | 0.46 | [0.25, 0.66] | **<.001** |
| T2 Pandemic Stress | T3 Pandemic Stress | Regression | 0.75 | [0.67, 0.83] | **<.001** |
| T2 Alcohol Frequency | T3 Pandemic Stress | Regression | 0.22 | [-0.57, 1] | .585 |
| T2 Alcohol Quantity | T3 Pandemic Stress | Regression | -0.30 | [-1.17, 0.57] | .501 |
| T2 Pandemic Stress | T3 Alcohol Frequency | Regression | 0.00 | [-0.02, 0.01] | .813 |
| T2 Alcohol Frequency | T3 Alcohol Frequency | Regression | 0.44 | [0.21, 0.66] | **<.001** |
| T2 Alcohol Quantity | T3 Alcohol Frequency | Regression | 0.22 | [-0.01, 0.46] | .066 |
| T2 Pandemic Stress | T3 Alcohol Quantity | Regression | 0.00 | [-0.02, 0.01] | .598 |
| T2 Alcohol Frequency | T3 Alcohol Quantity | Regression | 0.20 | [0.02, 0.38] | **.030** |
| T2 Alcohol Quantity | T3 Alcohol Quantity | Regression | 0.47 | [0.27, 0.66] | **<.001** |
| Age | T1 Pandemic Stress | Regression | -0.08 | [-0.12, -0.04] | **<.001** |
| Pre-COVID Frequency | T1 Pandemic Stress | Regression | -0.43 | [-1.2, 0.34] | .270 |
| Pre-COVID Quantity | T1 Pandemic Stress | Regression | 0.53 | [-0.71, 1.76] | .404 |
| U.K. (vs Australia) | T1 Pandemic Stress | Regression | 1.38 | [-0.62, 3.38] | .178 |
| U.S. (vs Australia) | T1 Pandemic Stress | Regression | 2.64 | [0.56, 4.72] | **.013** |
| Age | T1 Alcohol Frequency | Regression | 0.00 | [0, 0.01] | .324 |
| Pre-COVID Frequency | T1 Alcohol Frequency | Regression | 0.04 | [-0.1, 0.18] | .580 |
| Pre-COVID Quantity | T1 Alcohol Frequency | Regression | 0.12 | [-0.05, 0.28] | .170 |
| U.K. (vs Australia) | T1 Alcohol Frequency | Regression | 0.15 | [-0.19, 0.5] | .386 |
| U.S. (vs Australia) | T1 Alcohol Frequency | Regression | 0.43 | [0.07, 0.79] | **.018** |
| Age | T1 Alcohol Quantity | Regression | 0.01 | [0, 0.01] | .226 |
| Pre-COVID Frequency | T1 Alcohol Quantity | Regression | 0.12 | [-0.03, 0.27] | .107 |
| Pre-COVID Quantity | T1 Alcohol Quantity | Regression | -0.04 | [-0.23, 0.15] | .707 |
| U.K. (vs Australia) | T1 Alcohol Quantity | Regression | 0.16 | [-0.2, 0.51] | .385 |
| U.S. (vs Australia) | T1 Alcohol Quantity | Regression | 0.40 | [0.03, 0.77] | **.033** |
| Age | T2 Pandemic Stress | Regression | -0.01 | [-0.05, 0.02] | .476 |
| Pre-COVID Frequency | T2 Pandemic Stress | Regression | -0.02 | [-0.6, 0.57] | .953 |
| Pre-COVID Quantity | T2 Pandemic Stress | Regression | -0.25 | [-1.1, 0.59] | .556 |
| U.K. (vs Australia) | T2 Pandemic Stress | Regression | -0.22 | [-1.7, 1.26] | .770 |
| U.S. (vs Australia) | T2 Pandemic Stress | Regression | -0.68 | [-2.19, 0.84] | .380 |
| Age | T2 Alcohol Frequency | Regression | -0.01 | [-0.01, 0] | .063 |
| Pre-COVID Frequency | T2 Alcohol Frequency | Regression | -0.06 | [-0.17, 0.06] | .328 |
| Pre-COVID Quantity | T2 Alcohol Frequency | Regression | 0.14 | [-0.04, 0.33] | .131 |
| U.K. (vs Australia) | T2 Alcohol Frequency | Regression | 0.19 | [-0.1, 0.48] | .203 |
| U.S. (vs Australia) | T2 Alcohol Frequency | Regression | 0.01 | [-0.3, 0.31] | .958 |
| Age | T2 Alcohol Quantity | Regression | -0.01 | [-0.01, 0] | .067 |
| Pre-COVID Frequency | T2 Alcohol Quantity | Regression | -0.03 | [-0.15, 0.08] | .550 |
| Pre-COVID Quantity | T2 Alcohol Quantity | Regression | 0.12 | [-0.11, 0.34] | .300 |
| U.K. (vs Australia) | T2 Alcohol Quantity | Regression | -0.08 | [-0.36, 0.2] | .577 |
| U.S. (vs Australia) | T2 Alcohol Quantity | Regression | -0.03 | [-0.32, 0.27] | .851 |
| Age | T3 Pandemic Stress | Regression | -0.03 | [-0.06, -0.01] | **.014** |
| Pre-COVID Frequency | T3 Pandemic Stress | Regression | -0.01 | [-0.46, 0.45] | .976 |
| Pre-COVID Quantity | T3 Pandemic Stress | Regression | 0.27 | [-0.49, 1.03] | .490 |
| U.K. (vs Australia) | T3 Pandemic Stress | Regression | 1.48 | [0.28, 2.68] | **.016** |
| U.S. (vs Australia) | T3 Pandemic Stress | Regression | 2.49 | [1.23, 3.75] | **<.001** |
| Age | T3 Alcohol Frequency | Regression | 0.00 | [0, 0.01] | .253 |
| Pre-COVID Frequency | T3 Alcohol Frequency | Regression | 0.04 | [-0.08, 0.16] | .536 |
| Pre-COVID Quantity | T3 Alcohol Frequency | Regression | -0.04 | [-0.23, 0.14] | .642 |
| U.K. (vs Australia) | T3 Alcohol Frequency | Regression | 0.05 | [-0.25, 0.35] | .752 |
| U.S. (vs Australia) | T3 Alcohol Frequency | Regression | 0.19 | [-0.11, 0.48] | .209 |
| Age | T3 Alcohol Quantity | Regression | 0.00 | [0, 0.01] | .333 |
| Pre-COVID Frequency | T3 Alcohol Quantity | Regression | 0.02 | [-0.1, 0.14] | .757 |
| Pre-COVID Quantity | T3 Alcohol Quantity | Regression | 0.07 | [-0.11, 0.25] | .423 |
| U.K. (vs Australia) | T3 Alcohol Quantity | Regression | 0.10 | [-0.19, 0.39] | .513 |
| U.S. (vs Australia) | T3 Alcohol Quantity | Regression | 0.06 | [-0.23, 0.34] | .698 |
| T1 Alcohol Frequency | T1 Pandemic Stress | Covariance | 0.31 | [-0.4, 1.03] | .392 |
| T1 Alcohol Quantity | T1 Alcohol Frequency | Covariance | 0.93 | [0.76, 1.1] | **<.001** |
| T1 Alcohol Quantity | T1 Pandemic Stress | Covariance | 0.63 | [-0.12, 1.37] | .098 |
| T2 Alcohol Frequency | T2 Pandemic Stress | Covariance | 0.18 | [-0.32, 0.67] | .485 |
| T2 Alcohol Quantity | T2 Alcohol Frequency | Covariance | 0.44 | [0.32, 0.55] | **<.001** |
| T2 Alcohol Quantity | T2 Pandemic Stress | Covariance | 0.23 | [-0.24, 0.71] | .334 |
| T3 Alcohol Frequency | T3 Pandemic Stress | Covariance | 0.00 | [-0.35, 0.35] | .993 |
| T3 Alcohol Quantity | T3 Alcohol Frequency | Covariance | 0.53 | [0.38, 0.68] | **<.001** |
| T3 Alcohol Quantity | T3 Pandemic Stress | Covariance | 0.04 | [-0.29, 0.37] | .803 |
| T1 Pandemic Stress | T1 Pandemic Stress | Variance | 30.80 | [25.11, 36.49] | **<.001** |
| T1 Alcohol Frequency | T1 Alcohol Frequency | Variance | 1.08 | [0.9, 1.26] | **<.001** |
| T1 Alcohol Quantity | T1 Alcohol Quantity | Variance | 1.08 | [0.91, 1.25] | **<.001** |
| T2 Pandemic Stress | T2 Pandemic Stress | Variance | 16.02 | [12.48, 19.57] | **<.001** |
| T2 Alcohol Frequency | T2 Alcohol Frequency | Variance | 0.63 | [0.49, 0.76] | **<.001** |
| T2 Alcohol Quantity | T2 Alcohol Quantity | Variance | 0.58 | [0.46, 0.7] | **<.001** |
| T3 Pandemic Stress | T3 Pandemic Stress | Variance | 12.42 | [9.79, 15.05] | **<.001** |
| T3 Alcohol Frequency | T3 Alcohol Frequency | Variance | 0.72 | [0.57, 0.88] | **<.001** |
| T3 Alcohol Quantity | T3 Alcohol Quantity | Variance | 0.64 | [0.48, 0.8] | **<.001** |

Model fit: *χ*^2^ (9) = 52.57, *p* < .001, CFI = 0.98, TFI = 0.77, RMSEA = 0.14, SRMR = 0.04.

**Table S10**

*Results for cross-lagged panel model examining associations between general stress and alcohol consumption frequency and quantity*

| Predictor | Outcome | Path type | *b* | 95% CI | *p* |
| --- | --- | --- | --- | --- | --- |
| T1 General Stress | T2 General Stress | Regression | 0.66 | [0.55, 0.78] | **<.001** |
| T1 Alcohol Frequency | T2 General Stress | Regression | 0.62 | [0.04, 1.19] | **.036** |
| T1 Alcohol Quantity | T2 General Stress | Regression | -0.30 | [-0.9, 0.29] | .314 |
| T1 General Stress | T2 Alcohol Frequency | Regression | 0.01 | [-0.03, 0.04] | .703 |
| T1 Alcohol Frequency | T2 Alcohol Frequency | Regression | 0.25 | [0.03, 0.48] | **.024** |
| T1 Alcohol Quantity | T2 Alcohol Frequency | Regression | 0.48 | [0.26, 0.7] | **<.001** |
| T1 General Stress | T2 Alcohol Quantity | Regression | 0.00 | [-0.04, 0.03] | .832 |
| T1 Alcohol Frequency | T2 Alcohol Quantity | Regression | 0.24 | [0.04, 0.44] | **.016** |
| T1 Alcohol Quantity | T2 Alcohol Quantity | Regression | 0.45 | [0.24, 0.65] | **<.001** |
| T2 General Stress | T3 General Stress | Regression | 0.50 | [0.4, 0.61] | **<.001** |
| T2 Alcohol Frequency | T3 General Stress | Regression | -0.08 | [-0.63, 0.46] | .765 |
| T2 Alcohol Quantity | T3 General Stress | Regression | 0.22 | [-0.35, 0.8] | .445 |
| T2 General Stress | T3 Alcohol Frequency | Regression | 0.02 | [-0.02, 0.05] | .326 |
| T2 Alcohol Frequency | T3 Alcohol Frequency | Regression | 0.43 | [0.21, 0.65] | **<.001** |
| T2 Alcohol Quantity | T3 Alcohol Frequency | Regression | 0.22 | [-0.02, 0.45] | .070 |
| T2 General Stress | T3 Alcohol Quantity | Regression | 0.01 | [-0.02, 0.04] | .518 |
| T2 Alcohol Frequency | T3 Alcohol Quantity | Regression | 0.20 | [0.02, 0.38] | **.029** |
| T2 Alcohol Quantity | T3 Alcohol Quantity | Regression | 0.46 | [0.26, 0.66] | **<.001** |
| Age | T1 General Stress | Regression | -0.05 | [-0.07, -0.02] | **<.001** |
| Pre-COVID Frequency | T1 General Stress | Regression | -0.08 | [-0.51, 0.36] | .732 |
| Pre-COVID Quantity | T1 General Stress | Regression | 0.43 | [-0.37, 1.23] | .289 |
| U.K. (vs Australia) | T1 General Stress | Regression | 1.40 | [0.42, 2.39] | **.005** |
| U.S. (vs Australia) | T1 General Stress | Regression | 0.90 | [-0.21, 2.01] | .113 |
| Age | T1 Alcohol Frequency | Regression | 0.00 | [0, 0.01] | .293 |
| Pre-COVID Frequency | T1 Alcohol Frequency | Regression | 0.04 | [-0.1, 0.18] | .605 |
| Pre-COVID Quantity | T1 Alcohol Frequency | Regression | 0.11 | [-0.05, 0.28] | .186 |
| U.K. (vs Australia) | T1 Alcohol Frequency | Regression | 0.16 | [-0.18, 0.51] | .354 |
| U.S. (vs Australia) | T1 Alcohol Frequency | Regression | 0.43 | [0.07, 0.78] | **.019** |
| Age | T1 Alcohol Quantity | Regression | 0.01 | [0, 0.01] | .195 |
| Pre-COVID Frequency | T1 Alcohol Quantity | Regression | 0.12 | [-0.03, 0.27] | .116 |
| Pre-COVID Quantity | T1 Alcohol Quantity | Regression | -0.04 | [-0.23, 0.15] | .670 |
| U.K. (vs Australia) | T1 Alcohol Quantity | Regression | 0.17 | [-0.19, 0.53] | .352 |
| U.S. (vs Australia) | T1 Alcohol Quantity | Regression | 0.40 | [0.03, 0.77] | **.034** |
| Age | T2 General Stress | Regression | 0.00 | [-0.02, 0.02] | .700 |
| Pre-COVID Frequency | T2 General Stress | Regression | -0.12 | [-0.47, 0.23] | .492 |
| Pre-COVID Quantity | T2 General Stress | Regression | -0.07 | [-0.74, 0.6] | .837 |
| U.K. (vs Australia) | T2 General Stress | Regression | -0.42 | [-1.29, 0.44] | .341 |
| U.S. (vs Australia) | T2 General Stress | Regression | -0.37 | [-1.21, 0.47] | .389 |
| Age | T2 Alcohol Frequency | Regression | 0.00 | [-0.01, 0] | .143 |
| Pre-COVID Frequency | T2 Alcohol Frequency | Regression | -0.04 | [-0.16, 0.07] | .439 |
| Pre-COVID Quantity | T2 Alcohol Frequency | Regression | 0.13 | [-0.05, 0.32] | .163 |
| U.K. (vs Australia) | T2 Alcohol Frequency | Regression | 0.16 | [-0.14, 0.45] | .301 |
| U.S. (vs Australia) | T2 Alcohol Frequency | Regression | -0.03 | [-0.33, 0.28] | .853 |
| Age | T2 Alcohol Quantity | Regression | -0.01 | [-0.01, 0] | .062 |
| Pre-COVID Frequency | T2 Alcohol Quantity | Regression | -0.03 | [-0.14, 0.08] | .616 |
| Pre-COVID Quantity | T2 Alcohol Quantity | Regression | 0.12 | [-0.11, 0.35] | .320 |
| U.K. (vs Australia) | T2 Alcohol Quantity | Regression | -0.09 | [-0.37, 0.19] | .529 |
| U.S. (vs Australia) | T2 Alcohol Quantity | Regression | -0.04 | [-0.33, 0.25] | .785 |
| Age | T3 General Stress | Regression | -0.02 | [-0.04, -0.01] | **.009** |
| Pre-COVID Frequency | T3 General Stress | Regression | 0.13 | [-0.22, 0.47] | .466 |
| Pre-COVID Quantity | T3 General Stress | Regression | 0.01 | [-0.66, 0.69] | .970 |
| U.K. (vs Australia) | T3 General Stress | Regression | 1.04 | [0.18, 1.9] | **.018** |
| U.S. (vs Australia) | T3 General Stress | Regression | 0.41 | [-0.47, 1.29] | .360 |
| Age | T3 Alcohol Frequency | Regression | 0.00 | [0, 0.01] | .187 |
| Pre-COVID Frequency | T3 Alcohol Frequency | Regression | 0.04 | [-0.08, 0.16] | .494 |
| Pre-COVID Quantity | T3 Alcohol Frequency | Regression | -0.05 | [-0.23, 0.14] | .613 |
| U.K. (vs Australia) | T3 Alcohol Frequency | Regression | 0.04 | [-0.26, 0.34] | .793 |
| U.S. (vs Australia) | T3 Alcohol Frequency | Regression | 0.18 | [-0.11, 0.47] | .225 |
| Age | T3 Alcohol Quantity | Regression | 0.00 | [0, 0.01] | .247 |
| Pre-COVID Frequency | T3 Alcohol Quantity | Regression | 0.02 | [-0.1, 0.14] | .723 |
| Pre-COVID Quantity | T3 Alcohol Quantity | Regression | 0.07 | [-0.11, 0.25] | .438 |
| U.K. (vs Australia) | T3 Alcohol Quantity | Regression | 0.09 | [-0.2, 0.38] | .549 |
| U.S. (vs Australia) | T3 Alcohol Quantity | Regression | 0.05 | [-0.23, 0.33] | .731 |
| T1 Alcohol Frequency | T1 General Stress | Covariance | 0.20 | [-0.21, 0.62] | .330 |
| T1 Alcohol Quantity | T1 Alcohol Frequency | Covariance | 0.93 | [0.76, 1.1] | **<.001** |
| T1 Alcohol Quantity | T1 General Stress | Covariance | 0.29 | [-0.15, 0.73] | .192 |
| T2 Alcohol Frequency | T2 General Stress | Covariance | 0.28 | [0.04, 0.52] | **.024** |
| T2 Alcohol Quantity | T2 Alcohol Frequency | Covariance | 0.44 | [0.32, 0.56] | **<.001** |
| T2 Alcohol Quantity | T2 General Stress | Covariance | 0.32 | [0.09, 0.56] | **.007** |
| T3 Alcohol Frequency | T3 General Stress | Covariance | -0.06 | [-0.37, 0.25] | .709 |
| T3 Alcohol Quantity | T3 Alcohol Frequency | Covariance | 0.52 | [0.37, 0.67] | **<.001** |
| T3 Alcohol Quantity | T3 General Stress | Covariance | 0.01 | [-0.28, 0.3] | .948 |
| T1 General Stress | T1 General Stress | Variance | 9.55 | [7.94, 11.16] | **<.001** |
| T1 Alcohol Frequency | T1 Alcohol Frequency | Variance | 1.08 | [0.9, 1.26] | **<.001** |
| T1 Alcohol Quantity | T1 Alcohol Quantity | Variance | 1.08 | [0.91, 1.25] | **<.001** |
| T2 General Stress | T2 General Stress | Variance | 6.28 | [5.06, 7.5] | **<.001** |
| T2 Alcohol Frequency | T2 Alcohol Frequency | Variance | 0.63 | [0.49, 0.77] | **<.001** |
| T2 Alcohol Quantity | T2 Alcohol Quantity | Variance | 0.58 | [0.46, 0.7] | **<.001** |
| T3 General Stress | T3 General Stress | Variance | 6.15 | [4.9, 7.4] | **<.001** |
| T3 Alcohol Frequency | T3 Alcohol Frequency | Variance | 0.72 | [0.57, 0.87] | **<.001** |
| T3 Alcohol Quantity | T3 Alcohol Quantity | Variance | 0.64 | [0.48, 0.79] | **<.001** |

Model fit: *χ*^2^ (9) = 49.216, *p* < .001, CFI = 0.97, TFI = 0.77, RMSEA = 0.14, SRMR = 0.04

**Table S11**

*Results for cross-lagged panel model examining associations between loneliness and alcohol consumption frequency and quantity*

| Predictor | Outcome | Path type | *b* | 95% CI | *p* |
| --- | --- | --- | --- | --- | --- |
| T1 Loneliness | T2 Loneliness | Regression | 0.80 | [0.71, 0.89] | **<.001** |
| T1 Alcohol Frequency | T2 Loneliness | Regression | 0.46 | [-1.78, 2.7] | .685 |
| T1 Alcohol Quantity | T2 Loneliness | Regression | -0.06 | [-2.24, 2.12] | .957 |
| T1 Loneliness | T2 Alcohol Frequency | Regression | 0.00 | [0, 0.01] | .169 |
| T1 Alcohol Frequency | T2 Alcohol Frequency | Regression | 0.25 | [0.03, 0.47] | **.023** |
| T1 Alcohol Quantity | T2 Alcohol Frequency | Regression | 0.48 | [0.27, 0.69] | **<.001** |
| T1 Loneliness | T2 Alcohol Quantity | Regression | 0.01 | [0, 0.01] | **.046** |
| T1 Alcohol Frequency | T2 Alcohol Quantity | Regression | 0.25 | [0.05, 0.45] | **.016** |
| T1 Alcohol Quantity | T2 Alcohol Quantity | Regression | 0.44 | [0.24, 0.65] | **<.001** |
| T2 Loneliness | T3 Loneliness | Regression | 0.80 | [0.73, 0.88] | **<.001** |
| T2 Alcohol Frequency | T3 Loneliness | Regression | -0.61 | [-2.57, 1.36] | .544 |
| T2 Alcohol Quantity | T3 Loneliness | Regression | -0.06 | [-1.93, 1.81] | .950 |
| T2 Loneliness | T3 Alcohol Frequency | Regression | -0.01 | [-0.01, 0] | .069 |
| T2 Alcohol Frequency | T3 Alcohol Frequency | Regression | 0.45 | [0.23, 0.67] | **<.001** |
| T2 Alcohol Quantity | T3 Alcohol Frequency | Regression | 0.23 | [-0.01, 0.46] | .061 |
| T2 Loneliness | T3 Alcohol Quantity | Regression | -0.01 | [-0.01, 0] | **.016** |
| T2 Alcohol Frequency | T3 Alcohol Quantity | Regression | 0.22 | [0.04, 0.4] | **.019** |
| T2 Alcohol Quantity | T3 Alcohol Quantity | Regression | 0.47 | [0.27, 0.66] | **<.001** |
| Age | T1 Loneliness | Regression | -0.15 | [-0.25, -0.04] | **.006** |
| Pre-COVID Frequency | T1 Loneliness | Regression | -1.52 | [-3.45, 0.41] | .123 |
| Pre-COVID Quantity | T1 Loneliness | Regression | 2.77 | [-1.22, 6.76] | .174 |
| U.K. (vs Australia) | T1 Loneliness | Regression | 1.10 | [-3.19, 5.4] | .615 |
| U.S. (vs Australia) | T1 Loneliness | Regression | 2.53 | [-2.49, 7.55] | .323 |
| Age | T1 Alcohol Frequency | Regression | 0.00 | [0, 0.01] | .294 |
| Pre-COVID Frequency | T1 Alcohol Frequency | Regression | 0.04 | [-0.1, 0.18] | .605 |
| Pre-COVID Quantity | T1 Alcohol Frequency | Regression | 0.11 | [-0.06, 0.28] | .195 |
| U.K. (vs Australia) | T1 Alcohol Frequency | Regression | 0.17 | [-0.18, 0.52] | .351 |
| U.S. (vs Australia) | T1 Alcohol Frequency | Regression | 0.43 | [0.07, 0.79] | **.020** |
| Age | T1 Alcohol Quantity | Regression | 0.01 | [0, 0.01] | .192 |
| Pre-COVID Frequency | T1 Alcohol Quantity | Regression | 0.12 | [-0.03, 0.27] | .116 |
| Pre-COVID Quantity | T1 Alcohol Quantity | Regression | -0.04 | [-0.23, 0.15] | .650 |
| U.K. (vs Australia) | T1 Alcohol Quantity | Regression | 0.18 | [-0.18, 0.54] | .337 |
| U.S. (vs Australia) | T1 Alcohol Quantity | Regression | 0.40 | [0.03, 0.78] | **.034** |
| Age | T2 Loneliness | Regression | -0.02 | [-0.1, 0.06] | .588 |
| Pre-COVID Frequency | T2 Loneliness | Regression | 0.32 | [-0.94, 1.59] | .617 |
| Pre-COVID Quantity | T2 Loneliness | Regression | 0.35 | [-1.25, 1.95] | .671 |
| U.K. (vs Australia) | T2 Loneliness | Regression | -3.21 | [-6.36, -0.06] | **.046** |
| U.S. (vs Australia) | T2 Loneliness | Regression | -3.50 | [-7.08, 0.08] | **.055** |
| Age | T2 Alcohol Frequency | Regression | 0.00 | [-0.01, 0] | .183 |
| Pre-COVID Frequency | T2 Alcohol Frequency | Regression | -0.04 | [-0.15, 0.08] | .524 |
| Pre-COVID Quantity | T2 Alcohol Frequency | Regression | 0.12 | [-0.07, 0.32] | .203 |
| U.K. (vs Australia) | T2 Alcohol Frequency | Regression | 0.16 | [-0.14, 0.45] | .293 |
| U.S. (vs Australia) | T2 Alcohol Frequency | Regression | -0.05 | [-0.36, 0.26] | .744 |
| Age | T2 Alcohol Quantity | Regression | 0.00 | [-0.01, 0] | .160 |
| Pre-COVID Frequency | T2 Alcohol Quantity | Regression | -0.02 | [-0.13, 0.1] | .763 |
| Pre-COVID Quantity | T2 Alcohol Quantity | Regression | 0.10 | [-0.13, 0.33] | .400 |
| U.K. (vs Australia) | T2 Alcohol Quantity | Regression | -0.10 | [-0.38, 0.17] | .462 |
| U.S. (vs Australia) | T2 Alcohol Quantity | Regression | -0.08 | [-0.36, 0.21] | .605 |
| Age | T3 Loneliness | Regression | -0.05 | [-0.13, 0.03] | .203 |
| Pre-COVID Frequency | T3 Loneliness | Regression | 0.10 | [-1.15, 1.34] | .881 |
| Pre-COVID Quantity | T3 Loneliness | Regression | 0.51 | [-1.39, 2.4] | .601 |
| U.K. (vs Australia) | T3 Loneliness | Regression | 4.88 | [1.56, 8.2] | **.004** |
| U.S. (vs Australia) | T3 Loneliness | Regression | 4.07 | [0.84, 7.31] | **.014** |
| Age | T3 Alcohol Frequency | Regression | 0.00 | [0, 0.01] | .377 |
| Pre-COVID Frequency | T3 Alcohol Frequency | Regression | 0.03 | [-0.09, 0.15] | .584 |
| Pre-COVID Quantity | T3 Alcohol Frequency | Regression | -0.02 | [-0.2, 0.17] | .868 |
| U.K. (vs Australia) | T3 Alcohol Frequency | Regression | -0.01 | [-0.31, 0.29] | .956 |
| U.S. (vs Australia) | T3 Alcohol Frequency | Regression | 0.15 | [-0.14, 0.43] | .316 |
| Age | T3 Alcohol Quantity | Regression | 0.00 | [0, 0.01] | .467 |
| Pre-COVID Frequency | T3 Alcohol Quantity | Regression | 0.01 | [-0.11, 0.13] | .835 |
| Pre-COVID Quantity | T3 Alcohol Quantity | Regression | 0.10 | [-0.09, 0.28] | .303 |
| U.K. (vs Australia) | T3 Alcohol Quantity | Regression | 0.05 | [-0.24, 0.35] | .717 |
| U.S. (vs Australia) | T3 Alcohol Quantity | Regression | 0.03 | [-0.25, 0.31] | .850 |
| T1 Alcohol Frequency | T1 Loneliness | Covariance | 0.36 | [-1.6, 2.31] | .722 |
| T1 Alcohol Quantity | T1 Alcohol Frequency | Covariance | 0.94 | [0.77, 1.11] | **<.001** |
| T1 Alcohol Quantity | T1 Loneliness | Covariance | 0.36 | [-1.55, 2.27] | .711 |
| T2 Alcohol Frequency | T2 Loneliness | Covariance | 1.02 | [0.03, 2.02] | **.044** |
| T2 Alcohol Quantity | T2 Alcohol Frequency | Covariance | 0.43 | [0.31, 0.55] | **<.001** |
| T2 Alcohol Quantity | T2 Loneliness | Covariance | 0.43 | [-0.51, 1.37] | .372 |
| T3 Alcohol Frequency | T3 Loneliness | Covariance | 1.17 | [-0.03, 2.36] | .056 |
| T3 Alcohol Quantity | T3 Alcohol Frequency | Covariance | 0.51 | [0.36, 0.66] | **<.001** |
| T3 Alcohol Quantity | T3 Loneliness | Covariance | 1.31 | [0.16, 2.47] | **.026** |
| T1 Loneliness | T1 Loneliness | Variance | 191.05 | [161.75, 220.34] | **<.001** |
| T1 Alcohol Frequency | T1 Alcohol Frequency | Variance | 1.09 | [0.91, 1.27] | **<.001** |
| T1 Alcohol Quantity | T1 Alcohol Quantity | Variance | 1.09 | [0.92, 1.26] | **<.001** |
| T2 Loneliness | T2 Loneliness | Variance | 86.01 | [65.29, 106.74] | **<.001** |
| T2 Alcohol Frequency | T2 Alcohol Frequency | Variance | 0.63 | [0.49, 0.76] | **<.001** |
| T2 Alcohol Quantity | T2 Alcohol Quantity | Variance | 0.57 | [0.45, 0.69] | **<.001** |
| T3 Loneliness | T3 Loneliness | Variance | 89.89 | [61.24, 118.53] | **<.001** |
| T3 Alcohol Frequency | T3 Alcohol Frequency | Variance | 0.70 | [0.55, 0.85] | **<.001** |
| T3 Alcohol Quantity | T3 Alcohol Quantity | Variance | 0.63 | [0.47, 0.78] | **<.001** |

Model fit: *χ*^2^ (9) = 55.17, *p* < .001, CFI = 0.97, TFI = 0.75, RMSEA = 0.15, SRMR = 0.04

## Sensitivity Analysis Results

### Results accounting for pandemic-related stress

**Table S12**

*Results for associations between pre-pandemic alcohol consumption quantity and frequency and relative changes in consumption at T1*

| Variable | Odds | 95% CI | *t* | *p* |
| --- | --- | --- | --- | --- |
| Quantity Model |  |  |  |  |
| Pre-pandemic quantity level |  |  |  |  |
| 3-4 vs 1-2 std drinks | 1.41 | [1.03, 1.95] | 2.11 | **.035** |
| 5-6 vs 1-2 std drinks | 1.41 | [0.81, 2.46] | 1.20 | .229 |
| 7-9 vs 1-2 std drinks | 0.99 | [0.38, 2.56] | -0.01 | .988 |
| 10 or more vs 1-2 std drinks | 2.13 | [0.44, 10.53] | 0.94 | .346 |
| Country |  |  |  |  |
| U.K. vs Australia | 1.40 | [1.08, 1.83] | 2.52 | **.012** |
| U.S. vs Australia | 1.63 | [1.2, 2.22] | 3.15 | **.002** |
| Pandemic-related stress | 1.03 | [1.01, 1.05] | 2.92 | **.004** |
| Frequency Model |  |  |  |  |
| Pre-pandemic frequency level |  |  |  |  |
| Monthly or less vs never | 0.69 | [0.3, 1.59] | -0.88 | .377 |
| 2-4 times/month vs never | 0.68 | [0.3, 1.56] | -0.92 | .357 |
| 2-3 times/week vs never | 1.03 | [0.45, 2.38] | 0.07 | .943 |
| 4+ times/week vs never | 1.16 | [0.49, 2.72] | 0.34 | .735 |
| Country |  |  |  |  |
| U.K. vs Australia | 1.62 | [1.26, 2.1] | 3.70 | **<.001** |
| U.S. vs Australia | 1.64 | [1.22, 2.21] | 3.29 | **.001** |
| Pandemic-related stress | 1.03 | [1.01, 1.05] | 2.88 | **.004** |

*Note.* Std = standard.

**Table S13**

*Results for associations between age, timepoint, country, and changes in alcohol consumption frequency and quantity during the pandemic relative to pre-pandemic levels*

| Variable | Odds | 95% CI | *t* | *p* |
| --- | --- | --- | --- | --- |
| Full model results (with time x age interaction) |  |  |  |  |
| *Quantity model* |  |  |  |  |
| Age | 2.82 | [1.9, 4.19] | 5.15 | **<.001** |
| Age^2^ | 1.55 | [1.14, 2.1] | 2.79 | **.005** |
| Age^3^ | 0.68 | [0.56, 0.82] | -3.98 | **<.001** |
| T2 (vs T1) | 0.95 | [0.65, 1.39] | -0.26 | .791 |
| T3 (vs T1) | 0.81 | [0.55, 1.2] | -1.06 | .290 |
| U.K. (vs Australia) | 1.94 | [1.26, 2.99] | 3.03 | **.002** |
| U.S. (vs Australia) | 1.97 | [1.21, 3.21] | 2.71 | **.007** |
| Pandemic-related stress | 1.04 | [1.01, 1.06] | 2.79 | **.005** |
| Age x T2 | 0.80 | [0.49, 1.3] | -0.91 | .365 |
| Age x T3 | 0.76 | [0.47, 1.22] | -1.14 | .256 |
| Age^2^ x T2 | 1.32 | [0.85, 2.03] | 1.24 | .214 |
| Age^2^ x T3 | 1.05 | [0.68, 1.62] | 0.22 | .826 |
| Age^3^ x T2 | 0.91 | [0.71, 1.16] | -0.78 | .435 |
| Age^3^ x T3 | 1.04 | [0.83, 1.3] | 0.34 | .733 |
| *Frequency model* |  |  |  |  |
| Age | 1.98 | [1.38, 2.83] | 3.72 | **<.001** |
| Age^2^ | 1.21 | [0.91, 1.6] | 1.29 | .196 |
| Age^3^ | 0.79 | [0.66, 0.94] | -2.66 | **.008** |
| T2 (vs T1) | 0.88 | [0.61, 1.26] | -0.72 | .474 |
| T3 (vs T1) | 0.67 | [0.46, 0.97] | -2.13 | **.033** |
| U.K. (vs Australia) | 2.06 | [1.39, 3.07] | 3.58 | **<.001** |
| U.S. (vs Australia) | 1.93 | [1.23, 3.02] | 2.87 | **.004** |
| Pandemic-related stress | 1.03 | [1, 1.05] | 2.31 | **.021** |
| Age x T2 | 0.93 | [0.59, 1.48] | -0.29 | .770 |
| Age x T3 | 1.02 | [0.65, 1.6] | 0.09 | .930 |
| Age^2^ x T2 | 1.30 | [0.85, 1.97] | 1.22 | .223 |
| Age^2^ x T3 | 1.20 | [0.79, 1.81] | 0.87 | .387 |
| Age^3^ x T2 | 0.88 | [0.7, 1.11] | -1.06 | .288 |
| Age^3^ x T3 | 0.92 | [0.75, 1.13] | -0.79 | .429 |
| Reduced model results (without time x age interaction) |  |  |  |  |
| *Quantity model* |  |  |  |  |
| Age | 2.56 | [1.79, 3.66] | 5.14 | **<.001** |
| Age^2^ | 1.63 | [1.24, 2.15] | 3.48 | **.001** |
| Age^3^ | 0.67 | [0.57, 0.8] | -4.48 | **<.001** |
| T2 (vs T1) | 1.04 | [0.81, 1.34] | 0.29 | .772 |
| T3 (vs T1) | 0.83 | [0.64, 1.07] | -1.42 | .156 |
| U.K. (vs Australia) | 1.94 | [1.26, 2.97] | 3.02 | **.003** |
| U.S. (vs Australia) | 1.99 | [1.22, 3.24] | 2.77 | **.006** |
| Pandemic-related stress | 1.04 | [1.01, 1.06] | 2.73 | **.006** |
| *Frequency model* |  |  |  |  |
| Age | 1.97 | [1.42, 2.72] | 4.10 | **<.001** |
| Age^2^ | 1.31 | [1.02, 1.69] | 2.12 | **.034** |
| Age^3^ | 0.76 | [0.65, 0.88] | -3.50 | **<.001** |
| T2 (vs T1) | 0.95 | [0.75, 1.21] | -0.40 | .686 |
| T3 (vs T1) | 0.73 | [0.57, 0.94] | -2.45 | **.014** |
| U.K. (vs Australia) | 2.06 | [1.39, 3.05] | 3.59 | **<.001** |
| U.S. (vs Australia) | 1.93 | [1.23, 3.02] | 2.89 | **.004** |
| Pandemic-related stress | 1.03 | [1, 1.05] | 2.29 | **.022** |

**Table S14**

*Results for associations between age, country, and changes in drinking behaviour during the pandemic relative to pre-pandemic*

| Variable | Odds | 95% CI | *t* | *p* | *p*FDR |
| --- | --- | --- | --- | --- | --- |
| Baseline models (pre-COVID to T1) |  |  |  |  |  |
| *Drinking earlier* |  |  |  |  |  |
| (Intercept) | 0.16 | [0.11, 0.23] | -10.38 | <.001 | **<.001** |
| Age | 0.81 | [0.7, 0.95] | -2.65 | .008 | **.013** |
| U.K. (vs Australia) | 1.78 | [1.21, 2.68] | 2.85 | .004 | **.009** |
| U.S. (vs Australia) | 2.02 | [1.3, 3.19] | 3.10 | .002 | **.005** |
| Pandemic-related stress | 1.29 | [1.11, 1.52] | 3.18 | .001 | **.004** |
| *Difficulty stopping drinking* |  |  |  |  |  |
| (Intercept) | 0.12 | [0.08, 0.18] | -10.85 | <.001 | **<.001** |
| Age | 1.04 | [0.84, 1.27] | 0.34 | .736 | .789 |
| U.K. (vs Australia) | 0.80 | [0.5, 1.29] | -0.93 | .354 | .458 |
| U.S. (vs Australia) | 0.77 | [0.44, 1.35] | -0.90 | .367 | .458 |
| Pandemic-related stress | 1.35 | [1.09, 1.67] | 2.75 | .006 | **.011** |
| *Drinking alone* |  |  |  |  |  |
| (Intercept) | 0.29 | [0.21, 0.38] | -8.59 | <.001 | **<.001** |
| Age | 0.92 | [0.8, 1.06] | -1.15 | .250 | .375 |
| U.K. (vs Australia) | 1.10 | [0.78, 1.56] | 0.53 | .597 | .689 |
| U.S. (vs Australia) | 1.05 | [0.7, 1.58] | 0.24 | .808 | .808 |
| Pandemic-related stress | 1.36 | [1.17, 1.58] | 3.95 | <.001 | **<.001** |
| Prospective models (T1-T3) |  |  |  |  |  |
| *Drinking earlier* |  |  |  |  |  |
| Age | 0.74 | [0.45, 1.21] | -1.20 | .229 | .558 |
| T2 (vs T1) | 0.39 | [0.19, 0.81] | -2.54 | .011 | .202 |
| T3 (vs T1) | 0.44 | [0.21, 0.92] | -2.19 | .028 | .255 |
| U.K. (vs Australia) | 1.94 | [0.56, 6.79] | 1.04 | .298 | .597 |
| U.S. (vs Australia) | 2.27 | [0.57, 9.1] | 1.15 | .248 | .558 |
| Pandemic-related stress | 1.55 | [1, 2.39] | 1.95 | .051 | .308 |
| *Difficulty stopping drinking* |  |  |  |  |  |
| Age | 0.89 | [0.58, 1.34] | -0.57 | .568 | .852 |
| T2 (vs T1) | 0.66 | [0.37, 1.18] | -1.39 | .164 | .558 |
| T3 (vs T1) | 1.26 | [0.72, 2.2] | 0.82 | .415 | .747 |
| U.K. (vs Australia) | 1.23 | [0.46, 3.29] | 0.40 | .686 | .882 |
| U.S. (vs Australia) | 1.06 | [0.34, 3.31] | 0.10 | .917 | .969 |
| Pandemic-related stress | 1.26 | [0.86, 1.84] | 1.19 | .234 | .558 |
| *Drinking alone* |  |  |  |  |  |
| Age | 0.97 | [0.55, 1.69] | -0.12 | .902 | .969 |
| T2 (vs T1) | 1.21 | [0.57, 2.6] | 0.49 | .621 | .860 |
| T3 (vs T1) | 1.27 | [0.58, 2.78] | 0.60 | .545 | .852 |
| U.K. (vs Australia) | 0.97 | [0.26, 3.71] | -0.04 | .969 | .969 |
| U.S. (vs Australia) | 0.85 | [0.18, 3.99] | -0.21 | .832 | .969 |
| Pandemic-related stress | 1.47 | [0.88, 2.45] | 1.49 | .137 | .558 |

*Note.* Results based on reduced model without age by wave interaction as full model did not yield significant interaction effects.

**Table S15**

*Results for associations between age, country, and changes in motivations for alcohol use during the pandemic relative to pre-pandemic*

| Variable | Odds | 95% CI | *t* | *p* | *p*FDR |
| --- | --- | --- | --- | --- | --- |
| Baseline models (pre-COVID to T1) |  |  |  |  |  |
| *Drinking to kill boredom* |  |  |  |  |  |
| (Intercept) | 0.24 | [0.17, 0.32] | -9.23 | <.001 | **<.001** |
| Age | 0.75 | [0.64, 0.87] | -3.71 | <.001 | **.001** |
| U.K. (vs Australia) | 1.18 | [0.82, 1.71] | 0.88 | .379 | .406 |
| U.S. (vs Australia) | 1.30 | [0.86, 1.99] | 1.24 | .217 | .255 |
| Pandemic-related stress | 1.36 | [1.16, 1.59] | 3.81 | <.001 | **<.001** |
| *Drinking to destress* |  |  |  |  |  |
| (Intercept) | 0.41 | [0.31, 0.53] | -6.58 | <.001 | **<.001** |
| Age | 0.88 | [0.77, 1.01] | -1.86 | .063 | .106 |
| U.K. (vs Australia) | 0.94 | [0.68, 1.31] | -0.36 | .721 | .721 |
| U.S. (vs Australia) | 1.26 | [0.87, 1.83] | 1.22 | .221 | .255 |
| Pandemic-related stress | 1.63 | [1.41, 1.89] | 6.52 | <.001 | **<.001** |
| *Drinking to enjoy* |  |  |  |  |  |
| (Intercept) | 0.48 | [0.37, 0.62] | -5.69 | <.001 | **<.001** |
| Age | 0.81 | [0.72, 0.92] | -3.19 | .001 | **.003** |
| U.K. (vs Australia) | 1.23 | [0.91, 1.68] | 1.33 | .182 | .248 |
| U.S. (vs Australia) | 1.35 | [0.94, 1.92] | 1.64 | .102 | .152 |
| Pandemic-related stress | 1.24 | [1.08, 1.41] | 3.16 | .002 | **.003** |
| Prospective models (T1-T3) |  |  |  |  |  |
| *Drinking to kill boredom* |  |  |  |  |  |
| Age | 0.69 | [0.43, 1.11] | -1.54 | .123 | .277 |
| T2 (vs T1) | 0.71 | [0.38, 1.31] | -1.10 | .272 | .408 |
| T3 (vs T1) | 0.61 | [0.32, 1.17] | -1.48 | .140 | .280 |
| U.K. (vs Australia) | 1.27 | [0.42, 3.83] | 0.43 | .666 | .749 |
| U.S. (vs Australia) | 1.42 | [0.41, 4.92] | 0.55 | .584 | .701 |
| Pandemic-related stress | 1.59 | [1.05, 2.39] | 2.22 | .027 | .080 |
| *Drinking to destress* |  |  |  |  |  |
| Age | 0.77 | [0.52, 1.14] | -1.32 | .186 | .335 |
| T2 (vs T1) | 0.64 | [0.36, 1.13] | -1.54 | .123 | .277 |
| T3 (vs T1) | 0.77 | [0.45, 1.33] | -0.92 | .356 | .493 |
| U.K. (vs Australia) | 1.17 | [0.45, 3.02] | 0.33 | .743 | .787 |
| U.S. (vs Australia) | 1.97 | [0.67, 5.83] | 1.22 | .221 | .362 |
| Pandemic-related stress | 1.65 | [1.14, 2.37] | 2.69 | .007 | .039 |
| *Drinking to enjoy* |  |  |  |  |  |
| Age | 0.70 | [0.6, 0.82] | -4.52 | <.001 | **<.001** |
| T2 (vs T1) | 0.97 | [0.72, 1.31] | -0.19 | .851 | .851 |
| T3 (vs T1) | 0.89 | [0.65, 1.21] | -0.74 | .458 | .589 |
| U.K. (vs Australia) | 1.57 | [1.08, 2.27] | 2.36 | .018 | .065 |
| U.S. (vs Australia) | 1.76 | [1.15, 2.7] | 2.62 | .009 | **.039** |
| Pandemic-related stress | 1.35 | [1.15, 1.58] | 3.77 | <.001 | **.001** |

*Note.* Results based on reduced model without age by wave interaction as full model did not yield significant interaction effects.

Cross-lagged models were not conducted with pandemic-related stress as an additional covariate because it was treated as a time-varying longitudinal construct rather than a time-invariant background characteristic and was examined as a focal variable as part of the main cross-lagged model analysis.

### Results for analyses without pregnant sample

**Table S16**

*Results for associations between pre-pandemic alcohol consumption quantity and frequency and relative changes in consumption at T1*

| Variable | Odds | 95% CI | *t* | *p* |
| --- | --- | --- | --- | --- |
| Quantity Model |  |  |  |  |
| Pre-pandemic quantity level |  |  |  |  |
| 3-4 vs 1-2 std drinks | 1.19 | [0.4, 3.58] | 0.31 | .753 |
| 5-6 vs 1-2 std drinks | 1.02 | [0.39, 2.71] | 0.05 | .963 |
| 7-9 vs 1-2 std drinks | 1.65 | [0.72, 3.77] | 1.18 | .236 |
| 10 or more vs 1-2 std drinks | 1.48 | [0.76, 2.87] | 1.15 | .249 |
| Country |  |  |  |  |
| U.K. vs Australia | 1.47 | [1.11, 1.95] | 2.70 | **.007** |
| U.S. vs Australia | 1.60 | [1.16, 2.21] | 2.88 | **.004** |
| Frequency Model |  |  |  |  |
| Pre-pandemic frequency level |  |  |  |  |
| Monthly or less vs never | 0.55 | [0.22, 1.4] | -1.26 | .206 |
| 2-4 times/month vs never | 0.55 | [0.22, 1.38] | -1.28 | .199 |
| 2-3 times/week vs never | 0.90 | [0.35, 2.29] | -0.22 | .822 |
| 4+ times/week vs never | 0.84 | [0.32, 2.17] | -0.37 | .713 |
| Country |  |  |  |  |
| U.K. vs Australia | 1.76 | [1.33, 2.31] | 4.01 | **<.001** |
| U.S. vs Australia | 1.71 | [1.26, 2.34] | 3.39 | **.001** |

*Note.* Std = standard.

**Table S17**

*Results for associations between age, timepoint, country, and changes in alcohol consumption frequency and quantity during the pandemic relative to pre-pandemic levels*

| Variable | Odds | 95% CI | *t* | *p* |
| --- | --- | --- | --- | --- |
| Full model results (with time x age interaction) |  |  |  |  |
| *Quantity model* |  |  |  |  |
| Age | 1.75 | [1.18, 2.58] | 2.81 | **.005** |
| Age^2^ | 0.86 | [0.63, 1.18] | -0.92 | .356 |
| Age^3^ | 0.91 | [0.75, 1.1] | -1.00 | .316 |
| T2 (vs T1) | 0.82 | [0.55, 1.23] | -0.96 | .336 |
| T3 (vs T1) | 0.57 | [0.37, 0.87] | -2.61 | **.009** |
| U.K. (vs Australia) | 2.06 | [1.31, 3.24] | 3.14 | **.002** |
| U.S. (vs Australia) | 2.03 | [1.22, 3.38] | 2.72 | **.007** |
| Age x T2 | 0.86 | [0.53, 1.42] | -0.58 | .562 |
| Age x T3 | 0.88 | [0.54, 1.43] | -0.51 | .608 |
| Age^2^ x T2 | 1.49 | [0.95, 2.34] | 1.75 | .080 |
| Age^2^ x T3 | 1.38 | [0.88, 2.17] | 1.41 | .157 |
| Age^3^ x T2 | 0.85 | [0.67, 1.09] | -1.26 | .209 |
| Age^3^ x T3 | 0.91 | [0.72, 1.15] | -0.79 | .429 |
| *Frequency model* |  |  |  |  |
| Age | 1.25 | [0.87, 1.79] | 1.20 | .230 |
| Age^2^ | 0.66 | [0.49, 0.89] | -2.71 | **.007** |
| Age^3^ | 1.06 | [0.88, 1.26] | 0.60 | .547 |
| T2 (vs T1) | 0.67 | [0.45, 1] | -1.96 | **.050** |
| T3 (vs T1) | 0.43 | [0.29, 0.65] | -3.98 | **<.001** |
| U.K. (vs Australia) | 2.32 | [1.52, 3.53] | 3.93 | **<.001** |
| U.S. (vs Australia) | 2.16 | [1.34, 3.49] | 3.16 | **.002** |
| Age x T2 | 1.08 | [0.67, 1.74] | 0.32 | .747 |
| Age x T3 | 1.19 | [0.75, 1.89] | 0.75 | .455 |
| Age^2^ x T2 | 1.55 | [1, 2.4] | 1.97 | **.049** |
| Age^2^ x T3 | 1.65 | [1.08, 2.54] | 2.30 | **.022** |
| Age^3^ x T2 | 0.81 | [0.64, 1.02] | -1.80 | .073 |
| Age^3^ x T3 | 0.79 | [0.64, 0.98] | -2.11 | **.035** |
| Reduced model results (without time x age interaction) |  |  |  |  |
| *Quantity model* |  |  |  |  |
| Age | 1.73 | [1.21, 2.46] | 3.04 | **.002** |
| Age^2^ | 1.00 | [0.75, 1.32] | -0.01 | .991 |
| Age^3^ | 0.86 | [0.73, 1.03] | -1.67 | .095 |
| T2 (vs T1) | 0.98 | [0.75, 1.27] | -0.19 | .852 |
| T3 (vs T1) | 0.70 | [0.53, 0.93] | -2.51 | **.012** |
| U.K. (vs Australia) | 2.01 | [1.28, 3.14] | 3.06 | **.002** |
| U.S. (vs Australia) | 1.95 | [1.17, 3.26] | 2.54 | **.011** |
| *Frequency model* |  |  |  |  |
| Age | 1.33 | [0.96, 1.84] | 1.69 | .091 |
| Age^2^ | 0.79 | [0.61, 1.03] | -1.73 | .083 |
| Age^3^ | 0.97 | [0.82, 1.13] | -0.43 | .664 |
| T2 (vs T1) | 0.84 | [0.65, 1.08] | -1.34 | .180 |
| T3 (vs T1) | 0.61 | [0.47, 0.79] | -3.72 | **<.001** |
| U.K. (vs Australia) | 2.31 | [1.52, 3.52] | 3.92 | **<.001** |
| U.S. (vs Australia) | 2.16 | [1.34, 3.47] | 3.18 | **.001** |

**Table S18**

*Results for associations between age, country, and changes in drinking behaviour during the pandemic relative to pre-pandemic*

| Variable | Odds | 95% CI | *t* | *p* | *p*FDR |
| --- | --- | --- | --- | --- | --- |
| Baseline models (pre-COVID to T1) |  |  |  |  |  |
| *Drinking earlier* |  |  |  |  |  |
| (Intercept) | 0.18 | [0.13, 0.26] | -9.67 | <.001 | **<.001** |
| Age | 0.72 | [0.61, 0.85] | -3.89 | <.001 | **<.001** |
| U.K. (vs Australia) | 1.73 | [1.17, 2.6] | 2.70 | .007 | **.012** |
| U.S. (vs Australia) | 2.05 | [1.32, 3.21] | 3.16 | .002 | **.004** |
| *Difficulty stopping drinking* |  |  |  |  |  |
| (Intercept) | 0.14 | [0.1, 0.21] | -10.22 | <.001 | **<.001** |
| Age | 0.93 | [0.75, 1.14] | -0.71 | .478 | .573 |
| U.K. (vs Australia) | 0.79 | [0.5, 1.27] | -1.00 | .318 | .477 |
| U.S. (vs Australia) | 0.79 | [0.45, 1.36] | -0.85 | .394 | .525 |
| *Drinking alone* |  |  |  |  |  |
| (Intercept) | 0.33 | [0.24, 0.43] | -7.62 | <.001 | **<.001** |
| Age | 0.81 | [0.7, 0.94] | -2.72 | .007 | **.012** |
| U.K. (vs Australia) | 1.11 | [0.78, 1.57] | 0.57 | .568 | .619 |
| U.S. (vs Australia) | 1.08 | [0.72, 1.61] | 0.36 | .715 | .715 |
| Prospective models (T1-T3) |  |  |  |  |  |
| *Drinking earlier* |  |  |  |  |  |
| Age | 0.66 | [0.42, 1.05] | -1.74 | .083 | .329 |
| T2 (vs T1) | 0.33 | [0.16, 0.7] | -2.91 | .004 | **.027** |
| T3 (vs T1) | 0.27 | [0.12, 0.6] | -3.21 | .001 | **.020** |
| U.K. (vs Australia) | 2.04 | [0.57, 7.29] | 1.10 | .271 | .581 |
| U.S. (vs Australia) | 2.64 | [0.65, 10.81] | 1.35 | .176 | .529 |
| *Difficulty stopping drinking* |  |  |  |  |  |
| Age | 0.78 | [0.52, 1.16] | -1.24 | .215 | .537 |
| T2 (vs T1) | 0.60 | [0.34, 1.08] | -1.71 | .088 | .329 |
| T3 (vs T1) | 1.09 | [0.62, 1.94] | 0.30 | .761 | .963 |
| U.K. (vs Australia) | 1.27 | [0.46, 3.53] | 0.47 | .641 | .961 |
| U.S. (vs Australia) | 1.16 | [0.36, 3.7] | 0.25 | .805 | .963 |
| *Drinking alone* |  |  |  |  |  |
| Age | 0.85 | [0.5, 1.46] | -0.58 | .564 | .961 |
| T2 (vs T1) | 1.20 | [0.56, 2.59] | 0.48 | .635 | .961 |
| T3 (vs T1) | 1.04 | [0.47, 2.33] | 0.10 | .924 | .963 |
| U.K. (vs Australia) | 0.97 | [0.25, 3.77] | -0.05 | .963 | .963 |
| U.S. (vs Australia) | 0.91 | [0.19, 4.33] | -0.12 | .901 | .963 |

*Note.* Results based on reduced model without age by wave interaction as full model did not yield significant interaction effects.

**Table S19**

*Results for associations between age, country, and changes in motivations for drinking during the pandemic relative to pre-pandemic*

| Variable | Odds | 95% CI | *t* | *p* | *p*FDR |
| --- | --- | --- | --- | --- | --- |
| Baseline models (pre-COVID to T1) |  |  |  |  |  |
| *Drinking to kill boredom* |  |  |  |  |  |
| (Intercept) | 0.27 | [0.2, 0.36] | -8.42 | <.001 | **<.001** |
| Age | 0.66 | [0.56, 0.77] | -5.07 | <.001 | **<.001** |
| U.K. (vs Australia) | 1.19 | [0.83, 1.73] | 0.92 | .355 | .388 |
| U.S. (vs Australia) | 1.37 | [0.9, 2.08] | 1.46 | .143 | .191 |
| *Drinking to destress* |  |  |  |  |  |
| (Intercept) | 0.46 | [0.35, 0.6] | -5.65 | <.001 | **<.001** |
| Age | 0.74 | [0.64, 0.85] | -4.20 | <.001 | **<.001** |
| U.K. (vs Australia) | 0.97 | [0.7, 1.35] | -0.16 | .870 | .870 |
| U.S. (vs Australia) | 1.36 | [0.94, 1.97] | 1.62 | .104 | .177 |
| *Drinking to enjoy* |  |  |  |  |  |
| (Intercept) | 0.57 | [0.44, 0.73] | -4.30 | <.001 | **<.001** |
| Age | 0.70 | [0.61, 0.81] | -5.04 | <.001 | **<.001** |
| U.K. (vs Australia) | 1.21 | [0.89, 1.66] | 1.19 | .234 | .281 |
| U.S. (vs Australia) | 1.33 | [0.93, 1.92] | 1.56 | .118 | .177 |
| Prospective models (T1-T3) |  |  |  |  |  |
| *Drinking to kill boredom* |  |  |  |  |  |
| Age | 0.60 | [0.38, 0.94] | -2.21 | .027 | .067 |
| T2 (vs T1) | 0.62 | [0.33, 1.17] | -1.46 | .144 | .196 |
| T3 (vs T1) | 0.49 | [0.25, 0.95] | -2.10 | .036 | .077 |
| U.K. (vs Australia) | 1.34 | [0.44, 4.11] | 0.51 | .610 | .610 |
| U.S. (vs Australia) | 1.62 | [0.45, 5.74] | 0.74 | .458 | .529 |
| *Drinking to destress* |  |  |  |  |  |
| Age | 0.65 | [0.44, 0.95] | -2.21 | .027 | .067 |
| T2 (vs T1) | 0.49 | [0.27, 0.87] | -2.42 | .016 | .067 |
| T3 (vs T1) | 0.59 | [0.34, 1.05] | -1.79 | .074 | .123 |
| U.K. (vs Australia) | 1.31 | [0.49, 3.55] | 0.54 | .590 | .610 |
| U.S. (vs Australia) | 2.35 | [0.75, 7.41] | 1.46 | .144 | .196 |
| *Drinking to enjoy* |  |  |  |  |  |
| Age | 0.60 | [0.51, 0.71] | -6.08 | <.001 | **<.001** |
| T2 (vs T1) | 0.81 | [0.59, 1.11] | -1.33 | .182 | .228 |
| T3 (vs T1) | 0.73 | [0.52, 1.01] | -1.89 | .058 | .110 |
| U.K. (vs Australia) | 1.62 | [1.08, 2.43] | 2.35 | .019 | .067 |
| U.S. (vs Australia) | 1.89 | [1.2, 2.98] | 2.74 | .006 | **.046** |

*Note.* Results based on reduced model without age by wave interaction as full model did not yield significant interaction effects.

**Table S20**

*Results for cross-lagged panel model examining associations between depression symptoms and alcohol consumption frequency and quantity*

| Predictor | Outcome | Path type | *b* | 95% CI | *p* |
| --- | --- | --- | --- | --- | --- |
| T1 Depression | T2 Depression | Regression | 0.79 | [0.69, 0.89] | **<.001** |
| T1 Alcohol Frequency | T2 Depression | Regression | -0.02 | [-1.06, 1.02] | .964 |
| T1 Alcohol Quantity | T2 Depression | Regression | 0.36 | [-0.63, 1.34] | .478 |
| T1 Depression | T2 Alcohol Frequency | Regression | 0.00 | [-0.01, 0.02] | .715 |
| T1 Alcohol Frequency | T2 Alcohol Frequency | Regression | 0.32 | [0.12, 0.53] | **.002** |
| T1 Alcohol Quantity | T2 Alcohol Frequency | Regression | 0.42 | [0.23, 0.62] | **<.001** |
| T1 Depression | T2 Alcohol Quantity | Regression | 0.01 | [-0.01, 0.03] | .204 |
| T1 Alcohol Frequency | T2 Alcohol Quantity | Regression | 0.31 | [0.13, 0.49] | **.001** |
| T1 Alcohol Quantity | T2 Alcohol Quantity | Regression | 0.38 | [0.2, 0.56] | **<.001** |
| T2 Depression | T3 Depression | Regression | 0.71 | [0.61, 0.8] | **<.001** |
| T2 Alcohol Frequency | T3 Depression | Regression | -0.22 | [-0.91, 0.47] | .533 |
| T2 Alcohol Quantity | T3 Depression | Regression | 0.35 | [-0.42, 1.13] | .371 |
| T2 Depression | T3 Alcohol Frequency | Regression | 0.00 | [-0.02, 0.01] | .623 |
| T2 Alcohol Frequency | T3 Alcohol Frequency | Regression | 0.43 | [0.21, 0.66] | **<.001** |
| T2 Alcohol Quantity | T3 Alcohol Frequency | Regression | 0.23 | [-0.01, 0.47] | .058 |
| T2 Depression | T3 Alcohol Quantity | Regression | 0.00 | [-0.02, 0.01] | .695 |
| T2 Alcohol Frequency | T3 Alcohol Quantity | Regression | 0.22 | [0.03, 0.4] | **.020** |
| T2 Alcohol Quantity | T3 Alcohol Quantity | Regression | 0.46 | [0.26, 0.66] | **<.001** |
| Age | T1 Depression | Regression | -0.08 | [-0.12, -0.04] | **<.001** |
| Pre-COVID Frequency | T1 Depression | Regression | -0.44 | [-1.18, 0.3] | .244 |
| Pre-COVID Quantity | T1 Depression | Regression | 1.20 | [-0.36, 2.77] | .132 |
| U.K. (vs Australia) | T1 Depression | Regression | 1.53 | [-0.15, 3.22] | .075 |
| U.S. (vs Australia) | T1 Depression | Regression | 1.39 | [-0.47, 3.25] | .142 |
| Age | T1 Alcohol Frequency | Regression | 0.00 | [-0.01, 0.01] | .572 |
| Pre-COVID Frequency | T1 Alcohol Frequency | Regression | 0.03 | [-0.11, 0.17] | .679 |
| Pre-COVID Quantity | T1 Alcohol Frequency | Regression | 0.10 | [-0.06, 0.27] | .205 |
| U.K. (vs Australia) | T1 Alcohol Frequency | Regression | 0.20 | [-0.15, 0.55] | .258 |
| U.S. (vs Australia) | T1 Alcohol Frequency | Regression | 0.41 | [0.05, 0.77] | **.025** |
| Age | T1 Alcohol Quantity | Regression | 0.00 | [0, 0.01] | .352 |
| Pre-COVID Frequency | T1 Alcohol Quantity | Regression | 0.12 | [-0.03, 0.27] | .120 |
| Pre-COVID Quantity | T1 Alcohol Quantity | Regression | -0.03 | [-0.22, 0.16] | .744 |
| U.K. (vs Australia) | T1 Alcohol Quantity | Regression | 0.20 | [-0.17, 0.56] | .287 |
| U.S. (vs Australia) | T1 Alcohol Quantity | Regression | 0.41 | [0.03, 0.78] | **.033** |
| Age | T2 Depression | Regression | -0.01 | [-0.04, 0.02] | .479 |
| Pre-COVID Frequency | T2 Depression | Regression | -0.13 | [-0.62, 0.36] | .603 |
| Pre-COVID Quantity | T2 Depression | Regression | 0.11 | [-0.66, 0.88] | .779 |
| U.K. (vs Australia) | T2 Depression | Regression | -0.78 | [-2.1, 0.53] | .242 |
| U.S. (vs Australia) | T2 Depression | Regression | 0.02 | [-1.35, 1.38] | .980 |
| Age | T2 Alcohol Frequency | Regression | 0.00 | [-0.01, 0] | .194 |
| Pre-COVID Frequency | T2 Alcohol Frequency | Regression | -0.04 | [-0.15, 0.07] | .485 |
| Pre-COVID Quantity | T2 Alcohol Frequency | Regression | 0.12 | [-0.07, 0.31] | .209 |
| U.K. (vs Australia) | T2 Alcohol Frequency | Regression | 0.13 | [-0.16, 0.43] | .366 |
| U.S. (vs Australia) | T2 Alcohol Frequency | Regression | -0.01 | [-0.3, 0.29] | .956 |
| Age | T2 Alcohol Quantity | Regression | 0.00 | [-0.01, 0] | .197 |
| Pre-COVID Frequency | T2 Alcohol Quantity | Regression | -0.02 | [-0.13, 0.09] | .753 |
| Pre-COVID Quantity | T2 Alcohol Quantity | Regression | 0.09 | [-0.14, 0.31] | .448 |
| U.K. (vs Australia) | T2 Alcohol Quantity | Regression | -0.14 | [-0.41, 0.14] | .338 |
| U.S. (vs Australia) | T2 Alcohol Quantity | Regression | -0.04 | [-0.32, 0.24] | .798 |
| Age | T3 Depression | Regression | -0.03 | [-0.06, 0] | **.043** |
| Pre-COVID Frequency | T3 Depression | Regression | 0.12 | [-0.41, 0.66] | .657 |
| Pre-COVID Quantity | T3 Depression | Regression | 0.31 | [-0.42, 1.04] | .410 |
| U.K. (vs Australia) | T3 Depression | Regression | 0.63 | [-0.5, 1.75] | .276 |
| U.S. (vs Australia) | T3 Depression | Regression | 0.60 | [-0.48, 1.67] | .277 |
| Age | T3 Alcohol Frequency | Regression | 0.00 | [0, 0.01] | .269 |
| Pre-COVID Frequency | T3 Alcohol Frequency | Regression | 0.05 | [-0.07, 0.17] | .423 |
| Pre-COVID Quantity | T3 Alcohol Frequency | Regression | -0.04 | [-0.22, 0.15] | .716 |
| U.K. (vs Australia) | T3 Alcohol Frequency | Regression | 0.04 | [-0.27, 0.35] | .806 |
| U.S. (vs Australia) | T3 Alcohol Frequency | Regression | 0.19 | [-0.11, 0.49] | .221 |
| Age | T3 Alcohol Quantity | Regression | 0.00 | [0, 0.01] | .342 |
| Pre-COVID Frequency | T3 Alcohol Quantity | Regression | 0.02 | [-0.1, 0.15] | .696 |
| Pre-COVID Quantity | T3 Alcohol Quantity | Regression | 0.08 | [-0.1, 0.26] | .399 |
| U.K. (vs Australia) | T3 Alcohol Quantity | Regression | 0.11 | [-0.2, 0.41] | .490 |
| U.S. (vs Australia) | T3 Alcohol Quantity | Regression | 0.04 | [-0.25, 0.33] | .779 |
| T1 Alcohol Frequency | T1 Depression | Covariance | 0.36 | [-0.32, 1.04] | .305 |
| T1 Alcohol Quantity | T1 Alcohol Frequency | Covariance | 0.89 | [0.72, 1.06] | **<.001** |
| T1 Alcohol Quantity | T1 Depression | Covariance | 0.46 | [-0.26, 1.18] | .211 |
| T2 Alcohol Frequency | T2 Depression | Covariance | 0.25 | [-0.23, 0.73] | .302 |
| T2 Alcohol Quantity | T2 Alcohol Frequency | Covariance | 0.41 | [0.29, 0.53] | **<.001** |
| T2 Alcohol Quantity | T2 Depression | Covariance | 0.26 | [-0.21, 0.72] | .283 |
| T3 Alcohol Frequency | T3 Depression | Covariance | 0.25 | [-0.15, 0.66] | .220 |
| T3 Alcohol Quantity | T3 Alcohol Frequency | Covariance | 0.53 | [0.37, 0.68] | **<.001** |
| T3 Alcohol Quantity | T3 Depression | Covariance | 0.37 | [-0.04, 0.79] | .080 |
| T1 Depression | T1 Depression | Variance | 28.66 | [23.42, 33.9] | **<.001** |
| T1 Alcohol Frequency | T1 Alcohol Frequency | Variance | 1.03 | [0.85, 1.22] | **<.001** |
| T1 Alcohol Quantity | T1 Alcohol Quantity | Variance | 1.03 | [0.85, 1.2] | **<.001** |
| T2 Depression | T2 Depression | Variance | 14.79 | [11.39, 18.2] | **<.001** |
| T2 Alcohol Frequency | T2 Alcohol Frequency | Variance | 0.61 | [0.47, 0.75] | **<.001** |
| T2 Alcohol Quantity | T2 Alcohol Quantity | Variance | 0.56 | [0.43, 0.68] | **<.001** |
| T3 Depression | T3 Depression | Variance | 11.71 | [8.3, 15.12] | **<.001** |
| T3 Alcohol Frequency | T3 Alcohol Frequency | Variance | 0.71 | [0.56, 0.87] | **<.001** |
| T3 Alcohol Quantity | T3 Alcohol Quantity | Variance | 0.64 | [0.47, 0.8] | **<.001** |

Model fit: *χ*^2^ (9) = 68.51, *p* < .001, CFI = 0.96, TFI = 0.67, RMSEA = 0.17, SRMR = 0.04

**Table S21**

*Results for cross-lagged panel model examining associations between anxiety symptoms and alcohol consumption frequency and quantity*

| Predictor | Outcome | Path type | *b* | 95% CI | *p* |
| --- | --- | --- | --- | --- | --- |
| T1 Anxiety | T2 Anxiety | Regression | 0.71 | [0.6, 0.82] | **<.001** |
| T1 Alcohol Frequency | T2 Anxiety | Regression | -0.09 | [-0.92, 0.74] | .829 |
| T1 Alcohol Quantity | T2 Anxiety | Regression | 0.44 | [-0.35, 1.23] | .272 |
| T1 Anxiety | T2 Alcohol Frequency | Regression | 0.00 | [-0.02, 0.01] | .624 |
| T1 Alcohol Frequency | T2 Alcohol Frequency | Regression | 0.27 | [0.05, 0.49] | **.018** |
| T1 Alcohol Quantity | T2 Alcohol Frequency | Regression | 0.49 | [0.28, 0.7] | **<.001** |
| T1 Anxiety | T2 Alcohol Quantity | Regression | 0.00 | [-0.01, 0.02] | .681 |
| T1 Alcohol Frequency | T2 Alcohol Quantity | Regression | 0.25 | [0.05, 0.45] | **.014** |
| T1 Alcohol Quantity | T2 Alcohol Quantity | Regression | 0.45 | [0.25, 0.65] | **<.001** |
| T2 Anxiety | T3 Anxiety | Regression | 0.72 | [0.62, 0.82] | **<.001** |
| T2 Alcohol Frequency | T3 Anxiety | Regression | 0.07 | [-0.64, 0.79] | .840 |
| T2 Alcohol Quantity | T3 Anxiety | Regression | 0.28 | [-0.46, 1.02] | .452 |
| T2 Anxiety | T3 Alcohol Frequency | Regression | 0.00 | [-0.02, 0.03] | .662 |
| T2 Alcohol Frequency | T3 Alcohol Frequency | Regression | 0.44 | [0.22, 0.66] | **<.001** |
| T2 Alcohol Quantity | T3 Alcohol Frequency | Regression | 0.22 | [-0.02, 0.46] | .070 |
| T2 Anxiety | T3 Alcohol Quantity | Regression | 0.01 | [-0.01, 0.03] | .521 |
| T2 Alcohol Frequency | T3 Alcohol Quantity | Regression | 0.22 | [0.04, 0.4] | **.018** |
| T2 Alcohol Quantity | T3 Alcohol Quantity | Regression | 0.45 | [0.25, 0.65] | **<.001** |
| Age | T1 Anxiety | Regression | -0.09 | [-0.12, -0.05] | **<.001** |
| Pre-COVID Frequency | T1 Anxiety | Regression | -0.13 | [-0.77, 0.51] | .696 |
| Pre-COVID Quantity | T1 Anxiety | Regression | 1.13 | [-0.25, 2.51] | .110 |
| U.K. (vs Australia) | T1 Anxiety | Regression | 1.34 | [-0.32, 2.99] | .114 |
| U.S. (vs Australia) | T1 Anxiety | Regression | 2.01 | [0.22, 3.8] | **.028** |
| Age | T1 Alcohol Frequency | Regression | 0.00 | [-0.01, 0.01] | .543 |
| Pre-COVID Frequency | T1 Alcohol Frequency | Regression | 0.04 | [-0.1, 0.18] | .569 |
| Pre-COVID Quantity | T1 Alcohol Frequency | Regression | 0.11 | [-0.05, 0.28] | .174 |
| U.K. (vs Australia) | T1 Alcohol Frequency | Regression | 0.19 | [-0.16, 0.54] | .281 |
| U.S. (vs Australia) | T1 Alcohol Frequency | Regression | 0.43 | [0.07, 0.79] | **.019** |
| Age | T1 Alcohol Quantity | Regression | 0.00 | [0, 0.01] | .379 |
| Pre-COVID Frequency | T1 Alcohol Quantity | Regression | 0.11 | [-0.04, 0.27] | .141 |
| Pre-COVID Quantity | T1 Alcohol Quantity | Regression | -0.04 | [-0.23, 0.15] | .691 |
| U.K. (vs Australia) | T1 Alcohol Quantity | Regression | 0.19 | [-0.17, 0.56] | .296 |
| U.S. (vs Australia) | T1 Alcohol Quantity | Regression | 0.38 | [0.01, 0.76] | **.044** |
| Age | T2 Anxiety | Regression | -0.02 | [-0.05, 0] | .098 |
| Pre-COVID Frequency | T2 Anxiety | Regression | -0.28 | [-0.72, 0.16] | .211 |
| Pre-COVID Quantity | T2 Anxiety | Regression | -0.65 | [-1.27, -0.04] | **.037** |
| U.K. (vs Australia) | T2 Anxiety | Regression | 0.70 | [-0.44, 1.84] | .229 |
| U.S. (vs Australia) | T2 Anxiety | Regression | -0.23 | [-1.41, 0.95] | .704 |
| Age | T2 Alcohol Frequency | Regression | -0.01 | [-0.01, 0] | .110 |
| Pre-COVID Frequency | T2 Alcohol Frequency | Regression | -0.05 | [-0.16, 0.06] | .361 |
| Pre-COVID Quantity | T2 Alcohol Frequency | Regression | 0.13 | [-0.06, 0.33] | .187 |
| U.K. (vs Australia) | T2 Alcohol Frequency | Regression | 0.14 | [-0.15, 0.43] | .333 |
| U.S. (vs Australia) | T2 Alcohol Frequency | Regression | -0.02 | [-0.32, 0.28] | .877 |
| Age | T2 Alcohol Quantity | Regression | 0.00 | [-0.01, 0] | .105 |
| Pre-COVID Frequency | T2 Alcohol Quantity | Regression | -0.03 | [-0.14, 0.08] | .557 |
| Pre-COVID Quantity | T2 Alcohol Quantity | Regression | 0.10 | [-0.14, 0.33] | .408 |
| U.K. (vs Australia) | T2 Alcohol Quantity | Regression | -0.13 | [-0.41, 0.14] | .347 |
| U.S. (vs Australia) | T2 Alcohol Quantity | Regression | -0.06 | [-0.34, 0.23] | .687 |
| Age | T3 Anxiety | Regression | -0.02 | [-0.04, 0.01] | .188 |
| Pre-COVID Frequency | T3 Anxiety | Regression | 0.12 | [-0.33, 0.57] | .594 |
| Pre-COVID Quantity | T3 Anxiety | Regression | 0.32 | [-0.41, 1.05] | .394 |
| U.K. (vs Australia) | T3 Anxiety | Regression | 0.54 | [-0.46, 1.54] | .291 |
| U.S. (vs Australia) | T3 Anxiety | Regression | 1.09 | [-0.03, 2.22] | .057 |
| Age | T3 Alcohol Frequency | Regression | 0.00 | [0, 0.01] | .210 |
| Pre-COVID Frequency | T3 Alcohol Frequency | Regression | 0.05 | [-0.07, 0.18] | .400 |
| Pre-COVID Quantity | T3 Alcohol Frequency | Regression | -0.05 | [-0.24, 0.15] | .641 |
| U.K. (vs Australia) | T3 Alcohol Frequency | Regression | 0.02 | [-0.29, 0.34] | .896 |
| U.S. (vs Australia) | T3 Alcohol Frequency | Regression | 0.16 | [-0.13, 0.46] | .282 |
| Age | T3 Alcohol Quantity | Regression | 0.00 | [0, 0.01] | .256 |
| Pre-COVID Frequency | T3 Alcohol Quantity | Regression | 0.03 | [-0.09, 0.15] | .650 |
| Pre-COVID Quantity | T3 Alcohol Quantity | Regression | 0.07 | [-0.11, 0.26] | .436 |
| U.K. (vs Australia) | T3 Alcohol Quantity | Regression | 0.09 | [-0.21, 0.39] | .567 |
| U.S. (vs Australia) | T3 Alcohol Quantity | Regression | 0.02 | [-0.27, 0.3] | .893 |
| T1 Alcohol Frequency | T1 Anxiety | Covariance | 0.31 | [-0.41, 1.02] | .398 |
| T1 Alcohol Quantity | T1 Alcohol Frequency | Covariance | 0.89 | [0.72, 1.07] | **<.001** |
| T1 Alcohol Quantity | T1 Anxiety | Covariance | 0.37 | [-0.36, 1.09] | .319 |
| T2 Alcohol Frequency | T2 Anxiety | Covariance | 0.34 | [-0.08, 0.77] | .113 |
| T2 Alcohol Quantity | T2 Alcohol Frequency | Covariance | 0.43 | [0.3, 0.55] | **<.001** |
| T2 Alcohol Quantity | T2 Anxiety | Covariance | 0.32 | [-0.05, 0.7] | .090 |
| T3 Alcohol Frequency | T3 Anxiety | Covariance | 0.23 | [-0.14, 0.59] | .222 |
| T3 Alcohol Quantity | T3 Alcohol Frequency | Covariance | 0.53 | [0.38, 0.69] | **<.001** |
| T3 Alcohol Quantity | T3 Anxiety | Covariance | 0.35 | [0.01, 0.69] | **.045** |
| T1 Anxiety | T1 Anxiety | Variance | 26.20 | [21.01, 31.38] | **<.001** |
| T1 Alcohol Frequency | T1 Alcohol Frequency | Variance | 1.04 | [0.85, 1.22] | **<.001** |
| T1 Alcohol Quantity | T1 Alcohol Quantity | Variance | 1.04 | [0.87, 1.22] | **<.001** |
| T2 Anxiety | T2 Anxiety | Variance | 12.36 | [9.53, 15.19] | **<.001** |
| T2 Alcohol Frequency | T2 Alcohol Frequency | Variance | 0.62 | [0.48, 0.77] | **<.001** |
| T2 Alcohol Quantity | T2 Alcohol Quantity | Variance | 0.58 | [0.45, 0.7] | **<.001** |
| T3 Anxiety | T3 Anxiety | Variance | 10.51 | [7.89, 13.13] | **<.001** |
| T3 Alcohol Frequency | T3 Alcohol Frequency | Variance | 0.72 | [0.56, 0.88] | **<.001** |
| T3 Alcohol Quantity | T3 Alcohol Quantity | Variance | 0.64 | [0.48, 0.81] | **<.001** |

Model fit: *χ*^2^ (9) = 68.28, *p* < .001, CFI = 0.96, TFI = 0.67, RMSEA = 0.17, SRMR = 0.04

**Table S22**

*Results for cross-lagged panel model examining associations between pandemic-related stress and alcohol consumption frequency and quantity*

| Predictor | Outcome | Path type | *b* | 95% CI | *p* |
| --- | --- | --- | --- | --- | --- |
| T1 Pandemic Stress | T2 Pandemic Stress | Regression | 0.79 | [0.7, 0.89] | **<.001** |
| T1 Alcohol Frequency | T2 Pandemic Stress | Regression | 0.07 | [-0.88, 1.02] | .880 |
| T1 Alcohol Quantity | T2 Pandemic Stress | Regression | -0.13 | [-1.11, 0.86] | .797 |
| T1 Pandemic Stress | T2 Alcohol Frequency | Regression | -0.01 | [-0.03, 0] | .123 |
| T1 Alcohol Frequency | T2 Alcohol Frequency | Regression | 0.27 | [0.05, 0.49] | **.016** |
| T1 Alcohol Quantity | T2 Alcohol Frequency | Regression | 0.48 | [0.27, 0.7] | **<.001** |
| T1 Pandemic Stress | T2 Alcohol Quantity | Regression | -0.01 | [-0.02, 0.01] | .498 |
| T1 Alcohol Frequency | T2 Alcohol Quantity | Regression | 0.26 | [0.07, 0.46] | **.009** |
| T1 Alcohol Quantity | T2 Alcohol Quantity | Regression | 0.43 | [0.23, 0.63] | **<.001** |
| T2 Pandemic Stress | T3 Pandemic Stress | Regression | 0.75 | [0.67, 0.83] | **<.001** |
| T2 Alcohol Frequency | T3 Pandemic Stress | Regression | 0.31 | [-0.47, 1.1] | .437 |
| T2 Alcohol Quantity | T3 Pandemic Stress | Regression | -0.34 | [-1.21, 0.53] | .448 |
| T2 Pandemic Stress | T3 Alcohol Frequency | Regression | 0.00 | [-0.02, 0.01] | .727 |
| T2 Alcohol Frequency | T3 Alcohol Frequency | Regression | 0.44 | [0.21, 0.66] | **<.001** |
| T2 Alcohol Quantity | T3 Alcohol Frequency | Regression | 0.24 | [0, 0.47] | .053 |
| T2 Pandemic Stress | T3 Alcohol Quantity | Regression | 0.00 | [-0.02, 0.01] | .670 |
| T2 Alcohol Frequency | T3 Alcohol Quantity | Regression | 0.22 | [0.04, 0.4] | **.019** |
| T2 Alcohol Quantity | T3 Alcohol Quantity | Regression | 0.46 | [0.26, 0.66] | **<.001** |
| Age | T1 Pandemic Stress | Regression | -0.08 | [-0.12, -0.04] | **<.001** |
| Pre-COVID Frequency | T1 Pandemic Stress | Regression | -0.36 | [-1.14, 0.42] | .364 |
| Pre-COVID Quantity | T1 Pandemic Stress | Regression | 0.57 | [-0.68, 1.81] | .371 |
| U.K. (vs Australia) | T1 Pandemic Stress | Regression | 1.43 | [-0.62, 3.48] | .173 |
| U.S. (vs Australia) | T1 Pandemic Stress | Regression | 2.81 | [0.69, 4.93] | **.009** |
| Age | T1 Alcohol Frequency | Regression | 0.00 | [-0.01, 0.01] | .546 |
| Pre-COVID Frequency | T1 Alcohol Frequency | Regression | 0.04 | [-0.11, 0.18] | .616 |
| Pre-COVID Quantity | T1 Alcohol Frequency | Regression | 0.12 | [-0.05, 0.28] | .161 |
| U.K. (vs Australia) | T1 Alcohol Frequency | Regression | 0.16 | [-0.19, 0.51] | .370 |
| U.S. (vs Australia) | T1 Alcohol Frequency | Regression | 0.43 | [0.07, 0.79] | **.019** |
| Age | T1 Alcohol Quantity | Regression | 0.00 | [0, 0.01] | .395 |
| Pre-COVID Frequency | T1 Alcohol Quantity | Regression | 0.12 | [-0.04, 0.27] | .135 |
| Pre-COVID Quantity | T1 Alcohol Quantity | Regression | -0.04 | [-0.23, 0.15] | .710 |
| U.K. (vs Australia) | T1 Alcohol Quantity | Regression | 0.15 | [-0.21, 0.51] | .422 |
| U.S. (vs Australia) | T1 Alcohol Quantity | Regression | 0.39 | [0.01, 0.76] | **.043** |
| Age | T2 Pandemic Stress | Regression | -0.01 | [-0.05, 0.02] | .466 |
| Pre-COVID Frequency | T2 Pandemic Stress | Regression | -0.04 | [-0.63, 0.55] | .894 |
| Pre-COVID Quantity | T2 Pandemic Stress | Regression | -0.23 | [-1.09, 0.64] | .603 |
| U.K. (vs Australia) | T2 Pandemic Stress | Regression | -0.40 | [-1.93, 1.13] | .604 |
| U.S. (vs Australia) | T2 Pandemic Stress | Regression | -0.80 | [-2.35, 0.76] | .315 |
| Age | T2 Alcohol Frequency | Regression | -0.01 | [-0.01, 0] | .083 |
| Pre-COVID Frequency | T2 Alcohol Frequency | Regression | -0.06 | [-0.17, 0.05] | .277 |
| Pre-COVID Quantity | T2 Alcohol Frequency | Regression | 0.13 | [-0.06, 0.32] | .176 |
| U.K. (vs Australia) | T2 Alcohol Frequency | Regression | 0.18 | [-0.11, 0.47] | .230 |
| U.S. (vs Australia) | T2 Alcohol Frequency | Regression | 0.00 | [-0.31, 0.3] | .990 |
| Age | T2 Alcohol Quantity | Regression | -0.01 | [-0.01, 0] | .083 |
| Pre-COVID Frequency | T2 Alcohol Quantity | Regression | -0.04 | [-0.15, 0.07] | .489 |
| Pre-COVID Quantity | T2 Alcohol Quantity | Regression | 0.10 | [-0.12, 0.33] | .374 |
| U.K. (vs Australia) | T2 Alcohol Quantity | Regression | -0.09 | [-0.37, 0.19] | .531 |
| U.S. (vs Australia) | T2 Alcohol Quantity | Regression | -0.04 | [-0.33, 0.25] | .783 |
| Age | T3 Pandemic Stress | Regression | -0.03 | [-0.06, -0.01] | **.015** |
| Pre-COVID Frequency | T3 Pandemic Stress | Regression | -0.02 | [-0.49, 0.44] | .921 |
| Pre-COVID Quantity | T3 Pandemic Stress | Regression | 0.29 | [-0.47, 1.05] | .459 |
| U.K. (vs Australia) | T3 Pandemic Stress | Regression | 1.40 | [0.17, 2.63] | **.026** |
| U.S. (vs Australia) | T3 Pandemic Stress | Regression | 2.35 | [1.06, 3.64] | **<.001** |
| Age | T3 Alcohol Frequency | Regression | 0.00 | [0, 0.01] | .247 |
| Pre-COVID Frequency | T3 Alcohol Frequency | Regression | 0.05 | [-0.07, 0.17] | .440 |
| Pre-COVID Quantity | T3 Alcohol Frequency | Regression | -0.03 | [-0.22, 0.15] | .720 |
| U.K. (vs Australia) | T3 Alcohol Frequency | Regression | 0.03 | [-0.28, 0.35] | .829 |
| U.S. (vs Australia) | T3 Alcohol Frequency | Regression | 0.17 | [-0.13, 0.47] | .261 |
| Age | T3 Alcohol Quantity | Regression | 0.00 | [0, 0.01] | .367 |
| Pre-COVID Frequency | T3 Alcohol Quantity | Regression | 0.02 | [-0.1, 0.15] | .722 |
| Pre-COVID Quantity | T3 Alcohol Quantity | Regression | 0.07 | [-0.11, 0.25] | .430 |
| U.K. (vs Australia) | T3 Alcohol Quantity | Regression | 0.10 | [-0.19, 0.4] | .493 |
| U.S. (vs Australia) | T3 Alcohol Quantity | Regression | 0.03 | [-0.26, 0.31] | .852 |
| T1 Alcohol Frequency | T1 Pandemic Stress | Covariance | 0.40 | [-0.32, 1.13] | .278 |
| T1 Alcohol Quantity | T1 Alcohol Frequency | Covariance | 0.90 | [0.73, 1.07] | **<.001** |
| T1 Alcohol Quantity | T1 Pandemic Stress | Covariance | 0.75 | [-0.01, 1.5] | .052 |
| T2 Alcohol Frequency | T2 Pandemic Stress | Covariance | 0.15 | [-0.35, 0.65] | .562 |
| T2 Alcohol Quantity | T2 Alcohol Frequency | Covariance | 0.42 | [0.3, 0.54] | **<.001** |
| T2 Alcohol Quantity | T2 Pandemic Stress | Covariance | 0.20 | [-0.28, 0.69] | .405 |
| T3 Alcohol Frequency | T3 Pandemic Stress | Covariance | -0.01 | [-0.36, 0.34] | .948 |
| T3 Alcohol Quantity | T3 Alcohol Frequency | Covariance | 0.53 | [0.37, 0.68] | **<.001** |
| T3 Alcohol Quantity | T3 Pandemic Stress | Covariance | -0.02 | [-0.34, 0.31] | .906 |
| T1 Pandemic Stress | T1 Pandemic Stress | Variance | 31.08 | [25.29, 36.88] | **<.001** |
| T1 Alcohol Frequency | T1 Alcohol Frequency | Variance | 1.05 | [0.87, 1.23] | **<.001** |
| T1 Alcohol Quantity | T1 Alcohol Quantity | Variance | 1.06 | [0.88, 1.23] | **<.001** |
| T2 Pandemic Stress | T2 Pandemic Stress | Variance | 16.26 | [12.63, 19.88] | **<.001** |
| T2 Alcohol Frequency | T2 Alcohol Frequency | Variance | 0.62 | [0.47, 0.76] | **<.001** |
| T2 Alcohol Quantity | T2 Alcohol Quantity | Variance | 0.58 | [0.45, 0.7] | **<.001** |
| T3 Pandemic Stress | T3 Pandemic Stress | Variance | 12.40 | [9.71, 15.09] | **<.001** |
| T3 Alcohol Frequency | T3 Alcohol Frequency | Variance | 0.72 | [0.57, 0.88] | **<.001** |
| T3 Alcohol Quantity | T3 Alcohol Quantity | Variance | 0.63 | [0.47, 0.8] | **<.001** |

Model fit: *χ*^2^ (9) = 52.59, *p* < .001, CFI = 0.97, TFI = 0.76, RMSEA = 0.15, SRMR = 0.04

**Table S23**

*Results for cross-lagged panel model examining associations between general stress and alcohol consumption frequency and quantity*

| Predictor | Outcome | Path type | *b* | 95% CI | *p* |
| --- | --- | --- | --- | --- | --- |
| T1 General Stress | T2 General Stress | Regression | 0.67 | [0.55, 0.78] | **<.001** |
| T1 Alcohol Frequency | T2 General Stress | Regression | 0.68 | [0.09, 1.26] | .023 |
| T1 Alcohol Quantity | T2 General Stress | Regression | -0.32 | [-0.92, 0.27] | .289 |
| T1 General Stress | T2 Alcohol Frequency | Regression | 0.01 | [-0.02, 0.04] | .612 |
| T1 Alcohol Frequency | T2 Alcohol Frequency | Regression | 0.28 | [0.06, 0.5] | **.012** |
| T1 Alcohol Quantity | T2 Alcohol Frequency | Regression | 0.46 | [0.24, 0.68] | **<.001** |
| T1 General Stress | T2 Alcohol Quantity | Regression | 0.00 | [-0.04, 0.03] | .923 |
| T1 Alcohol Frequency | T2 Alcohol Quantity | Regression | 0.27 | [0.07, 0.47] | **.008** |
| T1 Alcohol Quantity | T2 Alcohol Quantity | Regression | 0.42 | [0.22, 0.63] | **<.001** |
| T2 General Stress | T3 General Stress | Regression | 0.50 | [0.4, 0.61] | **<.001** |
| T2 Alcohol Frequency | T3 General Stress | Regression | -0.08 | [-0.63, 0.47] | .783 |
| T2 Alcohol Quantity | T3 General Stress | Regression | 0.22 | [-0.36, 0.8] | .454 |
| T2 General Stress | T3 Alcohol Frequency | Regression | 0.01 | [-0.02, 0.04] | .439 |
| T2 Alcohol Frequency | T3 Alcohol Frequency | Regression | 0.43 | [0.21, 0.65] | **<.001** |
| T2 Alcohol Quantity | T3 Alcohol Frequency | Regression | 0.23 | [-0.01, 0.47] | .057 |
| T2 General Stress | T3 Alcohol Quantity | Regression | 0.01 | [-0.02, 0.04] | .478 |
| T2 Alcohol Frequency | T3 Alcohol Quantity | Regression | 0.22 | [0.04, 0.4] | **.019** |
| T2 Alcohol Quantity | T3 Alcohol Quantity | Regression | 0.45 | [0.25, 0.65] | **<.001** |
| Age | T1 General Stress | Regression | -0.05 | [-0.07, -0.02] | **<.001** |
| Pre-COVID Frequency | T1 General Stress | Regression | -0.07 | [-0.51, 0.38] | .763 |
| Pre-COVID Quantity | T1 General Stress | Regression | 0.45 | [-0.36, 1.26] | .276 |
| U.K. (vs Australia) | T1 General Stress | Regression | 1.38 | [0.36, 2.41] | **.008** |
| U.S. (vs Australia) | T1 General Stress | Regression | 0.93 | [-0.22, 2.08] | .112 |
| Age | T1 Alcohol Frequency | Regression | 0.00 | [-0.01, 0.01] | .508 |
| Pre-COVID Frequency | T1 Alcohol Frequency | Regression | 0.03 | [-0.11, 0.17] | .642 |
| Pre-COVID Quantity | T1 Alcohol Frequency | Regression | 0.11 | [-0.05, 0.27] | .176 |
| U.K. (vs Australia) | T1 Alcohol Frequency | Regression | 0.17 | [-0.18, 0.52] | .339 |
| U.S. (vs Australia) | T1 Alcohol Frequency | Regression | 0.43 | [0.07, 0.78] | **.020** |
| Age | T1 Alcohol Quantity | Regression | 0.00 | [0, 0.01] | .351 |
| Pre-COVID Frequency | T1 Alcohol Quantity | Regression | 0.11 | [-0.04, 0.26] | .146 |
| Pre-COVID Quantity | T1 Alcohol Quantity | Regression | -0.04 | [-0.23, 0.15] | .673 |
| U.K. (vs Australia) | T1 Alcohol Quantity | Regression | 0.16 | [-0.2, 0.52] | .387 |
| U.S. (vs Australia) | T1 Alcohol Quantity | Regression | 0.38 | [0.01, 0.75] | **.044** |
| Age | T2 General Stress | Regression | 0.00 | [-0.02, 0.02] | .776 |
| Pre-COVID Frequency | T2 General Stress | Regression | -0.11 | [-0.46, 0.24] | .533 |
| Pre-COVID Quantity | T2 General Stress | Regression | -0.05 | [-0.73, 0.63] | .887 |
| U.K. (vs Australia) | T2 General Stress | Regression | -0.53 | [-1.42, 0.36] | .241 |
| U.S. (vs Australia) | T2 General Stress | Regression | -0.43 | [-1.29, 0.44] | .334 |
| Age | T2 Alcohol Frequency | Regression | 0.00 | [-0.01, 0] | .172 |
| Pre-COVID Frequency | T2 Alcohol Frequency | Regression | -0.05 | [-0.16, 0.06] | .363 |
| Pre-COVID Quantity | T2 Alcohol Frequency | Regression | 0.12 | [-0.07, 0.31] | .215 |
| U.K. (vs Australia) | T2 Alcohol Frequency | Regression | 0.14 | [-0.15, 0.43] | .340 |
| U.S. (vs Australia) | T2 Alcohol Frequency | Regression | -0.04 | [-0.34, 0.26] | .799 |
| Age | T2 Alcohol Quantity | Regression | -0.01 | [-0.01, 0] | .071 |
| Pre-COVID Frequency | T2 Alcohol Quantity | Regression | -0.04 | [-0.15, 0.08] | .535 |
| Pre-COVID Quantity | T2 Alcohol Quantity | Regression | 0.10 | [-0.13, 0.33] | .389 |
| U.K. (vs Australia) | T2 Alcohol Quantity | Regression | -0.10 | [-0.38, 0.18] | .483 |
| U.S. (vs Australia) | T2 Alcohol Quantity | Regression | -0.05 | [-0.33, 0.24] | .737 |
| Age | T3 General Stress | Regression | -0.02 | [-0.04, -0.01] | **.012** |
| Pre-COVID Frequency | T3 General Stress | Regression | 0.11 | [-0.24, 0.46] | .537 |
| Pre-COVID Quantity | T3 General Stress | Regression | 0.03 | [-0.66, 0.71] | .940 |
| U.K. (vs Australia) | T3 General Stress | Regression | 0.96 | [0.08, 1.84] | **.033** |
| U.S. (vs Australia) | T3 General Stress | Regression | 0.40 | [-0.5, 1.3] | .384 |
| Age | T3 Alcohol Frequency | Regression | 0.00 | [0, 0.01] | .186 |
| Pre-COVID Frequency | T3 Alcohol Frequency | Regression | 0.05 | [-0.07, 0.17] | .411 |
| Pre-COVID Quantity | T3 Alcohol Frequency | Regression | -0.04 | [-0.23, 0.15] | .687 |
| U.K. (vs Australia) | T3 Alcohol Frequency | Regression | 0.03 | [-0.28, 0.34] | .857 |
| U.S. (vs Australia) | T3 Alcohol Frequency | Regression | 0.16 | [-0.13, 0.46] | .281 |
| Age | T3 Alcohol Quantity | Regression | 0.00 | [0, 0.01] | .280 |
| Pre-COVID Frequency | T3 Alcohol Quantity | Regression | 0.02 | [-0.1, 0.15] | .695 |
| Pre-COVID Quantity | T3 Alcohol Quantity | Regression | 0.07 | [-0.11, 0.25] | .449 |
| U.K. (vs Australia) | T3 Alcohol Quantity | Regression | 0.10 | [-0.2, 0.4] | .519 |
| U.S. (vs Australia) | T3 Alcohol Quantity | Regression | 0.02 | [-0.26, 0.31] | .879 |
| T1 Alcohol Frequency | T1 General Stress | Covariance | 0.20 | [-0.22, 0.62] | .352 |
| T1 Alcohol Quantity | T1 Alcohol Frequency | Covariance | 0.90 | [0.73, 1.07] | **<.001** |
| T1 Alcohol Quantity | T1 General Stress | Covariance | 0.30 | [-0.15, 0.75] | .196 |
| T2 Alcohol Frequency | T2 General Stress | Covariance | 0.26 | [0.02, 0.5] | **.037** |
| T2 Alcohol Quantity | T2 Alcohol Frequency | Covariance | 0.42 | [0.3, 0.54] | **<.001** |
| T2 Alcohol Quantity | T2 General Stress | Covariance | 0.32 | [0.08, 0.56] | **<.001** |
| T3 Alcohol Frequency | T3 General Stress | Covariance | -0.03 | [-0.35, 0.29] | .840 |
| T3 Alcohol Quantity | T3 Alcohol Frequency | Covariance | 0.52 | [0.37, 0.68] | **<.001** |
| T3 Alcohol Quantity | T3 General Stress | Covariance | 0.04 | [-0.25, 0.34] | .775 |
| T1 General Stress | T1 General Stress | Variance | 9.77 | [8.12, 11.41] | **<.001** |
| T1 Alcohol Frequency | T1 Alcohol Frequency | Variance | 1.04 | [0.86, 1.22] | **<.001** |
| T1 Alcohol Quantity | T1 Alcohol Quantity | Variance | 1.05 | [0.88, 1.22] | **<.001** |
| T2 General Stress | T2 General Stress | Variance | 6.30 | [5.06, 7.55] | **<.001** |
| T2 Alcohol Frequency | T2 Alcohol Frequency | Variance | 0.62 | [0.48, 0.76] | **<.001** |
| T2 Alcohol Quantity | T2 Alcohol Quantity | Variance | 0.57 | [0.45, 0.7] | **<.001** |
| T3 General Stress | T3 General Stress | Variance | 6.20 | [4.93, 7.47] | **<.001** |
| T3 Alcohol Frequency | T3 Alcohol Frequency | Variance | 0.72 | [0.56, 0.87] | **<.001** |
| T3 Alcohol Quantity | T3 Alcohol Quantity | Variance | 0.63 | [0.47, 0.79] | **<.001** |

Model fit: *χ*^2^ (9) = 48.19, *p* < .001, CFI = 0.97, TFI = 0.76, RMSEA = 0.14, SRMR = 0.04

**Table S24**

*Results for cross-lagged panel model examining associations between loneliness and alcohol consumption frequency and quantity*

| Predictor | Outcome | Path type | *b* | 95% CI | *p* |
| --- | --- | --- | --- | --- | --- |
| T1 Loneliness | T2 Loneliness | Regression | 0.79 | [0.7, 0.89] | **<.001** |
| T1 Alcohol Frequency | T2 Loneliness | Regression | 0.52 | [-1.77, 2.81] | .657 |
| T1 Alcohol Quantity | T2 Loneliness | Regression | -0.10 | [-2.3, 2.1] | .929 |
| T1 Loneliness | T2 Alcohol Frequency | Regression | 0.00 | [0, 0.01] | .222 |
| T1 Alcohol Frequency | T2 Alcohol Frequency | Regression | 0.28 | [0.06, 0.5] | **.012** |
| T1 Alcohol Quantity | T2 Alcohol Frequency | Regression | 0.46 | [0.25, 0.67] | **<.001** |
| T1 Loneliness | T2 Alcohol Quantity | Regression | 0.01 | [0, 0.01] | .056 |
| T1 Alcohol Frequency | T2 Alcohol Quantity | Regression | 0.27 | [0.07, 0.47] | **.009** |
| T1 Alcohol Quantity | T2 Alcohol Quantity | Regression | 0.42 | [0.21, 0.62] | **<.001** |
| T2 Loneliness | T3 Loneliness | Regression | 0.80 | [0.72, 0.88] | **<.001** |
| T2 Alcohol Frequency | T3 Loneliness | Regression | -0.68 | [-2.66, 1.31] | .502 |
| T2 Alcohol Quantity | T3 Loneliness | Regression | -0.08 | [-1.96, 1.8] | .933 |
| T2 Loneliness | T3 Alcohol Frequency | Regression | -0.01 | [-0.01, 0] | .113 |
| T2 Alcohol Frequency | T3 Alcohol Frequency | Regression | 0.45 | [0.23, 0.67] | **<.001** |
| T2 Alcohol Quantity | T3 Alcohol Frequency | Regression | 0.24 | [0, 0.47] | **.049** |
| T2 Loneliness | T3 Alcohol Quantity | Regression | -0.01 | [-0.01, 0] | **.033** |
| T2 Alcohol Frequency | T3 Alcohol Quantity | Regression | 0.23 | [0.05, 0.41] | **.013** |
| T2 Alcohol Quantity | T3 Alcohol Quantity | Regression | 0.46 | [0.26, 0.66] | **<.001** |
| Age | T1 Loneliness | Regression | -0.14 | [-0.25, -0.04] | .007 |
| Pre-COVID Frequency | T1 Loneliness | Regression | -1.79 | [-3.73, 0.16] | .072 |
| Pre-COVID Quantity | T1 Loneliness | Regression | 2.92 | [-1.12, 6.95] | .156 |
| U.K. (vs Australia) | T1 Loneliness | Regression | 0.57 | [-3.83, 4.97] | .801 |
| U.S. (vs Australia) | T1 Loneliness | Regression | 2.13 | [-3, 7.25] | .416 |
| Age | T1 Alcohol Frequency | Regression | 0.00 | [-0.01, 0.01] | .509 |
| Pre-COVID Frequency | T1 Alcohol Frequency | Regression | 0.03 | [-0.11, 0.17] | .642 |
| Pre-COVID Quantity | T1 Alcohol Frequency | Regression | 0.11 | [-0.05, 0.27] | .185 |
| U.K. (vs Australia) | T1 Alcohol Frequency | Regression | 0.17 | [-0.18, 0.53] | .338 |
| U.S. (vs Australia) | T1 Alcohol Frequency | Regression | 0.43 | [0.07, 0.79] | **.021** |
| Age | T1 Alcohol Quantity | Regression | 0.00 | [0, 0.01] | .348 |
| Pre-COVID Frequency | T1 Alcohol Quantity | Regression | 0.11 | [-0.04, 0.26] | .145 |
| Pre-COVID Quantity | T1 Alcohol Quantity | Regression | -0.04 | [-0.23, 0.15] | .655 |
| U.K. (vs Australia) | T1 Alcohol Quantity | Regression | 0.17 | [-0.2, 0.54] | .374 |
| U.S. (vs Australia) | T1 Alcohol Quantity | Regression | 0.39 | [0.01, 0.77] | **.044** |
| Age | T2 Loneliness | Regression | -0.02 | [-0.1, 0.06] | .608 |
| Pre-COVID Frequency | T2 Loneliness | Regression | 0.30 | [-0.99, 1.59] | .651 |
| Pre-COVID Quantity | T2 Loneliness | Regression | 0.43 | [-1.21, 2.07] | .604 |
| U.K. (vs Australia) | T2 Loneliness | Regression | -3.49 | [-6.77, -0.21] | **.037** |
| U.S. (vs Australia) | T2 Loneliness | Regression | -3.48 | [-7.16, 0.21] | .064 |
| Age | T2 Alcohol Frequency | Regression | 0.00 | [-0.01, 0] | .198 |
| Pre-COVID Frequency | T2 Alcohol Frequency | Regression | -0.04 | [-0.16, 0.07] | .446 |
| Pre-COVID Quantity | T2 Alcohol Frequency | Regression | 0.11 | [-0.08, 0.3] | .255 |
| U.K. (vs Australia) | T2 Alcohol Frequency | Regression | 0.15 | [-0.14, 0.44] | .314 |
| U.S. (vs Australia) | T2 Alcohol Frequency | Regression | -0.06 | [-0.36, 0.25] | .717 |
| Age | T2 Alcohol Quantity | Regression | 0.00 | [-0.01, 0] | .161 |
| Pre-COVID Frequency | T2 Alcohol Quantity | Regression | -0.02 | [-0.13, 0.09] | .692 |
| Pre-COVID Quantity | T2 Alcohol Quantity | Regression | 0.08 | [-0.15, 0.31] | .478 |
| U.K. (vs Australia) | T2 Alcohol Quantity | Regression | -0.11 | [-0.39, 0.17] | .450 |
| U.S. (vs Australia) | T2 Alcohol Quantity | Regression | -0.08 | [-0.37, 0.21] | .586 |
| Age | T3 Loneliness | Regression | -0.05 | [-0.13, 0.03] | .206 |
| Pre-COVID Frequency | T3 Loneliness | Regression | 0.03 | [-1.25, 1.3] | .967 |
| Pre-COVID Quantity | T3 Loneliness | Regression | 0.48 | [-1.45, 2.41] | .626 |
| U.K. (vs Australia) | T3 Loneliness | Regression | 4.85 | [1.41, 8.28] | **.006** |
| U.S. (vs Australia) | T3 Loneliness | Regression | 4.06 | [0.74, 7.38] | **.016** |
| Age | T3 Alcohol Frequency | Regression | 0.00 | [0, 0.01] | .340 |
| Pre-COVID Frequency | T3 Alcohol Frequency | Regression | 0.04 | [-0.08, 0.16] | .490 |
| Pre-COVID Quantity | T3 Alcohol Frequency | Regression | -0.01 | [-0.19, 0.18] | .947 |
| U.K. (vs Australia) | T3 Alcohol Frequency | Regression | -0.03 | [-0.34, 0.28] | .866 |
| U.S. (vs Australia) | T3 Alcohol Frequency | Regression | 0.13 | [-0.17, 0.42] | .397 |
| Age | T3 Alcohol Quantity | Regression | 0.00 | [0, 0.01] | .493 |
| Pre-COVID Frequency | T3 Alcohol Quantity | Regression | 0.01 | [-0.11, 0.13] | .813 |
| Pre-COVID Quantity | T3 Alcohol Quantity | Regression | 0.10 | [-0.09, 0.28] | .311 |
| U.K. (vs Australia) | T3 Alcohol Quantity | Regression | 0.06 | [-0.24, 0.36] | .700 |
| U.S. (vs Australia) | T3 Alcohol Quantity | Regression | 0.00 | [-0.29, 0.28] | .992 |
| T1 Alcohol Frequency | T1 Loneliness | Covariance | 0.45 | [-1.53, 2.43] | .655 |
| T1 Alcohol Quantity | T1 Alcohol Frequency | Covariance | 0.91 | [0.73, 1.08] | **<.001** |
| T1 Alcohol Quantity | T1 Loneliness | Covariance | 0.43 | [-1.51, 2.36] | .664 |
| T2 Alcohol Frequency | T2 Loneliness | Covariance | 1.05 | [0.03, 2.06] | **.043** |
| T2 Alcohol Quantity | T2 Alcohol Frequency | Covariance | 0.42 | [0.3, 0.54] | **<.001** |
| T2 Alcohol Quantity | T2 Loneliness | Covariance | 0.43 | [-0.53, 1.4] | .376 |
| T3 Alcohol Frequency | T3 Loneliness | Covariance | 1.27 | [0.05, 2.49] | **.041** |
| T3 Alcohol Quantity | T3 Alcohol Frequency | Covariance | 0.51 | [0.36, 0.67] | **<.001** |
| T3 Alcohol Quantity | T3 Loneliness | Covariance | 1.44 | [0.25, 2.62] | **.017** |
| T1 Loneliness | T1 Loneliness | Variance | 191.56 | [161.7, 221.41] | **<.001** |
| T1 Alcohol Frequency | T1 Alcohol Frequency | Variance | 1.05 | [0.87, 1.23] | **<.001** |
| T1 Alcohol Quantity | T1 Alcohol Quantity | Variance | 1.06 | [0.89, 1.23] | **<.001** |
| T2 Loneliness | T2 Loneliness | Variance | 87.38 | [66.29, 108.48] | **<.001** |
| T2 Alcohol Frequency | T2 Alcohol Frequency | Variance | 0.62 | [0.48, 0.76] | **<.001** |
| T2 Alcohol Quantity | T2 Alcohol Quantity | Variance | 0.57 | [0.44, 0.69] | **<.001** |
| T3 Loneliness | T3 Loneliness | Variance | 91.71 | [62.54, 120.88] | **<.001** |
| T3 Alcohol Frequency | T3 Alcohol Frequency | Variance | 0.70 | [0.55, 0.86] | **<.001** |
| T3 Alcohol Quantity | T3 Alcohol Quantity | Variance | 0.62 | [0.46, 0.78] | **<.001** |

Model fit: *χ*^2^ (9) = 54.02, *p* < .001, CFI = 0.97, TFI = 0.75, RMSEA = 0.15, SRMR = 0.04

## STROBE Checklist

|  | Item No | Recommendation | Page No |
| --- | --- | --- | --- |
| **Title and abstract** | 1 | (*a*) Indicate the study’s design with a commonly used term in the title or the abstract | p.3 |
|  |  | (*b*) Provide in the abstract an informative and balanced summary of what was done and what was found | p.3 |
| Introduction | | | |
| Background/rationale | 2 | Explain the scientific background and rationale for the investigation being reported | p.5-8 |
| Objectives | 3 | State specific objectives, including any prespecified hypotheses | p.8 |
| Methods | | | |
| Study design | 4 | Present key elements of study design early in the paper | p.8 |
| Setting | 5 | Describe the setting, locations, and relevant dates, including periods of recruitment, exposure, follow-up, and data collection | p.8-10 |
| Participants | 6 | (*a*) Give the eligibility criteria, and the sources and methods of selection of participants. Describe methods of follow-up | p.9-10 |
|  |  | (*b*) For matched studies, give matching criteria and number of exposed and unexposed | N/A |
| Variables | 7 | Clearly define all outcomes, exposures, predictors, potential confounders, and effect modifiers. Give diagnostic criteria, if applicable | p.10-14 |
| Data sources/ measurement | 8* | For each variable of interest, give sources of data and details of methods of assessment (measurement). Describe comparability of assessment methods if there is more than one group | p.10-14 |
| Bias | 9 | Describe any efforts to address potential sources of bias | p.14 |
| Study size | 10 | Explain how the study size was arrived at | p.9 |
| Quantitative variables | 11 | Explain how quantitative variables were handled in the analyses. If applicable, describe which groupings were chosen and why | p.10-14 |
| Statistical methods | 12 | (*a*) Describe all statistical methods, including those used to control for confounding | p.13-14 |
|  |  | (*b*) Describe any methods used to examine subgroups and interactions | p.13-14 |
|  |  | (*c*) Explain how missing data were addressed | p.12 |
|  |  | (*d*) If applicable, explain how loss to follow-up was addressed | p.12 |
|  |  | (*e*) Describe any sensitivity analyses | p.14 |
| Results | | |  |
| Participants | 13* | (a) Report numbers of individuals at each stage of study—eg numbers potentially eligible, examined for eligibility, confirmed eligible, included in the study, completing follow-up, and analysed | p.15-20 |
|  |  | (b) Give reasons for non-participation at each stage | p.18 |
|  |  | (c) Consider use of a flow diagram | N/A |
| Descriptive data | 14* | (a) Give characteristics of study participants (eg demographic, clinical, social) and information on exposures and potential confounders | p.15-20 |
|  |  | (b) Indicate number of participants with missing data for each variable of interest | p.15-20 |
|  |  | (c) Summarise follow-up time (eg, average and total amount) | p.9 |
| Outcome data | 15* | Report numbers of outcome events or summary measures over time | p.15-20 |
| Main results | 16 | (*a*) Give unadjusted estimates and, if applicable, confounder-adjusted estimates and their precision (eg, 95% confidence interval). Make clear which confounders were adjusted for and why they were included | p. 21-34 & SM p.10-43 |
|  |  | (*b*) Report category boundaries when continuous variables were categorized | N/A |
|  |  | (*c*) If relevant, consider translating estimates of relative risk into absolute risk for a meaningful time period | N/A |
| Other analyses | 17 | Report other analyses done—eg analyses of subgroups and interactions, and sensitivity analyses | S1 p.24-42 |
| **Discussion** | | | |
| Key results | 18 | Summarise key results with reference to study objectives | p.35 |
| Limitations | 19 | Discuss limitations of the study, taking into account sources of potential bias or imprecision. Discuss both direction and magnitude of any potential bias | p.39-30 |
| Interpretation | 20 | Give a cautious overall interpretation of results considering objectives, limitations, multiplicity of analyses, results from similar studies, and other relevant evidence | p.35-42 |
| Generalisability | 21 | Discuss the generalisability (external validity) of the study results | p.39-42 |
| **Other information** | | | |
| Funding | 22 | Give the source of funding and the role of the funders for the present study and, if applicable, for the original study on which the present article is based | p.1 |

Page numbers refer to that of the main manuscript document unless specified as SM (supplementary material). The STROBE checklist was adapted and downloaded from <https://www.equator-network.org/reporting-guidelines/strobe/>
